# Understanding the interaction of upper respiratory tract infection with respiratory syncytial virus and *Streptococcus pneumoniae* using a human challenge model: a multicenter, randomized controlled study protocol

**DOI:** 10.1371/journal.pone.0325149

**Published:** 2025-07-01

**Authors:** Sanjita Brito-Mutunayagam, David O. Hamilton, Elena Mitsi, Carla Solórzano, Grace Li, Bruno Rocha De Macedo, Filora Elterish, Xinxue Liu, Kiarash Tanha, Rachel White, Angela Hyder-Wright, Bhumika Patel, Britta C. Urban, Conor Whelan, Hanane Trari Belhadef, Hannah Robinson, Emma Plested, Amber Thompson, Maria Lahuerta, Isis Kanevsky, Kena Swanson, Negar Aliabadi, Julie Catusse, Christian Theilacker, Bradford D. Gessner, Natalie I. Mazur, Christopher Chiu, Andrea M. Collins, Ben Morton, Maheshi N. Ramasamy, Daniela M. Ferreira

**Affiliations:** 1 Oxford Vaccine Group, Department of Paediatrics, University of Oxford, Oxford, United Kingdom; 2 Department of Clinical Sciences, Liverpool School of Tropical Medicine, Liverpool, United Kingdom; 3 Pfizer Vaccines, Pfizer Inc, New York, New York, United States of America; 4 Department of Pediatric Infectious Diseases and Immunology, University Medical Centre Utrecht, Utrecht, Netherlands; 5 Department of Infectious Disease, Imperial College London, London, United Kingdom; 6 University Hospital Liverpool Group, Liverpool, United Kingdom; George Washington University School of Medicine and Health Sciences, UNITED STATES OF AMERICA

## Abstract

**Background:**

*Streptococcus pneumoniae* (pneumococcus) and respiratory syncytial virus (RSV) are major causes of respiratory infections globally. Viral and bacterial co-infections are commonly observed in respiratory infections and there is evidence that these pathogens interact synergistically to evade host responses and lead to more severe disease. Notably, RSV seasonal outbreaks are associated with increased hospitalization and a subsequent peak in invasive pneumococcal disease cases, particularly in pediatric populations. Here, we summarize a protocol for a controlled human infection model aiming to evaluate pathogen interaction dynamics and immune responses in a combined pneumococcus and RSV model. The primary objective is to determine whether primary RSV challenge increases the risk of secondary pneumococcal colonization.

**Methods:**

This is an open-label, multi-center, randomized controlled human co-infection study, inclusive of a pilot phase. Individuals will be randomized to primary inoculation with either pneumococcus (serotype 6B) or RSV (subtype RSV-A) intra-nasally on day 0 followed by a reciprocal challenge on day 7. During pilot phase A up to 10 participants will be monitored in an in-patient facility for 7–10 days following RSV-A challenge. If there are no safety concerns, we will then progress to an outpatient phase where participants will self-isolate at home. Clinical samples to be taken from participants include nasal swabs and washes for pathogen detection; and nasal cells, nasal lining fluid, and blood samples to examine mucosal and systemic immune responses.

**Discussion:**

This work will lead to important scientific knowledge on the interaction and dynamics between pneumococcus and RSV. This knowledge could help inform pneumococcal and RSV vaccination strategies, particularly for groups at risk of developing severe pneumococcal and RSV disease.

**Trial registration:**

The study is registered on ISRCTN (The UKs Clinical Study Registry). DOI https://doi.org/10.1186/ISRCTN12036902

## Introduction

Lower respiratory tract infections (LRTIs) caused by viruses and bacterial pathogens account for a substantial burden of disease and death throughout the world. *Streptococcus pneumoniae* (pneumococcus, Spn) and respiratory syncytial virus (RSV) are recognized as major causes of LRTIs in both children and older adults [1–3].

There are more than 100 serotypes of pneumococcus. Currently licensed pneumococcal conjugate vaccines (PCVs, e.g., PCV13, PCV15 and PCV20) are targeted toward serotypes causing the most disease and antibiotic resistance [4]. Expanded valent PCVs including up to 20 serotypes have been licensed in different countries and jurisdictions for both pediatric and adult indications [5]. PCV use in adults varies globally and is not currently used routinely in the United Kingdom (UK) [2,4].

There are two major RSV subtypes, A and B. Both can cause severe disease [1]. Treatment is limited primarily to supportive care for adults and children. Ribavirin is an antiviral drug that has been licensed for RSV infection, however its effectiveness is not well established and is associated with toxicity [6]. Infection prevention, therefore, is paramount. For infants there are two licensed prophylactic monoclonal antibodies, palivizumab and nirsevimab [1,6]. Recent clinical developments include the approval of RSV vaccines for older adults and pregnant women [7,8]. An immunization schedule for older adults and for infants either by active vaccination or maternal passive immunization has recently been implemented in the UK [9].

RSV and pneumococcus are known to interact in the upper airways, promoting synergistic viral or bacterial invasion through host immune modulation, which in turn leads to more severe respiratory disease and complications [10–12]. In the United States of America (USA), seasonal increases in RSV infection rates during the winter in young children are strongly associated with subsequent increased hospital admissions due to invasive pneumococcal disease [10]. Data from Israel showed a correlation between lower rates of pneumonia in children <5 years old with decreased rates of RSV, influenza and human metapneumovirus during COVID-19 lockdowns, despite almost unchanged pneumococcal carriage rates and density, implying synergy between these pathogens in driving invasive pneumococcal disease [13]. In children, the nasopharyngeal load of Spn can be 15-fold higher in those with viral co-infection, including RSV, than those with bacterial community acquired pneumonia alone [14]. In older adults, a study from the USA found an increased odds ratio of 2.7 for pneumonia among patients with concomitant RSV versus influenza infection [15]. These findings suggest that respiratory virus infections (including RSV) aggravate the burden of pneumococcal disease. While the mechanism for this interaction is not fully known, there is evidence showing that pre-existing RSV infections increase pneumococcal adherence, colonization and shedding, decrease bacterial clearance, increase pneumococcal virulence, and modulate immune responses to bacteria [16–21].

Spn colonization may also conversely influence RSV replication. An *in-vitro* study in human monocyte-derived macrophages reported that Spn co-infection contributes to increased levels of CXCL-10, in the context of limited RSV replication [21,22]. In a mouse model, using a murine analog of RSV, co-infection with Spn enhanced nasal cytokine secretions and increased virus clearance [17,21]. Alternatively, data from another study of Spn/RSV co-infection, in human bronchial epithelial cells and cotton rats, demonstrated that prior pneumococcal colonization can increase RSV viral load [21,23]. However, as these studies were conducted in cell lines or animal models, the impact of pneumococcal colonization on RSV disease severity remains unclear, particularly as RSV viral load does not consistently correlate with disease severity [24].

Selective vaccination against either pneumococcus or RSV may impact transmission of the organism not targeted for vaccination as a result of divergent immune responses to pathogens during co-infection [25]. Indirect/off-target effects of PCV vaccination have been shown in randomized clinical trials, specifically reducing pneumonia hospitalizations caused by viruses [26]. Likewise, the widespread implementation of PCVs had considerable consequences for the rates of viral lower respiratory tract infections in infants and toddlers [11].

### Controlled human infection models

Controlled human infection models (CHIMs) have enormous potential to evaluate disease pathogenesis, identify correlates of protection, accelerate vaccine development, and test vaccination strategies. CHIMs entail the deliberate exposure of volunteers who are carefully selected (for low risk of severe or invasive infection) and exposed under rigorously controlled conditions, to a precise dose of a well characterized pathogen [27].

RSV-A CHIMs, using Memphis 37, RSV A2 and Maryland strains conducted in the UK and USA, demonstrate an excellent safety profile with attack rates of 50–70%, dependent on challenge dose and pre-existing RSV-specific serum antibodies [28,29]. Participants infected with RSV experience either no, or mild to moderate symptoms, enabling the model to be extended to older adults aged 60–75 years [30]. Vaccine efficacy results reported in a Phase 2A human challenge trial [31] have been similar to those observed in a subsequent Phase 3 field trial [7], highlighting the potential for RSV human challenge models to accelerate vaccine development [32].

In the pneumococcus CHIMs, 40–70% of the participants nasally inoculated with pneumococcus develop nasal colonization for 1–4 weeks [33–35]. The Experimental Human Pneumococcal Challenge (EHPC) program has been conducted safely in the UK since 2010. Over 2000 participants including adults with moderate asthma and a cohort of healthy older adults aged 50–84 have been inoculated to date without safety concerns [33,36]. These studies have provided extensive knowledge in pneumococcal colonization responses, correlates of protection and mucosal immunity [37–41]. The model was used to test PCV13 efficacy in a double-blinded randomized controlled trial (RCT) in adults, demonstrating a 78% relative risk reduction in colonization by pneumococcal serotype 6B (Spn6B) one-month post-vaccination [42]. The model was also used to test PCV13 in a double-blind RCT in Malawi demonstrating a vaccine efficacy against carriage of 62% [43].

Recent observations in pneumococcus CHIM studies revealed that asymptomatic RSV and rhinovirus infection led to increased risk of pneumococcal acquisition, with 88% and 66% participants, respectively, becoming colonized after Spn6B challenge versus 49% in the virus negative group [44]. Presence of RSV at the time of pneumococcal challenge increased subsequent pneumococcal carriage density by almost 2 logs compared to participants with no virus infection [44].

We have previously utilized the pneumococcal CHIM to explore interactions with live attenuated influenza vaccine (LAIV) as a safe model for upper respiratory tract infections with influenza [45–47]. Both RSV and pneumococcus CHIMS are well established individually with robust and consistent safety data [48,49]. For this study we will employ standardized operating procedures (SOPs) in use for more than 10 years to develop a safe co-infection model to understand the effect of respiratory viral/bacterial co-infection in the general population.

### Rationale

Since exposure to both pneumococcus and RSV is common, it is critical to understand the impact of co-infection and their interactions in humans [1,2]. This work will lead to greater understanding of the microbial interactions between pneumococcus and RSV, as well how co-infection modulates host immune responses to each pathogen. Vaccines to prevent both RSV and pneumococcus are now licensed. Results of this work may inform pneumococcal vaccination strategies and whether PCVs can confer protection against viral pneumonias, particularly for the groups who are at risk of developing severe pneumococcal and RSV disease, such as young children, at-risk groups and older adults. Results may also shed light on whether anti-viral drugs and vaccines (e.g., against influenza, RSV, SARS-CoV2) can indirectly protect against pneumococcal disease and reduce pneumococcal transmission.

## Materials and methods

### Study design

**Fig 1:**
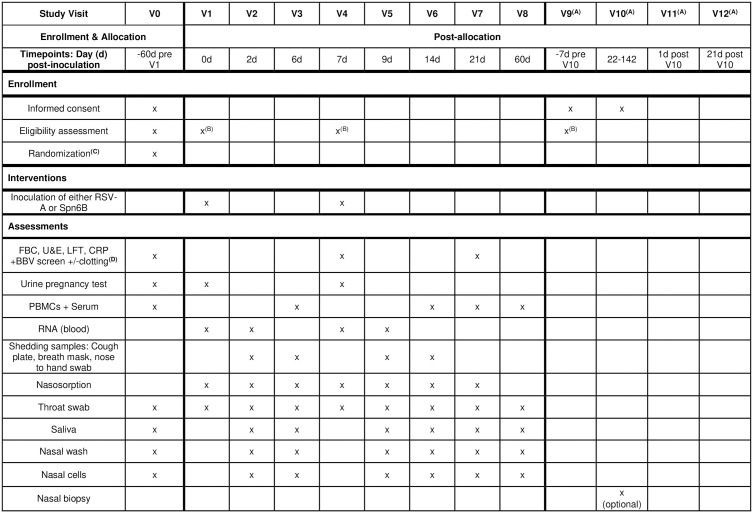
SPIRIT schedule of enrolment, interventions and assessments. (A) V9–V12 for participants consenting to the optional nasal biopsy; (B) Eligibility assessment includes assessing temporary exclusion prior to inoculation and nasal biopsy. (C) Randomization occurs before inoculation to allow participant preparation for self-isolation. (D) Blood-borne virus screen will only be done at screening and includes human immunodeficiency virus and hepatitis B and C. Clotting is only for participants undergoing nose biopsy. Abbreviations: BBV, blood-borne virus; CRP, C-reactive protein; FBC, full blood count; PBMCs, peripheral blood mononuclear cells; RNA, ribonucleic acid; RSV-A, respiratory syncytial virus A; Spn6B, *Streptococcus pneumoniae* 6B; U&Es, urea and electrolytes.

**Fig 2:**
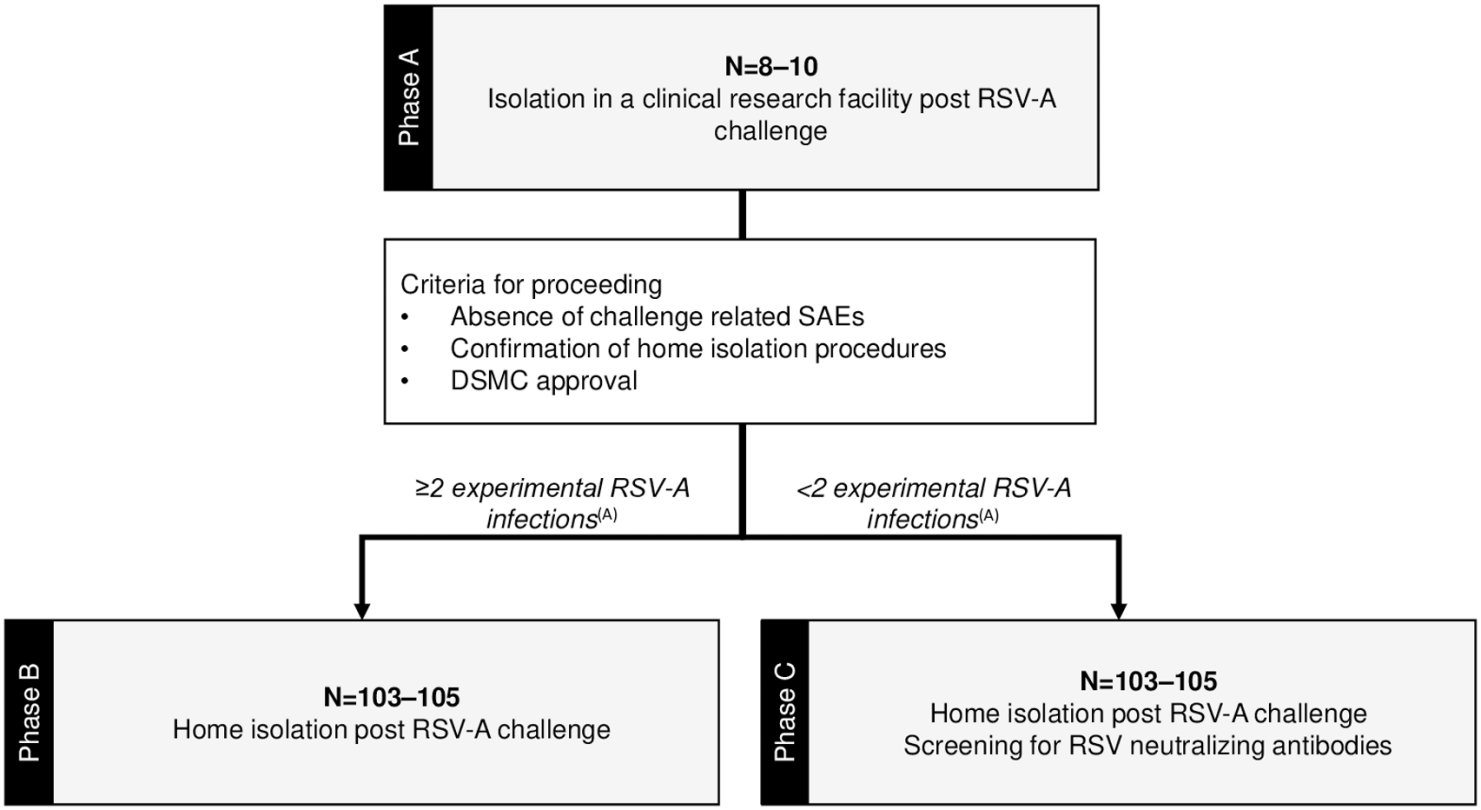
RESPECCT study phases. (A) If fewer than 2 participants are infected with RSV-A in phase A, then phase C will be conducted instead of phase B. Abbreviations: DSMC, data safety monitoring committee; RSV-A, respiratory syncytial virus; SAEs, serious adverse events.

This manuscript is written per protocol version 3.0, dated 30^th^ June 2024. The SPIRIT checklist and schedule of enrollment, interventions, and assessments is provided in S1 File and Fig 1 respectively, with the approved protocol provided in S2 File. Patients and members of the public were extensively consulted in the design of the study and creation of study documents. A focus group session was conducted to ensure the research questions and study design were informed by their experiences and preferences, especially regarding self-isolation procedures. This is an open-label, multi-center, randomized controlled human co-infection study with a pilot phase, conducted in Oxford and Liverpool, UK, with participant recruitment and data collection ongoing. The study will consist of two phases, a pilot phase A (isolation in a clinical research facility) and phase B or C (home isolation) as demonstrated in Fig 2. Participants will be required to isolate for up to 10 days post-RSV challenge, reduced to 7 days with a negative RSV result on day 7 either by point of care (POC) or nucleic acid amplification testing. Phase C will be conducted instead of phase B if fewer than 2 participants are infected with RSV in phase A. To increase RSV attack rates, participants will be pre-screened for RSV neutralizing antibody titers and only participants in the lowest 10^th^ percentile of antibody titers will be included in phase C.

### Recruitment and eligibility

The study will recruit healthy adults aged 18–55 years. Multiple strict inclusion and exclusion criteria will be applied during the screening process to minimize the risk to participants and limit the risk of onward transmission to others. A summary of these criteria is provided in Table 1, and a full list is provided in S3 Table.

**Table 1:** Summary of inclusion and exclusion criteria.

Inclusion Criteria
• Healthy adults aged 18–55 (inclusive, at the time of consent) • Fluent spoken English • Capacity to provide written informed consent in English • Females of childbearing potential with a negative urine pregnancy test at screening and willing to practice adequate contraceptive measures • Willing to provide their household contacts with the Close Contact Screening Information Letter • For Phase C, RSV neutralizing antibody titre in the lowest 10^th^ percentile of participants
**Exclusion Criteria**
• Research participant in another study • Unable to travel to outpatient clinic for visits during phase B/C after RSV challenge (for up to 10 days or 7 days if negative for RSV) without using public transport • Unable to wear a fluid resistant surgical mask **•** Live vaccination within four weeks and no previous pneumococcal or RSV vaccination • Allergy to beta lactam antibiotics • Medical history leading to increased risk of severe infection • Current issues such as upper respiratory tract infections or any uncontrolled conditions • Any major pneumococcal illness or pneumonia requiring hospitalization in the last 10 years • Medication that may affect the immune or coagulation systems or nasal biopsy procedure • Female participants who are pregnant, lactating or intending to become pregnant • Direct caring role or share living accommodation with individuals at risk from infection • Health-care worker • Current or ex-smoker in the last 6 months • Previous significant smoking history • Regularly drinks ≥3units/day (male) or ≥2units/day (female) • Regularly uses recreational drugs • Significant mental health disorders • Overseas travel planned during 21-day period following first inoculation • Participants full blood count results do not meet the required criteria on screening bloods • Any other issue which, in the opinion of the study staff, may affect the study or participant

Potential participants will be given a participant information sheet containing detailed study information and asked to complete an initial online and/or telephone questionnaire before they are invited for a screening and consent visit. Written informed consent and eligibility assessment will be done by the research team. Participants will be separately consented for their samples to be transferred to the Oxford Vaccine Centre (OVC) Biobank. Participants will be reimbursed for their time, travel, and inconvenience.

### Study procedures

After eligibility assessment, participants will be randomized 1:1 to either receive Spn6B inoculation at day 0 followed by RSV-A inoculation at day 7 or RSV-A inoculation at day 0 followed by Spn6B at day 7. A computer-generated randomization list will be prepared by the study statistician and will be stored on a secure electronic portal with restricted access. Random block sizes (2 and 4) will be used. To maintain a balance in phase A (equal number of participants in each arm), participants who are randomized but not inoculated are not enrolled in the study and can be replaced. The replacement participants will be allocated to study arms and not randomized. There will be no replacement in phase B/C. For phase B, randomization will be stratified based on the participant's sex (male/female) to account for differences in the likelihood of Spn colonization between sex [50]. Since this is an open-label study, participants and the clinical staff will be unblinded to randomization arms. However, the laboratory team conducting immunological assays will be blinded to participant allocation when processing samples for outcome measure assessment.

Participants will be considered enrolled to the study at the point of first challenge and all participants will be followed up for 60 days. The full schedule of enrollment, interventions, and assessments is shown in Fig 1. A subset of participants (up to 40) will also be offered an optional nasal biopsy procedure. Nasal biopsies will be collected from the inferior turbinate and/or postnasal nasopharynx-associated lymphoid tissue.

### Challenge procedures

Before RSV challenge, frozen aliquots will be thawed at 37°C and diluted to reach the desired dose of 1x10^4^ plaque forming units of the RSV-A inoculum [30]. Before Spn challenge, frozen aliquots will be thawed at room temperature and diluted to reach the desired dose of 80,000 colony forming units/100μl per naris [28]. The inoculum will be administered with the participant seated in a semi-recumbent position. Using a micropipette, 0.1 ml of pneumococcus or RSV-containing fluid will be instilled into each nostril. This will be done slowly with sufficient interval between each inoculation to ensure maximum contact time between the nasal and pharyngeal mucosa. After inoculation, the participant will remain in this position for up to 15 or 30 minutes for pneumococcus or RSV inoculation, respectively, and will be asked not to wash or blow their noses. Research staff performing bacterial and viral inoculation will wear full personal protective equipment including gloves, gown, safety goggles and fluid resistant surgical masks*.*

Following challenge administration, participants will be instructed to complete an online e-diary to record oral temperatures, and to describe any symptoms [51,52] or the use of medications for 21 days. After the first inoculation participants will be given a safety pack containing a safety information leaflet, respiratory precautions leaflet, and a backup paper diary (these documents are provided in S4 File). Oral amoxicillin (500 mg, three times daily for 3 days) will be provided to all participants after the first challenge. Participants may be advised to take antibiotics if there is clinical suspicion of a pneumococcal or other bacterial infection, they are unwell and unable to contact the research team, or remain colonized at the end of the 21-day follow-up period. Participants can call a delegated clinician 24/7 if they are concerned about their health.

### Challenge agents

The RSV-A challenge stock intended for use in this study is RSV-A Memphis 37, provided by Imperial College London and Duke University. The stock was produced at Charles River Laboratories, Malvern, PA, LOT: RSV070504. The strain has been developed specifically for human challenge trials and has been used in RSV CHIMs with no reported serious adverse events [12,15]. The virus was isolated from a child with severe RSV infection in the USA in 2007 and prepared according to current Good Manufacturing Practice in human Vero cells.

The pneumococcal inoculum used in this study is Spn6B BHN418 strain. Spn6B stocks are being produced and provided by Liverpool School of Tropical Medicine, LOT: SP-240919 and AH-050324. Spn6B strains will be grown to mid-log phase and stored in aliquots of glycerol-enriched media at –80°C. The preparation process of the bacterial stock is subject to independent identification, purity confirmation by whole genome sequencing, and assessment of antimicrobial sensitivity at a reference laboratory (UK Health Security Agency).

### Study objectives and measurements

The primary objective is to determine whether primary RSV-A challenge increases the risk of secondary Spn6B carriage using classical culture methods. Secondary objectives include determining the impact of primary RSV-A challenge and infection on secondary pneumococcal colonization dynamics (duration and density), the effect of primary Spn6B challenge and colonization on RSV infection dynamics, and assessment of symptoms and safety of the combined infection model. A full list of the primary, secondary, and exploratory objectives is outlined in S5 Table. Samples will be taken for safety, microbiological and immunological assays as shown in Fig 1. Participants will also be trained in taking self-samples to quantify inflammatory and infection dynamics (Table 2). To aid adherence to self-sampling, participants are provided with a guidance sheet and a planner.

**Table 2:** Participant self-sampling schedule.

Study Visit	V1					V4				
**Hours post inoculation**	**2**	**6**	**12**	**24**	**Daily until V4**	**2**	**6**	**12**	**24**	**Daily until V7**
Nasosorption	x	x	x	x		x	x	x	x	
Saliva	x	x	x	x		x	x	x	x	
Nasal swab	x	x	x	x		x	x	x	x	
Symptom diary				x					x	

Samples will be stored for the duration of the study and thereafter, if consent is given, be transferred to the OVC Biobank. Samples will be handled and processed in a containment level 2 facility. All samples will be stored at −80°C, except nasal cells and tissue, and peripheral blood mononuclear cells (PBMCs), which will be stored in liquid nitrogen.

### Pneumococcal and RSV detection

Colonization will be assessed by classical microbiology culture in nasal washes and serotype determined as Spn6B by latex agglutination. Colonization results will also be confirmed by multiplex quantitative polymerase chain reaction (qPCR) for *lytA* and *6BcpsA* genes on nasal wash pellets. A participant is considered positive for colonization if they test positive for pneumococcus at any nasal wash time point by either classical microbiology or molecular methods. Detection and viral load quantification of RSV-A will be performed on nasal wash and respiratory swabs by qPCR targeting the RSV N gene. A participant is considered RSV infected if they have positive samples at any two consecutive time points. Viral and bacterial shedding will be assessed using cough, breath, nose to hand, and saliva samples. Classical microbiology and qPCR for Spn and RSV detection will be performed on these samples to assess shedding of these pathogens.

### Immune measurements

Systemic and mucosal immune responses will be measured in relation to Spn carriage and density, as well as RSV infection status and viral load. Bead-based multiplex immunoassays will be used to quantify cytokine responses in the serum and mucosa. We will measure virus-specific and pneumococcal serotype-specific antibody responses using bead-based multiplex immunoassays and enzyme-linked immunosorbent assays to measure responses to RSV and Spn antigens. Viral neutralization and bacterial agglutination assays will be performed to measure functional antibody quality. Cellular responses including viral and pneumococcal specific T-cell responses (both CD4+ and CD8+), and levels of memory B cells will be assessed using spectral flow cytometry.

### Genetic measurements

Whole blood and nasal cells will be collected in PAX gene tubes and CTL-Cryo ABC media, respectively, until further analysis. Bulk RNA sequencing will be applied to cryopreserved whole blood samples to determine gene expression and regulation in response to challenge, whereas nasal cell transcriptome analysis will be assessed either at the single cell level using the 10x genomics platform or at the bulk level using bulk RNA sequencing. Freshly collected nasal biopsy samples will be bathed in isopentane on dry ice, followed by snap freezing in liquid nitrogen to prevent RNA degradation and avoid crystal formation. Cryopreserved nasal biopsy samples will be analyzed on spatial transcriptome platforms, such as Cell Dive, to characterize the nasal cell gene alterations to co-infection and their spatial location within the nasal tissue.

### Safety measurements

All adverse events (AEs) occurring from the first challenge visit to 21 days post-challenge will be recorded and graded. All solicited AEs will be automatically assumed to be related to challenge whereas all unsolicited AEs will undergo causality assessment in relation to the study interventions, depending on when they are reported (Table 3 provides the grading criteria). Laboratory results and vital signs will also be graded for severity (see SI Table 6), with clinically significant results reported as adverse events.

**Table 3:** Grading of solicited and unsolicited adverse events.

GRADE 0	None
GRADE 1	Mild: Transient or mild discomfort (< 48 hours); No interference with activity; No medical intervention/therapy required.
GRADE 2	Moderate: Mild to moderate limitation in activity – some assistance may be needed; no or minimal medical intervention/therapy required.
GRADE 3	Severe: Marked limitation in activity, some assistance usually required; medical intervention/therapy required.
GRADE 4	Potentially Life-threatening: Requires assessment in accident & emergency or hospitalization.

Serious adverse events (SAEs) will be reported from the time of enrollment until the completion of the study and reported to the DSMC, sponsor and funder within 24 hours of notification. Adverse events of special interest (AESIs) include invasive pneumococcal disease; severe RSV infection requiring hospitalization; pneumococcal pneumonia; otitis media or pneumococcal meningitis; virologically confirmed transmission of RSV-A to a household contact; and AEs requiring a physician visit or Emergency Department visit which, in the opinion of the study staff, are related to the challenge with RSV-A or Spn6B.

### Sample size

The effective sample size will be 90 (including participants who completed phase A). After adjusting for an attrition of 20%, the study will recruit a total of up to 113 participants. Sample size was determined based on 50% experimental Spn carriage in our EHPC program. During this EHPC program, we observed that 5/6 participants in whom asymptomatic RSV infection (confirmed by nasal PCR) were identified prior to pneumococcal challenge subsequently developed experimental pneumococcal carriage. Based on these data, and using a two-sided proportions power calculation test (parameters: alpha = 0.05 and 80% power), 45 participants will be required to demonstrate a difference of 30% Spn carriage in the two arms.

### Statistical analysis

The consolidated standards of reporting trials (CONSORT) will be used to report the results. Primary outcome (presence/absence of experimental Spn6B) will be summarized by frequencies and proportions by study arms. Data will be analyzed using a generalized linear model with presence/absence of Spn6B being dependent variable and randomization stratification factor (i.e., study site, and sex) and study arm included as independent variables. The risk ratio and rate difference together with their 95% confidence intervals will be estimated. Data from the pilot phase will be included within the analysis of primary outcome. The per-protocol population, will be used for the evaluation of primary outcome, defined as those who have consented to the study and met all study criteria, successfully challenged with Spn6B and RSV-A, three nasal wash data points within 14 days post first inoculation, no prohibited medications during the study, completed 7 days follow up following second inoculation and undergone throat swab POC testing for RSV and SARS-CoV-2.

### Ethical and safety considerations

#### Ethical approval, regulation, and governance.

The study will be conducted in accordance with the principles of the Declaration of Helsinki and in line with Good Clinical Practice guidelines. Ethical approval was obtained from East of England-Cambridge South Research Ethics Committee on 19^th^ October 2023; reference 23/EE/0219. Risk assessment for clinical and laboratory processes were approved by relevant committees from the University of Oxford (study sponsor) and the Oxford University Hospital NHS Foundation Trust. The University has a specialist insurance policy in place which would operate in the event of any participant suffering harm because of their involvement in the research.

An independent data safety monitoring committee (DSMC) consisting of an experienced group of infectious disease clinicians, scientists and a statistician will be appointed to provide real-time oversight of safety and trial conduct. Safety data from phase A will be reviewed prior to progression to Phase B/C. Additional DSMC reviews will occur regularly through the study.

#### Risks.

The general risks to participants in this study are associated with symptomatic upper respiratory tract infection, a very low risk of invasive pneumococcal disease or severe RSV infection, and discomfort associated with blood or upper respiratory tract sampling.

These risks will be greatly minimized by the selected challenge strain, careful participant selection and education, daily monitoring of symptoms and provision of standby antibiotics to reduce time-to-treatment. Participants who remain Spn colonized at Day 21 will also be advised to take a 3-day course of amoxicillin.

Combined bacterial and viral inoculation is novel for a CHIM, leading to the implementation of the additional safety measure of a pilot phase A incorporating inpatient confinement period of up to 10 days. During this time, participants will have immediate access to a healthcare professional 24/7 in the event of illness. Stopping rules for individual participants will apply, potentially halting progress to the second challenge if participants develop grade 3 or higher adverse events considered related to the challenge.

To mitigate any potential risk of spreading RSV or pneumococcus to vulnerable groups in the community, we will discuss these risks with the participants and exclude anyone with close physical contact with at risk individuals (see exclusion criteria). Multiple EHPC studies have demonstrated that there is extremely low risk of bacterial transmission between study participants and their close contacts. Home self-isolation procedures will be based on existing and extensively tested UKHSA procedures, utilized for the management of SARS-CoV2 positive individuals at the height of the pandemic. Throughout the possible infectivity period, participants will be taught to follow stringent respiratory precautions to minimize risk of transmission. Household contacts will also be offered screening for RSV infection. Nasal biopsies will be obtained by experienced ear, nose and throat surgeons in a hospital outpatient setting, and participants will be followed up for safety for 3 weeks post procedure.

### Data access and management

Consent will be documented on paper and recorded on the study database. All study data will be recorded on an electronic database designed for the study. The database will have a pre-defined data types and logic checks for real-time electronic data validation. Data collection and storage will be inspected throughout the study by the data management and monitoring teams. Participant data will be de-identified, other than for uses the participant has specifically consented to. Any electronic databases and documents with participant identifying details will be stored securely and will only be accessible by study staff.

## Discussion

This novel co-infection model will provide unique insights into the interactions between bacteria and viruses in the upper respiratory tract and the subsequent induction of mucosal and systemic immune responses. This is the first study to establish RSV in an ambulatory CHIM model, facilitating future work to test RSV vaccines or other pharmaceutical interventions without the need for restrictive and costly inpatient studies. While both RSV and Spn have previously been used in separate CHIMs, this will be the first study deliberating utilizing sequential challenge to establish co-infection and investigate the impact of each organism on the risk of colonization/infection with the other. Dissemination of findings is planned for publication in peer-reviewed journals and at international scientific conferences. In addition, we will produce a lay report of our findings, which will be made available to all participants.

While CHIM studies provide important insights into pathophysiology, they have certain limitations. Importantly, CHIM studies prioritize safety of the participants and thus only healthy adult volunteers will be recruited to RESPECCT. Results may therefore not be fully generalizable or representative of populations (young children and elderly) most at risk of severe infection. In the study participants will be challenged with a pre-specified bacterial serotype and viral strain at a single time point at a standard inoculum dose. This differs from natural infection events which likely involve multiple exposures to different pathogen strains at various doses and in prolonged time frames. Furthermore, it is possible that antibiotic treatment of symptomatic participants may impact on some microbiological and immunological outcomes.

This study aims to enhance our understanding of the synergy between respiratory bacterial viral co-infections and how this affects disease burden, transmission, and nasal immunity. By doing so, it may help determine whether interventions and vaccinations could be utilized to provide indirect protection, with an ultimate aim of reducing the global mortality and morbidity associated with pneumonia.

## Supporting information

S1 File Reporting checklist; SPIRIT checklist. (DOCX)

S2 File Protocol approved by the ethics committee. (PDF)

S3 Table RESPECCT inclusion and exclusion criteria. (DOCX)

S4 File RESPECCT safety pack documents. (PDF)

S5 Table Primary, secondary, and exploratory objectives. (DOCX)

S6 Table Adverse events grading. (DOCX)
