# Supplementary material for: Understanding the interaction of upper respiratory tract infection with respiratory syncytial virus and Streptococcus pneumoniae using a human challenge model: a multicenter, randomized controlled study protocol
Source: PLoS One. 2025 Jul 1;20(7):e0325149. doi: 10.1371/journal.pone.0325149 (PMC12212582; doi:10.1371/journal.pone.0325149)
Supplement: S2 File — (PDF) [file pone.0325149.s002.pdf]

[redacted]

Study title: *Understanding how two common respiratory infections interact in the nose of healthy adults: Respiratory Syncytial Virus and Streptococcus pneumoniae*

Short title: ***RE**spiratory syncytial virus and **S. pnE**umoniae **C**hallenge **C**oinfection s**T**udy (RESPECCT)*

Internal Reference Number: OVG2023/04
Study Code: RESPECCT
Research Ethics Reference: 23/EE/0219
IRAS Project ID 327739
Date and Version number: 30 July 2024, V3.0

Chief Investigator: Prof Maheshi Ramasamy
Principal Investigators: Prof Maheshi Ramasamy, Dr Ben Morton, Dr Andrea Collins
Lead Scientific Investigator: Prof Daniela Ferreira
Sponsor: University of Oxford
Funder: Pfizer Inc.

Chief Investigator Signature

Statistician Signature

None of the investigators declare any conflicts or potential conflicts of interest.

[redacted]

Protocol Signature Page

RESPECCT Version XX, Date XXX

The undersigned has read and understood the trial protocol detailed above and agrees to conduct the trial in compliance with the protocol.

**Principal Investigator** (Please print name)	**Signature**	**Site name or ID number**	**Date**
**Principal Investigator** (Please print name)	**Signature**	**Site name or ID number**	**Date**
**Principal Investigator** (Please print name)	**Signature**	**Site name or ID number**	**Date**

Following any amendments to the protocol, this page must be updated with the new protocol version number and date and re-signed by the site PI.

1. Key contacts 7
2. Lay Summary 11
3. Synopsis 13
4. Abbreviations 15
5. Background and rationale 17
5.1 Controlled Human Infection Models (CHIM) for Vaccine Research 18
5.2 Respiratory virus co-infections studies and alterations in mucosal immunity 18
5.3 *Streptococcus pneumoniae* serotype selection 19
5.4 RSV strain selection 19
6. Objectives and outcome measures 21
6.1 Primary aim 21
6.2 Secondary aims 21
6.3 Exploratory aims 21
6.4 Definitions 21
7. Study Design 24
7.1 Phase A 24
7.2 Phase B 24
7.3 Phase C 25
8. Participant identification 25
8.1 Study participants 25
8.2 Inclusion criteria 25
8.3 Exclusion criteria 26
8.4 Temporary exclusion criteria 28
9. Study procedures 29
9.1 Recruitment 29
9.2 Screening and eligibility assessment 30
9.3 Informed Consent 30
9.4 Consent of household close contacts for screening 31
9.5 Randomisation 32
9.6 Blinding 32
9.7 Study interventions, comparators and study procedures 33
9.8 Preparation of Spn6B challenge agent 36
9.9 Preparation of RSV challenge agent 37
9.10 Inoculation 38
9.11 Baseline assessments 41
9.12 Inpatient observation – phase A 41

9.13 Subsequent visits 41
9.14 Sample Handling 44
9.15 Early discontinuation / withdrawal of participants 44
9.16 End of Trial definition 44
10. Study interventions 44
10.1 Investigational Medicinal Products (IMP) 44
10.2 Concomitant medications 45
10.3 Assessment of compliance 45
10.4 Post study interventions 45
11. Risk Assessment and Mitigation 45
11.1 Risk to participant safety 47
11.2 Risk to participant contacts 50
11.3 Risk to researchers 50
12. Safety reporting 50
12.1 Definitions 50
12.2 Grading 51
12.3 Causality 52
12.4 Procedure for collecting and recording of adverse events 52
12.5 Recording and reporting of serious adverse events (SAEs) 54
12.6 Adverse Events of Special Interest (AESI) 54
12.7 Development Safety Update Reports (DSURs) 55
12.8 Stopping rules 55
12.9 Data and Safety Monitoring Committee (DSMC) 55
13. Statistics and Analysis 56
13.1 Statistical Analysis Plan 56
13.2 Populations for analysis of primary and secondary endpoints 56
13.3 Analysis of demographics and baseline characteristics 56
13.4 Data Summaries 56
13.5 Description of statistical methods 56
13.6 Analysis of primary outcome 57
13.7 Analysis of secondary outcomes 57
13.8 Analysis of exploratory outcomes 58
13.9 Sample size determination 58
13.10 Decision points 58
13.11 The Level of Statistical Significance 58
13.12 Procedure(s) to Account for Missing or Spurious Data 59

13.13 Procedures for Reporting any Deviation(s) from the Original Statistical Plan....................59
14. Data Management 59
14.1 Data integrity 59
14.2 Data storage....................59
14.3 Source data 60
14.4 Access to data 60
14.5 Data recording and record keeping 60
15. Quality assurance procedures 61
15.1 Risk assessment 61
15.2 Study monitoring 61
15.3 Study committees 61
Study Management Group (SMG): 61
Trial Steering Committee (TSC):....................62
Data and Safety Monitoring Committee (DSMC):....................62
16. Protocol deviations 62
16.1 Audit and Inspection 62
17. Serious breaches....................62
18. Ethical and regulatory considerations 63
18.1 Declaration of Helsinki 63
18.2 Guidelines for good clinical practice....................63
18.3 Approvals 63
18.4 Other ethical considerations....................63
18.5 Reporting....................63
18.6 Transparency in research....................63
18.7 Participant confidentiality....................64
18.8 Expenses and benefits 64
19. Finance and insurance 64
19.1 Funding....................64
19.2 Insurance....................65
19.3 Contractual arrangements....................65
20. Publication policy....................65
20.1 Publications....................65
20.2 Authorship....................65
21. Process for the generation of intellectual property 65
22. Archiving 65
23. References 66

Annex 1: Example diary card 71
Annex 2: Grading the severity of self-reported Adverse Events (challenge period) 72
Annex 3: Grading the severity of visit-observed Adverse Events 73
Annex 4: Grading the severity of laboratory Adverse Events 73
Annex 5: Amendment History 74

1. Key contacts

**Chief Investigator and SMG**	**Prof Maheshi Ramasamy**<br>Consultant Physician, Oxford University Hospitals NHS Foundation Trust<br>Associate Professor, Oxford Vaccine Group, University of Oxford,<br>Oxford Vaccine Group, University of Oxford, Centre for Clinical Vaccinology and Tropical Medicine (CCVTM), Churchill Hospital, Oxford, OX3 7LE, United Kingdom<br>[redacted]
**Principal Investigators and SMG**	**Site 1:** PI<br>Prof Maheshi Ramasamy. Consultant Physician, Oxford University Hospitals NHS Foundation Trust. Oxford Vaccine Group, University of Oxford, Centre for Clinical Vaccinology and Tropical Medicine (CCVTM), Churchill Hospital, Oxford, OX3 7LE, United Kingdom<br>[redacted]<br><br>**Site 2:**<br>Co-PI<br>Dr Ben Morton. Consultant Physician, Liverpool University Hospital NHS Foundation Trust. Liverpool Vaccine Group, Liverpool School of Tropical Medicine, Accelerator Building, 1 Daulby Street, Liverpool, L7 8XZ<br>[redacted]<br><br>Co-PI<br>Dr Andrea Collins. Senior Clinical Lecturer in Respiratory Medicine, Liverpool School of Tropical Medicine. Honorary Consultant at University Hospital Aintree and Royal Liverpool and Broadgreen University Hospital. Liverpool Life Sciences Accelerator, 1 Daulby Street, Liverpool, L7 8XZ<br>[redacted]<br><br>**Site 3:** PI **(non-recruiting site)**<br>Prof Maheshi Ramasamy. Consultant Physician, Oxford University Hospitals NHS Foundation Trust. Oxford University Hospitals NHS Foundation Trust. John Radcliffe Hospital, Oxford OX3 9DU [redacted]

**Lead Scientific Investigator and SMG**	Prof Daniela Ferreira<br><br>Oxford Vaccine Group, University of Oxford, Centre for Clinical Vaccinology and Tropical Medicine (CCVTM), Churchill Hospital, Oxford, OX3 7LE, United Kingdom<br><br>Liverpool Vaccine Group, Liverpool School of Tropical Medicine, Accelerator Building, 1 Daulby Street, Liverpool, L7 8XZ<br><br>[redacted]
**Lead Statistician and SMG**	Dr Xinxue Liu<br><br>Oxford Vaccine Group, University of Oxford, Centre for Clinical Vaccinology and Tropical Medicine (CCVTM), Churchill Hospital, Oxford, OX3 7LE, United Kingdom<br><br>[redacted]
**Laboratory Lead and SMG**	Dr Elena Mitsi<br><br>Oxford Vaccine Group, University of Oxford, Centre for Clinical Vaccinology and Tropical Medicine (CCVTM), Churchill Hospital, Oxford, OX3 7LE, United Kingdom<br><br>[redacted]
**Co-Investigators (Clinical)**	Dr Grace Li<br><br>Clinical Fellow, Oxford Vaccine Group, University of Oxford, Centre for Clinical Vaccinology and Tropical Medicine (CCVTM), Churchill Hospital, Oxford, OX3 7LE, United Kingdom<br><br>[redacted]<br><br>Dr Sanjita Brito-Mutunayagam<br><br>Clinical Fellow, Oxford Vaccine Group, University of Oxford, Centre for Clinical Vaccinology and Tropical Medicine (CCVTM), Churchill Hospital, Oxford, OX3 7LE, United Kingdom<br><br>[redacted]<br><br>Dr Bruno Rocha de Macedo<br><br>Oxford Vaccine Group, University of Oxford, Centre for Clinical Vaccinology and Tropical Medicine (CCVTM), Churchill Hospital, Oxford, OX3 7LE, United Kingdom<br><br>[redacted]

	Dr Oliver Hamilton<br>Clinical Fellow, Liverpool Vaccine Group, Liverpool School of Tropical Medicine, Accelerator Building, 1 Daulby Street, Liverpool, L7 8XZ, United Kingdom<br>[redacted]<br><br>Mr Samuel Leong<br>Consultant ENT Surgeon, Liverpool University Hospitals NHS Foundation Trust<br>[redacted]<br><br>Ms Abigail Lamyman<br>Consultant ENT Surgeon, Oxford University Hospitals NHS Foundation Trust.<br>[redacted]
**Trial manager**	Dr Carla Solorzano-Gonzalez<br>Oxford Vaccine Group, University of Oxford, Centre for Clinical Vaccinology and Tropical Medicine (CCVTM), Churchill Hospital, Oxford, OX3 7LE, United Kingdom<br>[redacted]
**Collaborators**	Professor Chris Chiu<br>Professor of Infectious Diseases, Faculty of Medicine, Department of Infectious Disease, Imperial College, London.<br>[redacted]<br><br>Dr Matthew Lawson Coates<br>ENT Consultant, University of Cambridge<br>[redacted]
**Sponsor**	University of Oxford<br>Research Governance, Ethics & Assurance Team (RGEA)<br>Boundary Brook House<br>Churchill Drive<br>Headington<br>Oxford OX3 7GB<br>United Kingdom
**Funder**	**Pfizer Inc.**<br>235 East 42nd Street, NY 10017, USA

**Sites**	**Site 1:**<br>Oxford Vaccine Group, University of Oxford, Centre for Clinical Vaccinology and Tropical Medicine (CCVTM), Churchill Hospital, Oxford, OX3 7LE, United Kingdom<br>**Site 2:**<br>Liverpool Vaccine Group, Liverpool School of Tropical Medicine (LSTM), Liverpool Life Sciences Accelerator Building, 1 Daulby Street, Liverpool, L7 8XZ, United Kingdom<br>**Site 3:** **<u>non-recruiting site</u>**<br>Oxford University Hospitals NHS Foundation Trust. John Radcliffe Hospital, Oxford OX3 9DU

2. Lay Summary

Respiratory syncytial virus (RSV) and pneumococcus are two of the most common causes of respiratory infections that affect young children and elderly people. Although these bugs can independently cause severe infection, there is a growing body of evidence demonstrating that they can interact with each other and make disease worse.

RSV infection primarily affects the lungs and causes symptoms such as cough, fever, and difficulty breathing. It spreads through droplets when an infected person talks, coughs or sneezes. Pneumococcus, on the other hand, is a bacterial infection that affects the lungs and can cause fever, cough, chest pain, and shortness of breath. It is spread through direct contact with saliva or mucus of an infected person.

RSV and pneumococcus are particularly harmful in young children, elderly people, and those with weakened immune systems. When RSV and pneumococcus co-infect an individual, it can lead to increased pathogen load and risk of transmission of the bugs in the community. It can also increase disease severity and lead to complications such as asthma, bronchitis, and pneumonia. In addition, recent studies have shown that RSV may weaken the body's immune defences against pneumococcus, making it easier for the bacteria to invade and cause an infection.

The aim of this study is to understand how RSV and pneumococcus interact in the nose of healthy adult volunteers. Our group has developed innovative methods to collect fluid and cells to detect virus and bacterial infections as well as to understand the body's response at the site of infection – the nose. For pneumococcus, we have also developed a human infection challenge model to study responses to pneumococcal colonisation in the nose. This model, in which people are infected with small amounts of bacteria in their nose ("challenge"), allows us to understand why some people get exposed to the bacteria and do not develop infection whilst others do. It is also a useful tool to accelerate vaccine development. To date we have challenge with pneumococcus over 2000 participants and demonstrated these studies can be done safely. For RSV we will be adopting an established and safe RSV human infection model successfully used by researchers in both the US and the UK to improve our understanding of this infection and disease.

In this study we will combine these two safe and established human infection models to gain important knowledge on the co-infection. Participants will be randomised to receive either RSV or pneumococcal challenge first. After this, participants will receive the other infection seven days later. This means that a participant who receives RSV first will get pneumococcus second and a participant who received pneumococcus first will get RSV second. Our hypothesis is that we will see more pneumococcus nasal infection cases in those who receive RSV infection first. We will take samples throughout the study so that we can track and understand immune responses to infection challenge.

Safety will be paramount in this study. This will be the first study to combine the two safe and established models so we will ask participants to remain in our medical facility for up to 10 days after their RSV challenge. We will closely observe participants during this first phase of the study (inpatient only) and refine our processes to manage participants on an outpatient basis once co-challenge safety is established. In the outpatient phase, participants will be asked to self-isolate, as happened in the COVID-19 pandemic, for up to 10 days after RSV infection to reduce the risk of transmission to others. In this second phase (outpatients), participants will be asked to wear a facemask on their way to and

from the outpatient clinic and not to use public transport. We will work closely with participants so that they understand study requirements and will address their questions and concerns.

The knowledge generated in this project could be used to help develop new interventions such as anti-virus agents and drugs targeting the host body response. Data from our work will help authorities with decisions on immunisation policy (who gets which immunisations/therapies and when). This study is very timely with many new and improved vaccines/immunisations for RSV and pneumococcal infections coming to the market.

3. Synopsis

**Study Title**	Understanding how two common respiratory infections interact in the nose of healthy adults: Respiratory Syncytial Virus and *Streptococcus pneumoniae*
**Study Code**	RESPECCT
**Chief Investigator**	Prof Maheshi Ramasamy
**Sponsor**	University of Oxford
**Funder**	Pfizer Inc.
**Trial Design**	Open label randomised controlled trial
**Study Centres**	University of Oxford and Liverpool School of Tropical Medicine
**Study Participants**	Healthy adults aged 18-55 inclusive
**Planned study size**	113 participants will be recruited to allow for 90 participants to complete
**Inoculation timing**	Intranasal inoculation day 0 and day 7 (both nares)
**Study agents**	*Streptococcus pneumoniae* serotype 6B (Spn6B) BHN418 strain<br>Respiratory syncytial virus A (RSV-A) Memphis 37 strain
**Doses**	Spn6B: 80,000 colony forming units (CFU)/naris<br>RSV-A: 5,000 plaque forming units (PFU)/naris
**Follow-up duration**	60 days or up to 142 days if undergoing optional biopsy
**Sampling**	See visit schedule
**Study interventions**	Intranasal inoculation with Spn6B suspension<br>Intranasal inoculation with RSV-A suspension
**Primary objective**	To determine if RSV-A **challenge** increases the risk of secondary Spn6B experimental carriage using classical culture
**Outcome measures**	Spn6B in nasal washes detected by classical culture<br>RSV-A in nasal washes and nasal swabs detected by RT-qPCR
**Secondary objectives**	To confirm co-infection model safety for participants
	To determine if primary RSV-A **challenge** increases the risk of secondary Spn6B carriage using molecular methods
	To determine if primary RSV-A **infection** increases the risk of secondary Spn6B carriage
	To determine if primary RSV-A **challenge or infection** alters the density of secondary Spn6B carriage
	To determine if primary RSV-A **challenge or infection** alters the duration of secondary Spn6B carriage
	To determine if primary Spn6B **challenge** alters the risk of secondary RSV-A infection

	To determine if primary Spn6B **carriage** alters the risk of secondary RSV-A infection
	To determine if primary Spn6B **challenge or carriage** alters the viral load of secondary RSV-A infection (AUC of density over time)
	To determine if primary Spn6B **challenge or carriage** alters the duration of secondary RSV-A infection
	To determine if co-infection with RSV-A and Spn6B alter URTI and LRTI symptoms
	Longitudinal assessment of viral and bacterial shedding from the nose in both arms of the study
	To determine if primary Spn6B **carriage** increases the proportion of participants from whom live RSV-A can be isolated from their hands
	To determine if primary or secondary RSV-A **infection** increases the proportion of participants from whom live Spn6B can be isolated from their hands
	To determine if Spn6B **carriage** density correlates with bacterial shedding
	To determine if RSV-A nasal and throat viral load correlates with viral shedding
	To determine if the outpatient RSV-A challenge model is associated with any increased risk of infection in household contacts
**Exploratory objectives**	To evaluate the immunology of pneumococcal/viral interaction and the mechanisms through which one pathogen modulates immune responses to the second pathogen challenge

4. Abbreviations

AE	Adverse Event
AUC	Area Under the Curve
CAP	Community Acquired Pneumonia
CFU	Colony Forming Units
CHIM	Controlled Human Infection Model
CRF	Case Report Form
DBRCT	Double blinded randomized-controlled trial
DSMC	Data, Safety and Monitoring Committee
EHPC	Experimental Human Pneumococcal Carriage
ELISA	Enzyme-Linked Immunosorbent Assay
ELISpot	Enzyme-Linked Immune Absorbent Spot
ENT	Ear, nose and throat
EPR	Electronic Patient Record
FBC	Full Blood Count
GCP	Good Clinical Practice
GEE	Generalised estimating equations
GI	Gastrointestinal
GLMM	Generalised linear mixed models
GPQ	General practitioner's questionaries
hCoV	Human Coronavirus
ICF	Informed Consent Form
ICH-GCP	International Conference on Harmonisation of Good Clinical Practice
LAIV	Live attenuated influenza vaccine
LRTI	Lower respiratory tract infection
LSTM	Liverpool School of Tropical Medicine
MHRA	Medicines and Healthcare products Regulatory Authority (UK Regulator)
mRNA	Messenger ribonucleic acid
MTA	Material Transfer Agreement
NAAT	Nucleic Acid Amplification Test
NALT	Nasal Associated Lymphoid Tissue
NW	Nasal wash

OM	Otitis Media
PBMC	Peripheral Blood Mononuclear Cell
PCR	Polymerase Chain Reaction
PCV	Pneumococcal conjugate vaccine
PFU	Plaque-forming units
PHE	Public Health England
PIS	Participant Information Sheet
PPSV23	Pneumococcal polysaccharide vaccine 23
qPCR	Quantitative Polymerase Chain Reaction
RCT	Randomised-controlled trial
RNA	Ribonucleic Acid
RSV	Respiratory Syncytial Virus
RT-qPCR	Real-time qPCR
SAE	Serious Adverse Event
SMG	Study Management Group
SOP	Standardised Operating Procedure
TDS	Three time a day
TSC	Trial Steering Committee
URTI	Upper respiratory tract infection

5. Background and rationale

Lower respiratory tract infections (LRTI) caused by viruses and bacterial pathogens account for a substantial burden of disease and death throughout the world. *Streptococcus pneumoniae* bacterium (pneumococcus) and respiratory syncytial virus (RSV) are separately recognised as major causes of LRTIs in both children and older adults (1, 2).

There is increasing evidence that these pathogens interact in the upper airways, promoting synergistic viral and/or bacterial invasion through host immune modulation which in turn leads to more severe respiratory disease and complications. For example, seasonal increases in RSV infection rates during the winter in young children are strongly associated with subsequent increased hospital admissions due to invasive pneumococcal disease (3, 4).

There are more than 100 distinct serotypes of pneumococcus. Currently licensed pneumococcal conjugate vaccines (PCVs, e.g., PCV13) included within infant vaccine schedules are targeted toward the most invasive serotypes. Expanded valent PCVs including up to 20 serotypes are being licensed. PCV use in adults varies globally and is not currently used routinely in the UK.

There are two major RSV subgroups, A and B. Both cause severe disease and either of them can be dominant in a given season. As of March 4, 2023, there are no licensed RSV vaccines for adults but Phase 3 trial results have shown high levels of protection and RSV vaccines for older people and pregnant women are likely to be licensed by the end of 2023. Treatment is limited primarily to supportive care for adults and children. For infants there are two licenced monoclonal antibodies prophylaxis, palivizumab and nirsevimab.

Since pneumococcal colonisation and infection with RSV are both common (2, 5), it is critical to understand their interactions in humans. This information will allow a more effective design and implementation of interventions and vaccination strategies. Alterations in the mucosal microenvironment due to co-infection may be a critical determinant of disease and/or transmission of pathogens, with the order of host exposure to respiratory pathogens altering disease outcome through stimulation of divergent immune responses.

This work will lead to important scientific knowledge on the interaction and dynamics between pneumococcus and RSV, as well as on the mechanisms by which pneumococcus modulates host immune responses to the virus. This knowledge will inform pneumococcal vaccination strategies particularly for the groups who are at risk of developing severe pneumococcal and RSV disease, such as young children, at-risk groups and older adults. Vaccination against pneumococcus or RSV may impact on transmission of the other organism as a result of divergent immune responses to pathogens during co-infection.

Vaccination of the elderly and vulnerable population with PCV13 and PCV20 could be a prophylactic strategy to halt cases of severe respiratory infections in these high-risk groups. Given the evident indirect effect of PCV13 vaccine in protecting unvaccinated individuals in the population, implementation of newly developed pneumococcal vaccines with expanded serotype coverage, such as PCV20, would be highly recommended in childhood immunisation programmes globally.

In addition, the acquired knowledge could be utilised for the development of new interventions such anti-virus drugs and drugs targeting the host body response. Increasing our scarce knowledge on nasal immunity against respiratory viruses will also help future pandemic preparedness. Better understanding of respiratory infections synergy, transmission and associated nasal immunity can unlock the potential for indirect protection by vaccination to reduce mortality, morbidity and costs associated with pneumonia in at-risk groups in the UK and globally.

Ultimately, key questions in the field are whether anti-viral drugs and vaccines (e.g. against influenza, RSV, SARS-CoV2) can offer 'off target' protection against pneumococcal disease and whether PCV vaccines can offer similar protection against viral pneumonias.

5.1 Controlled Human Infection Models (CHIM) for Vaccine Research

Controlled human infection models (CHIMs) have enormous potential to study the pathogenesis of a disease, accelerate vaccine development and test vaccination strategies. CHIMs entail the deliberate exposure of volunteers, who are carefully selected and exposed under rigorously controlled conditions, to an infectious dose of human pathogens (6). CHIM trials have informed decision-making and vaccine policy worldwide. Two examples are the Vaxchora Cholera vaccine recently licensed by the FDA for travellers and the WHO recommendation of the Vi-tetanus toxoid conjugated *Salmonella typhi* vaccine which has now been given to millions of children worldwide.

RSV-A CHIMs using Memphis 37, RSV A2 and Maryland strains has been conducted in the USA and in the UK with an excellent safety profile with attack rates of 50-70% depending on challenge dose and pre-screening of participants based on pre-existing RSV-specific serum antibodies (7, 8). Participants who develop RSV infection post-challenge have only mild to moderate symptoms. The model has also been used in adults aged 60-75 years (9). Further, several therapeutics including potential vaccine candidates have been tested using the RSV CHIM (10-12). Vaccine efficacy results reported in a Phase 2A human challenge trial (13) have been similar to those observed in a subsequent Phase 3 field trial (14), highlighting the potential for RSV human challenge models to accelerate vaccine development (15). Outpatient RSV challenge studies are currently being conducted in the Netherlands (verbal communication with Prof Louis Bont, Utrecht UMC).

Over the past decade we have developed a unique CHIM with pneumococcus in which 40-70% of the participants nasally inoculated with pneumococcus develop nasal colonisation for 1-4 weeks (16-18). This Programme (Experimental Human Pneumococcal Colonisation [EHPC]) – has been running safely in the UK for thirteen years. Over 2000 participants including adults with moderate asthma and healthy adults aged 50-84 (16, 19) have been inoculated to date without safety concerns. The study team have gained extensive knowledge in pneumococcal infection responses, correlates of protection and mucosal immunity (20-25). This work has demonstrated that prior carriage boosts pneumococcus-specific IgG responses as well as cellular responses in the lungs and blood (20). Serotype-specific memory B cells were associated with protection from reacquisition of carriage following re-challenge studies (18, 20, 26).

The pneumococcal CHIM model has been used to test PCV13 conjugate vaccine efficacy in a double-blinded randomised-controlled trial in adults and demonstrated a 78% relative risk reduction in colonisation by Spn6B one month post vaccination (27). These results were consistent with paediatric field studies showing similar reduction in colonisation in vaccinated children (28). The study required only 100 participants and was completed within 9 months. The EHPC study team have recently developed a new pneumococcal CHIM using Spn3, which is associated with severe clinical manifestations and reduced antibody mediated clearance (29) and are testing PCV13 vs PPSV23 vs Placebo in a DBRCT (30).

5.2 Respiratory virus co-infections studies and alterations in mucosal immunity

We have previously shown that asymptomatic virus infection at the time of pneumococcus inoculation increased the risk of adults becoming colonised (31). We have now collected further data on 489 study participants screened in CHIM pneumococcus studies. These data show a strong association between the presence of certain viruses just prior to inoculation and the increased risk of pneumococcal colonization after nasal challenge (carriage+). All the participants were asymptomatic at the time of pneumococcal inoculation but 16.5% (n=81) were viral PCR nasopharyngeal swab positive (most commonly detected viruses were human coronavirus (hCoV), rhinovirus and RSV). Amongst those without viral infection (n=408), 50% became pneumococcal carriage positive versus 63% in those with virus detected. Rhinovirus and RSV-A were particularly associated with increased risk of pneumococcal colonization, 68% and 83% respectively, after pneumococcal challenge. For hCoV infection 55%

became pneumococcal carriage positive. hCoV and RSV-A viral infection also increased subsequent pneumococcal carriage density by almost 2 logs (manuscript in preparation).

We have used our CHIM to study interactions of pneumococcus and live attenuated influenza vaccine (LAIV) as a model for URTI with influenza (31-34). We demonstrated that the effect of pneumococcal colonisation on the outcome of infection and immune responses to influenza antigens is dependent on the order of pathogen exposure. Attenuated influenza infection prior to challenge with pneumococcus leads to exacerbated nasal inflammatory responses and impaired nasal cellular responses, particularly affecting monocytes, which was associated with increased pneumococcal bacterial carriage density (34). Surprisingly, pre-existing pneumococcal colonisation with pneumococcus at the time of attenuated influenza virus infection reduces nasal inflammatory responses leading to decreased nasal antibody responses, as well as lung cellular responses to influenza antigens (34). We have recently reported similar findings during infection with SARS-CoV2 in 400 patients admitted at the hospital and 100 healthcare workers with severe to moderate and mild viral infection, respectively (35). Pneumococcal colonisation at the time of SARS-CoV2 infection dampened virus antigen specific B and T cell levels as well as systemic and mucosal antibody responses (35). We postulate that pneumococcal colonisation may alter antigen presentation at the nasal epithelia reducing immune responses to subsequent virus infections. PCV vaccination, which reduces pneumococcal colonisation, could therefore have an indirect effect on protection against respiratory infections. Data in the literature also support this. Post hoc analysis of two Randomised-controlled trials (RCTs) found that individuals vaccinated with PCVs had 30–35% reduction in hospitalisations for the endemic human coronaviruses (hCoV OC43, and HKU1) associated with pneumonia in adults (36, 37) and LRTI in children (38). PCV13 vaccination in older adults was associated with a reduction of approximately 30% in COVID-19 disease, hospitalisation and death (34).

This work poses important questions on whether the synergy of respiratory infections with viruses and bacteria can lead to more severe disease, what nasal immune mechanisms are associated with transmission and whether transmission can be curbed by improved vaccination strategies.

The main focus of this study is to determine pathogen interactions and host immune responses to RSV and Spn during bacteria-virus co-infection. Both models are well established individually with robust and consistent safety data. For this study we will employ standardised operating procedures established for more than 10 years to develop a safe co-infection model, as our group seeks to understand the effect of respiratory viral/bacterial co-infection in the general population.

5.3 *Streptococcus pneumoniae* serotype selection

There are more than 100 distinct pneumococcal serotypes. PCV13 targets the 13 serotypes (1, 3, 4, 5, 6A, 6B, 7F, 9V, 14, 19A, 19F, 18C, and 23F) associated with high potential for invasive disease. For this study, we have selected Spn6B as a pneumococcal serotype associated with lower invasive potential. Observational evidence from a large population cohort study demonstrates that RSV-A infection is more likely to be associated with pneumococcal co-infection with typically less pathogenic serotypes such as Spn6B (39). Our group has previously established a safe and reproducible controlled human infection model with Spn6B, demonstrating 50% carriage rate with a single dose of 80,000 CFUs/naris (27).

5.4 RSV strain selection

The Respiratory Syncytial Virus (RSV) A controlled human infection model was first established in 2009 (8) and has been used extensively (with robust safety data) to explore the immunopathology of RSV-A infection, including recent findings describing the association of nasal mucosal neutrophilic infiltration in the nasal mucosa and their activation level with predisposition to development of

symptomatic viral infection (40). Similar to the EHPC model, participants are given RSV by nasal administration with respiratory mucosal and systemic (blood) samples taken to monitor immune responses.

We will use the RSV-A Memphis 37 strain, an inoculum developed specifically for human challenge. This virus has been used to infect over 350 healthy adults in studies in the UK (8, 41, 42) without reported serious adverse events. Infectivity in humans has been demonstrated for doses of 3-5 log PFU with 55.6% attack rate (125 out of 225 participants becoming infected with RSV) in the most recent studies. Virus stocks and SOPs for virus dilution for challenge are being provided in collaboration with Prof Chris Chiu (Imperial College London).

We will now leverage more than a decade of safe and successfully delivered controlled human infection models experience to combine these two models. This work is vital to understand the interaction between viral and bacterial infection in the respiratory tract. Specifically, we will seek to understand how pre-existing viral and bacterial colonisation potentiate co-infection with bacteria and viruses respectively. This study will increase our understanding of the immunopathology behind co-infection, identify correlates of protection and susceptibility to infection and inform how vaccination targeting pneumococci and RSV should be employed to maximise protection in the population.

6. Objectives and outcome measures

In this study we propose to evaluate pathogen interaction dynamics and immune responses in a combined pneumococcal (Spn) and RSV controlled human infection model.

6.1 Primary aim

To compare the Spn6B colonisation rates following Spn6B inoculation between participants with or without previous RSV-A challenge.

6.2 Secondary aims

1. To determine participant symptoms, AEs and safety after each challenge and combined infection model
2. To assess the effect of primary Spn6B challenge and colonisation on RSV infection dynamics (alterations in RSV infection symptoms, attack rates, duration of the infection and viral load, nasal shedding)
3. To assess the effect of primary RSV challenge and infection on pneumococcal colonisation dynamics (alterations in Spn Symptoms, attack rates, density and duration of carriage episode, nasal shedding).
4. To determine the parameters for outpatient delivery.

6.3 Exploratory aims

To evaluate the immunology of pneumococcal/viral interaction and the mechanisms through which one pathogen modulates immune responses to the second pathogen challenge.

6.4 Definitions

The following definitions and figure 1 have been included to provide a better understanding of the objectives of the study:

- RSV-A challenge: individuals experimentally nasally inoculated with RSV-A strain
- RSV-A infection: individuals experimentally nasally inoculated and who became infected with RSV-A (positive result in two consecutive days and in samples of same type, e.g. nasosorption/ swabs).
- Spn6B challenge: individuals experimentally nasally inoculated with pneumococcus serotype 6B
- Spn6B carriage: individuals experimentally nasally inoculated and who became colonised with pneumococcus serotype 6B (positive result at any day using nasal washes).

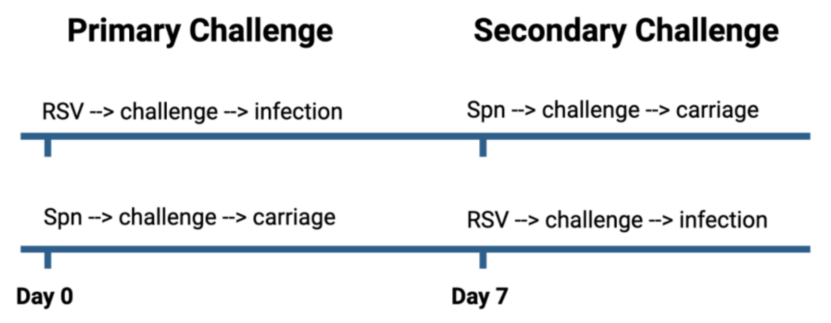

Figure 1: Representation of the definitions used for the study objectives.

Table 1: Objectives and outcome measures

Objectives	Outcome measures
**Primary study objectives**	
A. To determine if primary RSV-A **challenge** increases the risk of secondary Spn6B carriage using classical culture	Colonisation rates of Spn6B determined by classical culture
**Secondary objectives**	
1. To confirm co-infection model safety for participants	Absence of SAEs/AESIs relating to inoculation throughout the study<br>(see section 7.1)
2. To determine if primary RSV-A **challenge** increases the risk of secondary Spn6B carriage using molecular methods	Colonisation rates of Spn6B determined by RT-qPCR
3. To determine if primary RSV-A **infection** increases the risk of secondary Spn6B carriage	Colonisation rates of Spn6B determined by classical culture and molecular methods
4. To determine if primary RSV-A **challenge or infection** alters the density of secondary Spn6B carriage	Density of Spn6B carriage by classical culture and molecular methods
5. To determine if primary RSV-A **challenge or infection** alters the duration of secondary Spn6B carriage	Quantification of Spn6B determined longitudinally by classical culture and RT-qPCR
6. To determine if primary Spn6B **challenge** alters the risk of secondary RSV-A infection	RSV-A attack rates determined by RT-qPCR
7. To determine if primary Spn6B **carriage** alters the risk of secondary RSV-A infection	RSV-A attack rates determined by RT-qPCR
8. To determine if primary Spn6B **challenge or carriage** alters the viral load of secondary RSV-A infection (AUC of density over time)	RSV-A viral load determined by RT-qPCR
9. To determine if primary Spn6B **challenge or carriage** alters the duration of secondary RSV-A infection	Duration of RSV-A detection and quantification determined by RT-qPCR
10. To determine if co-infection with RSV-A and Spn6B alter URTI and LRTI symptoms	Number of URTI and LRTI symptoms per participant after secondary challenge
11. Longitudinal assessment of viral and bacterial shedding from the nose in both arms of the study	Shedding of pathogens from the nose and throat will be assessed by coughing plate, hand swab after nose touching and facemasks assessments<br>RSV-A viral load and Spn6B density by RT-qPCR

12. To determine if primary Spn6B **carriage** increases the proportion of participants from whom live RSV-A can be isolated from their hands	As per objective 11
13. To determine if primary or secondary RSV-A **infection** increases the proportion of participants from whom live Spn6B can be isolated from their hands	As per objective 11
14. To determine if Spn6B **carriage** density correlates with bacterial shedding	As per objective 11
15. To determine if RSV-A nasal and throat viral load correlates with viral shedding	As per objective 11
16. To determine if the outpatient RSV-A challenge model is associated with any increased risk of infection in household contacts	Nasopharyngeal or nasal swabs of household contacts during two weeks following RSV-A challenge visit
**Exploratory objectives including:**	
17. To identify if primary Spn6B carriage alters immune responses to secondary RSV-A infection (innate and adaptive)	Assessment of immune responses including nasal cytokines and cell populations as well as RSV-specific antibodies and cellular immunity before and after each pathogen challenge.
18. To identify how primary RSV-A challenge or infection alters immune responses to secondary Spn6B carriage	Assessment of immune responses including nasal cytokines and cell populations as well as Spn6B-specific antibodies and cellular immunity before and after each pathogen challenge.
19. Nasal cells gene expression alterations and their visualization (spatial location) within nasal tissue	Longitudinal analysis of the spatial microenvironment and transcriptomics of nasal tissue samples
20. Characterise transcriptional changes in immune cells (via single cell and bulk RNA sequencing) in response to RSV-A and Spn6B challenge/infection/carriage.	RNAseq analysis to determine gene induction and regulation to identify gene signatures (cell specific where possible) that correlate with susceptibility to infection, as well as alterations in immune responses and symptoms during co-infection
21. Assess pneumococcal transcriptional alterations in response to RSV-A co-infection	Determination of gene induction and regulation to identify bacterial response to viral co-infection

Time points and sample types for outcome measures will be detailed in the study SAP.

7. Study Design

This is an open-label randomised controlled human co-infection study using previously established and safe models of pneumococcal and RSV infection with a pilot phase. This will be a multi-center study conducted in Oxford and Liverpool. Healthy volunteers will be challenged with either Spn6B or RSV-A at day 0 followed by a secondary reciprocal challenge (RSV-A or Spn6B) at day 7. The experimental human pneumococcal challenge model is established as an outpatient model (>2000 participants have completed the programme with no evidence of community transmission). The RSV-A model has also been established as an outpatient model in the Netherlands (Eudract: 2021-004025-77) with participants subject to self-isolation in the community setting (similar to that imposed during the recent COVID-19 pandemic). Volunteers will be trained in transmission prevention measures and will be screened for caring for or living with children under the age of 5years of age or other high-risk groups in the household.

7.1 Phase A

Participants will be randomised 1:1 to primary RSV-A challenge OR primary Spn6B challenge. Reciprocal challenge will occur seven days later. Participants will be subject to confinement within a dedicated clinical research facility for 10 days (reduced to 7 days if RSV-A negative result by point of care testing or nucleic acid amplification test (NAAT) methods on day 7) after either primary or secondary RSV-A inoculation. This is to facilitate enhanced monitoring by a resident clinically trained member of the research team.

The parameters for progression to Phase B are:

1. No experimental challenge related serious adverse events (SAEs)
2. Study team to confirm the self-isolation standard operating procedures (SOPs)
3. Review and approval to progress by TSC and DSMC

Patient and Public Involvement consultation will be sought to inform any necessary changes to participant-facing documents which are needed based on the results of Phase A.

7.2 Phase B

Participants will undergo primary challenge with Spn6B or RSV-A at day 0. Subsequently, participants will have reciprocal secondary challenge at day 7. Participants will self-isolate at home for up to 10 days after either primary or secondary RSV-A challenge, following SOPs validated within Phase A. If a volunteer has a negative result (measured with a NAAT) on day 7 they may terminate the self-quarantine. If the volunteer has a positive result on day 7, then they must remain in self-quarantine until day 10.

All household contacts of participants will be asked for consent to take part in twice weekly nasopharyngeal or nasal swabbing during the two-week period following RSV-A challenge, for which they will be offered remuneration. A household contact is defined as a person who shares a kitchen with the study participant.

If a PCR result from a household contact confirms the presence of RSV, the isolate will be fully sequenced to clarify whether the positive result is likely to have originated from the study participant. Swabs will be self-collected by the household contact, stored in the freezer and returned to the study

team by the participant at their next visit. If household contacts decline screening, this will not preclude participants from being enrolled.

Participants will be randomised in advance of their inoculation visit at day 0 and informed of their allocation to facilitate planning for individuals and research staff. Longitudinal testing for pathogen detection will occur at 2, 7 and 14 days after each challenge (samples 60 days after primary challenge for immunological assessments only, see table 2A). Participants will be asked to visit our outpatient facility (wearing a fluid resistant surgical mask and avoiding public transport) for sampling. In addition, participants will be trained to take samples (nasosorption, saliva and nasopharyngeal/nasal swab) whilst in isolation. Based on our power calculation, a total of up to 113 participants will be recruited to the study to allow for 90 participants to complete (20% expected drop out). A participant will be considered enrolled to the study at the point of first inoculation.

7.3 Phase C

Phase A will also determine if it is feasible to proceed to Phase B without pre-screening for pre-existent levels of IgG to RSV. If fewer than 2 volunteers are infected with the challenge virus in Phase A, then Phase C will be conducted instead of Phase B (data will be reviewed and decision made by TSC). Phase C will be identical to Phase B, but the study population will be pre-screened for RSV neutralizing antibody titres. Only participants in the lowest 10th percentile of RSV neutralising antibody titres will be included in the study. This pre-screening is expected to increase the virus attack rate by approximately 10% (personal communication, Christopher Chiu, Imperial College). The screening is not done in the first place due to costs, resources and patient burden. TSC may decide to revert back to Phase B at any point (following review and decision by TSC).

8. Participant identification

8.1 Study participants

Eligibility assessment will be completed in stages:

- Screening: study research staff highlights to participants the general screening criteria (including medical history) as part of the study information process before consent is taken.
- Eligibility is confirmed on the formal screening (V0), inoculation (V1) and follow-up visits (Table 2A). Final eligibility will be signed off by a medical doctor in the CRF prior to the primary inoculation.

8.2 Inclusion criteria

- Healthy adults aged 18-55 (inclusive, at the time of consent)
- Fluent spoken English – to ensure a comprehensive understanding of the research project
- Capacity to provide written informed consent in English
- Females of childbearing potential* with a negative urine pregnancy test at screening and willing to practice adequate contraceptive** measures as per UK Clinical Trial Facilitation Group (43) during the study.
- Willing to provide their household contacts with the Close Contact Screening Information Letter
- For Phase C, RSV neutralising antibody titre in the lowest 10th percentile of screened participants

*A woman is considered of childbearing potential, i.e. fertile, following menarche and until becoming post-menopausal unless permanently sterile. Permanent sterilisation methods include hysterectomy,

bilateral salpingectomy and bilateral oophorectomy. A post-menopausal state is defined as no menses for 12 months without an alternative medical cause. A high follicle-stimulating hormone (FSH) level in the post-menopausal range may be used to confirm a post-menopausal state in women not using hormonal contraception or hormonal replacement therapy. However, in the absence of 12 months of amenorrhoea, a single FSH measurement is insufficient.

**Acceptable effective forms of contraception for female volunteers include:

- Established use of oral, injected or implanted hormonal methods of contraception.
- Placement of an intrauterine device (IUD) or intrauterine system (IUS).
- Total abdominal hysterectomy.
- Bilateral tubal occlusion.
- Barrier methods of contraception (condom or occlusive cap with spermicide).
- Male sterilisation, if the vasectomised partner is the sole partner for the subject.
- Sexual abstinence defined as refraining from heterosexual intercourse during the entire period of risk associated with the study interventions The reliability of sexual abstinence needs to be evaluated in relation to the duration of the clinical trial and the preferred and usual lifestyle of the subject.

8.3 Exclusion criteria

Exclusion criteria will be self-reported and/or confirmed from GP questionnaire (GPQ) or medical summary if deemed necessary at research clinician discretion. Individuals may not participate in the study if they are/ have:

- Research participant:
  - Currently involved in another study unless observational or non-interventional. Exceptions may be applied at the discretion of the Chief Investigator to ensure no harm comes to the participants (e.g. excessive blood sampling or nasal sampling)
  - Participated in a previous Spn6B EHPC study ≤3 years or an Spn3 EHPC study ≤1 year before screening
- Unable to travel to outpatient clinic for visits during phase B/C after RSV challenge (for up to 10 days or 7 days if negative for RSV) without using public transport
- Unable to wear a fluid resistant surgical mask
- Vaccination**:**
  - No live vaccination within four weeks prior to enrolment (defined as time of first inoculation)
  - Any previous pneumococcal or RSV vaccination (including in a research study)
- Allergy:
  - Allergy to beta-lactam antibiotics (including penicillin and amoxicillin)
  - Allergy to Lidocaine local anaesthetic (for optional nasal biopsy participants only)
- Medical history leading to increased risk of severe infection or illness including but not limited to:
  - Asplenia or dysfunction of the spleen
  - Chronic respiratory disease (e.g. asthma [requiring medication (including salbutamol inhaler) within last 12 months], COPD, bronchiectasis and sleep apnoea)
  - Chronic heart disease (e.g. angina, ischaemic heart disease, chronic heart failure) – controlled and stable hypertension may be included
  - Chronic kidney disease (e.g. nephrotic syndrome, kidney transplant, requires dialysis)
  - Chronic liver disease (e.g. cirrhosis, biliary atresia, hepatitis)
  - Chronic neurological disease that limits mobility, bulbar or respiratory function (including stroke, Parkinson’s disease, dementia and multiple sclerosis)
  - Diabetes mellitus (including diet controlled)

    - Receipt of immunosuppressive therapy such as anti-cancer chemotherapy or radiation therapy within the preceding 12 months or long-term systemic corticosteroid, Roaccutane, or disease modifying anti-rheumatoid drugs therapy (for more than 7 consecutive days within the 3 months prior to enrolment).
    - Individuals with cochlear ear implants
    - Individuals with major cerebrospinal fluid leaks (e.g. following traumatic, major skull surgery, or requiring CSF shunts)
    - Subjects with known or suspected immune deficiency (e.g. known IgA deficiency, immotile cilia syndrome, or Kartagener's syndrome)
    - Autoimmune disease
    - History of frequent nose bleeds
    - Bleeding disorders
    - History of significant unexplained bleeding after a surgical or dental procedure (for optional nasal biopsy participants only)
- Current medical issues
    - Acute upper respiratory tract infection in the four weeks preceding recruitment (as per definition in Annex 1)
    - Any uncontrolled medical or surgical condition (e.g. mental health conditions, epilepsy, narcolepsy or chronic pain) at the discretion of the study doctor
- Any major pneumococcal illness or pneumonia requiring hospitalisation in the last 10 years
- Medication
    - Any medication that may affect the immune system in the last 3 months (e.g. systemic steroids [IM/IV], Roaccutane, disease modifying anti-rheumatoid drugs, chemotherapeutic agents)
    - Long-term antibiotic use
    - Recipient of monoclonal antibodies for any indication within one year of period at screening.
    - Recipient of blood transfusion products within the last year
    - Any medication that may affect the coagulation system in the last 3 months (excluding low dose (75mg) aspirin).
    - Use of any medication or other product (prescription or over-the-counter) for symptoms of rhinitis or nasal congestion within the last 1 month
- Maternal
    - Female participants who are pregnant
    - Female participants who are lactating
    - Female participants who intend to become pregnant during the study
    - Female participants unable to take contraception measures (43) during the study (from consent to final study visit at day 60)
- Direct caring role or share living accommodation with individuals at higher-risk from infection
    - Children under the age of 5 years of age
    - Adults > 65 years old
    - Adults with chronic ill health or immunosuppression
    - Adults classified as clinically extremely vulnerable by the NHS (44)
- Health-care worker
- Smoking
    - Current or ex-smoker (regular cigarettes/cigars/e-cigarette/vaping/smoking of recreational drugs) in the last 6 months
    - Previous significant smoking history (more than 5 cigarettes per day for 20 years or the equivalent [i.e. >5 pack years]).
- Current alcohol and recreational drug use
    - Regularly drinks ≥3units/day (male) or ≥2units/day (female)

- Regularly uses recreational drugs
    - Participants may be excluded at the discretion of the research clinician
- Significant mental health disorders
    - Uncontrolled condition or previous admission to a psychiatric unit (at the discretion of the research clinician) which would impair the participants ability to safely participate in the study
    - Moderate or severe depression or anxiety as classified by the Hospital Anxiety and Depression Score at screening or challenge that is deemed clinically significant by the study doctors
- Overseas travel planned during 21 day period following first inoculation
- Participants whose FBC results do not meet the required criteria on screening bloods

Full blood count exclusion parameters	Hb <90 g/L, total WCC <1.5 x10^9/l<br>total WCC >12 x10^9/l platelets <75 x10^9/l

- Any other issue which, in the opinion of the study staff, may:
    - Put the participant or their contacts at risk because of participation in the study
    - Adversely affect the interpretation of the study results, or
    - Impair the participant's ability to participate in the study
- Study site staff or a partner of study site staff.

8.4 Temporary exclusion criteria

The following are temporary exclusion criteria to primary inoculation:

- Current acute infective illness – delay inoculation by 14 days
- Recent /current URTI – delay inoculation by 4 weeks after last day of illness.
- Asymptomatic positive COVID-19 OR RSV-A swab (taken on day of planned challenge) – delay inoculation by 21 days.
- Antimicrobial (including antiviral) use – delay inoculation by 28 days from last date of antimicrobial therapy
- Nasal carriage
    - Participants who have natural pneumococcal carriage identified at visit 0 will be excluded (see table 2A) – delay until carriage clearance.

The following are temporary exclusion criteria to nasal biopsy:

- Antibiotic use (during the study)-delay nasal biopsy for at least 1 week from last date of therapy
- Dental infections- delay nasal biopsy for at least 2 weeks after last day of illness

Potential participants who are temporarily excluded at screening or prior to first inoculation may be re-screened at a later date to assess inclusion into the study. There is no time limit to re-screen a potential participant, however, participants would be re-consented if the time since initial written informed consent is greater than 3 months. Participants who are delayed may be re-screened at the discretion of the investigator, including screening samples prior to formal enrolment (defined as time of first inoculation). Participants who meet any of the temporary exclusion criteria to primary inoculation may have their inoculation delayed until resolution of the temporary exclusion.

For clarity, a participant who has a positive RSV-A swab at day 7 following primary RSV inoculation will proceed to second inoculation with Spn unless they meet individual stopping rule criteria (see section 12.8) or at investigator discretion.

If a participant unexpectedly requires a vaccination during the study period, they will remain in the study and will be considered as a part of an intention-to-treat subgroup for the purposes of the analysis.

9. Study procedures

9.1 Recruitment

Healthy adults between 18 and 55 years (inclusive) will be recruited for the study. The study will be split into two phases: a Phase A (up to n=10) to determine safety and feasibility of the co-infection model and a Phase B/C randomised controlled trial phase (up to n=103). See power calculation in section 14.11 for further details. Participants who drop out before completion of both challenge visits and at least day 2 post pneumococcal challenge will be included in the safety and secondary endpoint analysis, but will be excluded from primary endpoint.

All potential participants may be contacted by methods including but not limited to email, telephone, posters, leaflets, websites, advertisements in newspaper, radio and on social media, public engagement events and/or mail using a REC approved invitation letter or other advertising material using wording from REC approved study documents in the first instance to invite them to participate in the study.

Where mail-outs are used, participants may be identified via the electoral open register, or through National Health Service databases using data extracts. For the NHS databases initial contact to potential participants will not be made by the study team. Instead, study invitation material will be sent out on behalf of the study team by an external company, CFH Docmail Ltd (or equivalent company), in order to preserve the confidentiality of potential participants. CFH Docmail Ltd (or equivalent company) is accredited as having exceeded standards under the NHS Digital Data Security and Protection Toolkit (ODS ID – 8HN70).

For mail-outs via the electoral register, the study team will obtain access to the names and addresses of individuals who are on the open electoral register (only contains the names of registered voters who have not opted out). In this instance, the study team will upload the mailing list to the CFH Docmail system (or equivalent company), and the study invitation pack will be sent out by CFH Docmail (or equivalent company).

The details of other recruitment methods that may be used are outlined below:

1. Email/Poster campaign: We will contact representatives of local tertiary education establishments and local employers and ask them to circulate posters and link to study website by email or hard copy.

2. Oxford Vaccine Centre (OVC) database for healthy volunteers/other databases: The study may be advertised on the electronic newsletter sent out to those potential participants signed up to the Oxford Vaccine Centre's Healthy Volunteers Database. Additionally, by email distribution to potential participants registered on the OVC Healthy Volunteers Database or similar databases (where members of the public have given their consent to be contacted when studies open for recruitment and understand that this is not a commitment to

participate), or to a group or list only with the express agreement of the network administrator or with equivalent authorisation.

3. Media advertising: Local media, newspaper and website advertisement placed in locations relevant for the target age group with brief details of the study and contact details for further information.

4. Website advertising: Description of the study and copy of information booklet on study team websites and other appropriate platforms for vaccine trial advertising.

5. Social media: Advertisements placed on trial site media accounts or targeted social media platform advertisements including, but not restricted to, Twitter, Facebook and Instagram

6. Exhibitions: Advertising material and/or persons providing information relating to the study will exhibit using stalls or stands at exhibitions and/or fairs, such as University Fresher's Fairs.

7. Volunteers may also be recruited using direct SMS/text message, or emails to potential participants identified by GPs from their databases (PIC agreements to be set up with the GP surgeries as required).

8. Royal Mail Leaflet: Royal Mail door-to-door service with delivery of invitation letters in site envelopes to every household within certain postcode areas.

Research ambassadors and research staff will attend public engagement events as stated above to promote our research and engage the public.

Potential participants who are interested in study participation will be able to contact the sites by telephone, email, online or a reply slip.

9.2 Screening and eligibility assessment

Once an expression of interest has been received, an information sheet will be downloaded from the study website by the potential participant, and/or sent to them via mail or email. Following this information, if participants are willing to proceed, they will be asked to complete an initial online and/or telephone questionnaire before they are invited for a screening and consent visit, where their eligibility will be assessed by member of the clinical research team at the study site. Procedures will take place as listed in 9.7.

Permission to access the volunteer medical records either via the electronic patient record (EPR) or GP will be sought (if possible) prior to the screening visit. Participants will be asked to sign a secure electronic document (hosted by the REDCap database). Alternatively, written permission to access medical records will be sought at the volunteers screening and consent visit.

9.3 Informed Consent

Consent will be sought following relevant local SOP's. Potential participants will be invited to discuss the study at visit 0.

Written and verbal versions of the Participant Information sheet and Informed Consent will be presented to the participants detailing no less than: the exact nature of the study; what it will involve

for the participant; the implications and constraints of the protocol; the known side effects and any risks involved in taking part. It will be clearly stated that the participant is free to withdraw from the study at any time for any reason without prejudice to future care, without affecting their legal rights, and with no obligation to give the reason for withdrawal.

Sites may invite groups of participants for a study presentation and discussion (either remotely or in person) prior to visit 0.

The participant will be allowed as much time as needed to consider the information and will have the opportunity to question the Investigator (in private), GP or other independent parties to decide whether they would like to participate in the study.

Participants will be asked to complete a consent quiz as part of the informed consent process to ensure they have properly understood the study and provide an opportunity to review any areas that the participant may require further information before consent is taken.

Written Informed Consent will then be obtained by means of participant-dated signature and dated signature of the person obtaining the Informed Consent. The person who obtained the consent must be suitably qualified and experienced and have been delegated to do so by the Chief/Principal Investigator. A copy of the signed Informed Consent will be given to the participant. The original signed form will be retained at the study site.

If individuals agree to participate in the study and the study team are satisfied that they meet the clinical eligibility criteria, they will be invited to provide written informed consent and further clinical appointment will be made for formal screening to take place. The consent form includes an option for participants to allow the DNA/RNA from these blood samples to be studied.

The PIS and presentation (presentation will be based on PIS) will inform the volunteers that they may withdraw from the study at any time and that this will not affect the care they receive within the NHS.

A GPQ / summary will be requested if clinically indicated during the eligibility assessment at the consent visit. This will be reviewed by the study doctor prior to the primary inoculation appointment A continuous consent approach will be used throughout the study as participants will be asked at each visit if they are willing to continue.

As part of recommended practice (MRC tissue and biological samples for use in research (45)) participants will be asked to consent at screening to gift their samples for use in future studies and they may be shared with international collaborators. All samples will be de-identified. Any samples remaining at the end of the study will be transferred to the OVC biobank [Biobank REC number is 21/SC/0161] providing appropriate consent has been obtained.

Optional study procedures:

A subset of participants will also be offered an optional nasal biopsy based on availability. We will aim to collect up to 40 (divided among study groups, refer to table 3 for study groups). Consent for this will be sought at screening or after enrolment. Nasal biopsy samples will be used to characterise the nasal cells gene alterations and their visualisation (spatial location) within the nasal tissue. This information will allow a better understanding of the mucosal immune mechanisms that relate to susceptibility/protection to RSV infection and Spn6B colonisation.

9.4 Consent of household close contacts for screening

At V0, the participant will be provided with a letter to take home to close household contacts to invite them for screening. Household contacts will be offered to be screened for RSV infection (using a NAAT), which will involve obtaining twice weekly nasopharyngeal or nasal swabs at days 2, 7, 9 and

14 after the participant has received intranasal challenge with RSV. A step-by-step sample self-collection guidance will be provided. Samples can be stored at home in the freezer until the participant brings the sample to their next visit. Contacts will be notified of positive results. Household close contacts will also be asked for consent to store their identifiable data for the duration of the study as well as the storage of any samples which they provide. Failure to provide consent to storage of personal information/sample storage will not prevent participants from being offered screening.

9.5 Randomisation

Participants will be randomised after clinical screening and screening samples have been taken at visit 0.

The study will comprise of two phases: a randomised pilot phase (up to n=10 participants) to determine the safety and feasibility of the co-infection model and a secondary randomised controlled phase (up to n=103 participants) to determine if nasal RSV-A challenge significantly increases experimental Spn6B carriage rates. For Phase A, up to 10 participants will be randomised in 1:1 ratio to RSV-A or Spn6B at day 0 followed by reciprocal challenge seven days later. This is to optimize the isolation following RSV-A challenge and to evaluate the safety of the co-infection model.

A computer generated randomisation list will be prepared by the study statistician and will be stored on a secure electronic portal accessible by the statistics and IT team. Trials participants will be randomised 1:1 to the two study arms. Random block sizes will be used. Since this is an open-label study, trial participants and the clinical staff involved in the study delivery will be unblinded. Randomisation will occur prior to primary challenge and the allocation will be communicated to study participants at least 48 hours prior to challenge so that they can prepare for their 7-10 days of self-isolation after RSV-A challenge. The lab team conducting immunological assays will be blinded to participant allocation when processing samples for outcome measure assessment.

In order to maintain a balance in phase A (5 participants in each arm), participants who are randomised but unable to be inoculated according to the protocol will be replaced. These participants might be enrolled on Cohort B if they are eligible. There will be no replacement in phase B. For phase A, if the dropouts happen in both arms, additional participants will be randomised to meet the recruitment target. On the other hand, if the dropouts occur in only one study arm, the participants will be replaced instead of randomised.

For phase B, randomisation will be stratified based on participants' gender (male/female) to account for differences in the likelihood of Spn colonisation between genders.

Participants will be randomised to one of two groups:

1. Arm P-RSV: *Streptococcus pneumoniae* serotype 6B inoculation at day 0 followed by RSV-A Memphis 37 strain inoculation at day 7.
2. Arm RSV-P: RSV-A Memphis 37 strain inoculation at day 0 followed by *Streptococcus pneumoniae* serotype 6B inoculation at day 7.

9.6 Blinding

Participants and clinical research teams will not be blinded in this study. Laboratory staff will be blinded to study allocation during conduct of research assays. Blinding plan will be described in the Randomisation and blinding plan.

9.7 Study interventions, comparators and study procedures

Study Visit<br><br>Day post inoculation	V0** 	V1<br><br>0	V2<br><br>2	V3<br><br>6	V4<br><br>7	V5<br><br>9	V6<br><br>14	V7<br><br>21	V8<br><br>60	V9 	Biopsy Visit V10<br>(Optional)	V11<br><br>1day post V10	V12<br><br>21days post V11
Visit window (days post inoculation)	Up to - 60		1 to 3	5 to 7	6 to 8	8 to 10	13 to 15	19 to 23	53 to 67	Up to - 7[%]	22 to 142	1-3post biopsy	14-28post biopsy
Consent (Written)*	x										x[&]		
Consent (Verbal)		x	x	x	x	x	x	x	x	x	x	x	x
Study information	x												
Study screening	x									X			
TOPS initial check and registration www.tops.org.uk***	x												
Clinical Exam	x												
ECG[γ]	x												
Randomisation[#]	x												
Vital Signs	x	x	x	x	x	x	x	x	x		x[$]		
Screen for AEs		x	x	x	x	x	x	x	x				
Screen for SAEs		x	x	x	x	x	x	x	x			x	x
Inoculation[‡]		x			x								
Pregnancy test	x	x			x								

**FBC, U&E, LFT, CRP<br>+/- coagulation screen**	x				x			x					
**HIV, HepB/C test**	x												
**PBMCs**	x[1]			x			x	x	x				
**Serum**	x[1]			x			x	x	x				
**Blood collected for RNA**		x	x		x	x							
**Nasosorption**		x	x	x	x	x	x	x					
**Nasal swab**	x	x	x	x	x	x	x	x					
**Nasal wash**	x		x	x		x	x	x	x				
**Nasal cells^**	x		x	x		x	x	x	x				
**Throat swab^^**	x	x	x	x	x	x	x	x	x				
**Saliva**	x		x	x		x	x	x	x				
**Cough sample[α]**			x	x		x	x						
**Emission sample[β]**			x	x		x	x						
**Nose to hand swab[£]**			x	x		x	x						
**Mood assessment[z]**	x				x								
**Nasal Biopsy[π]**											x		

Table 2A: Study procedures and sampling schedule at sites. V0 may occur for up to 60 days before visit V1. Samples may be repeated at investigator discretion for safety or to check eligibility. There will be a flexibility ± one or two working days at all other visits. Participants will be asked to keep a symptom diary and conduct self-testing after inoculation with RSV-A in addition to scheduled visits (detailed below). FBC: full blood count; PBMCs: peripheral blood mononuclear cells; RNA: ribonucleic

acid. *Written consent may occur on the same day as the information visit or one week later if the potential participant requires more time to consider or if the information visit has been conducted remotely via video link. **Consent discussion and obtaining formal written consent and screening visit procedures may occur over two separate visits. *** TOPS: The Over-volunteering Prevention System- if required per local policy. [1]Samples can be collected at V0 or V1.

[#]Randomisation will be conducted after participants are confirmed eligible for the trial (including results of nasal wash to confirm absence of natural pneumococcal carriage) with allocation communicated to participants in advance of V1 with at least 48 hours notice. Participants will be considered enrolled to the trial at the point of first inoculation. ‡ Participants will be randomised to primary inoculation with RSV-A or Spn6B at day 0 followed by reciprocal challenge at day 7. Swabs at days 0 and 7 (days of inoculation) will be tested for RSV by molecular testing and primary RSV inoculation will proceed only if negative result is confirmed. For clarity, secondary inoculation with Spn may be performed in RSV positive individuals as per Section 8.4 . [μ]To be collected 7 days after RSV inoculation to determine length of isolation period. ^Nasal cells to be taken AFTER nasal wash. ^^Throat swab to be taken prior to nasal swab. α: A cough sample will be collected by asking the participant to cough into a microbiological plate according to established SOPs. β: in a subset an emission sample will be collected by asking the participant to breathe and talk into a face mask with an internal microbiological/virological strip to collect the sample per established SOPs (46). £: Participants will be asked to rub their noses with the back of their hands and their hands will be then swabbed for bacterial and viral detection in a subset of participants. [γ]: ECG will be performed as part of inpatient confinement procedures according to the clinical research facility requirements, and if clinically indicated. [z]: participants will be asked to complete a mood assessment questionnaire (Hospital Anxiety and Depression Score) at screening, prior to the second challenge administration and may be performed at additional timepoints at clinician discretion. π: Optional Nasal biopsy collected from inferior turbinate and/or postnasal NALT tissue from a subset of volunteers. [%]Temporary exclusion criteria will need to be assessed prior to nasal biopsy, this can either be done at a separate phone call or at V7 or V8, if these visits fall within the window period to assess temporary exclusion criteria. [&]Vital signs may be performed at the discretion of the ENT specialists. Safety follow-up: Participants after nasal biopsy procedure will be observed for 30 minutes before leaving the facility. A phone safety review will be conducted by the study team at 1 day and 3 weeks after the nasal biopsy. Participants undertaking nasal biopsy will undertake a separate additional written consent for the procedure which will be performed or re-confirmed on the day of the biopsy by the operator.

Blood Volumes

The following blood volumes are the maximal expected to be drawn at each visit:

V0 – 74 ml (FBC, U&E, LFT, HIV and HepB/C serology, PBMCs and serum) +/- coagulation screen

V1 and 2 – 2.5ml (Paxgene)

V3 and V6 and V8 – 50 ml (PBMCs and serum)

V7- 65 ml (PBMCs, serum + FBC, U&Es, LFT, CRP) +/-coagulation screen

V4 – 20.5 ml (FBC, U&E, LFT and Paxgene)

V5 – 2.5ml (Paxgene)

Unless otherwise specified, up to 40mls of blood for PBMCs and 10mls of blood for serum will be drawn. The exact volume of safety blood samples sent to clinical laboratories will depend on local sampling bottles. Cumulative blood volume drawn over the course of the study = 317 ml

Coagulation screen will be done either on V0 or V7

For their safety, inpatient participants in Phase A will also undergo daily review including the following schedule of events (table 2B):

Procedure	Days post RSV inoculation
Vital signs	Days 1-7/10
AE review	Days 1-7/10

Table 2B: Inpatients only: additional procedures for participants in Phase A

Study Visit	V1				Daily until V4	V4				Daily until V7
Hours post inoculation	2	6	12	24*		2	6	12	24*	
Nasosorption	x	x	x	x		x	x	x	x	
Saliva	x	x	x	x		x	x	x	x	
Nasal swab	x	x	x	x		x	x	x	x	
Symptom diary				x					x	

Table 2C: Participant self-sampling schedule. Participants will be trained to take a nasosorption sample and a nasal swab sample from opposite nostrils after infection challenge. This sampling strategy is designed to quantify inflammatory and infection dynamics after infections challenge. * After the first 24 hours, participants will be asked to take a daily nasosorption, saliva and nasopharyngeal/nasal swab samples at approximately the same time ± 2 hours until V4 and V7 respectively. We will ask participants to document a symptoms diary in addition to these samples on a daily basis until V7. On days which coincide with a clinical visit, participants can bring the sample to the visit. During phase A, clinical staff will bring the self-collected samples to the processing laboratory on day of collection.

9.8 Preparation of Spn6B challenge agent

The dilution of the inoculum will be prepared as per local SOPs in a room dedicated for challenge agent preparation. A mid-log broth culture of pneumococcus (Spn6B) will be frozen at -80°C in aliquots of glycerol-enriched media. Frozen aliquots will be thawed and checked for bacterial number (colony

forming units [CFU] per ml), and purity. These checks will first be carried out in research laboratories and then identification, purity by whole genome sequencing and penicillin sensitivity will be confirmed in a reference laboratory (UKHSE). On experimental inoculation days, aliquots will be thawed, washed twice, and re-suspended in 0.9% normal saline at the correct density. Once inoculum is prepared it will be administered within 30min to avoid degradation.

We will aim for a dose of 80,000 CFU/100µl per naris of the Spn6B inoculum (inoculum dose determined from previous challenge studies). We will allow a variation of half or double of this dose and have previously demonstrated in our dose-ranging studies that this range is safe and leads to similar colonisation outcome (rates and density recovered from the nasopharynx) (16).

9.9 Preparation of RSV challenge agent

The RSV stock intended for use in this study is designated RSV-A Memphis 37 and is supplied by Imperial College London. The virus was isolated from a child with severe RSV infection in the USA in 2007 and manufactured according to current Good Manufacturing Practice (cGMP) in human Vero cells. Batch was tested and is free of adventitial agents and human pathogens.

RSV-A Memphis 37 inoculum was developed specifically for human challenge. It was isolated from a natural infection occurring in a neonate in the USA by plaque purification in human Vero cells (5 passages only) and expanded under fully GMP-compliant conditions. It was then tested according to appropriate International Conference on Harmonisation (ICH) of Good Clinical Practice (GCP) and the United States Food and Drug Administration (FDA) guidance documents for the production of a human clinical vaccine product. This virus has been used to infect over 300 healthy adults in studies without reported serious adverse events. Infectivity in humans has been demonstrated for doses of 3-5 log PFU. On the day of inoculation, viral doses will be defrosted on ice in a dedicated area by trained research staff wearing appropriate PPE (see below for details).

Diluted Inoculum

RSV-A Memphis 37 is presented in 1mL vials diluted in 25% sucrose and DMEM. On the day of challenge the RSV challenge agent will be diluted to the required dose following established SOP (collaboration with Prof Chis Chiu, Imperial College London). Once inocula are prepared they must be kept on ice and administered to participants within 1 hour to avoid degradation.

Transport of inocula to clinics

Labelled inocula (pneumococcus and RSV) are stored at dedicated -80°C in the designated laboratory of the study sites. Prepared inocula are transported to the site clinical facilities in a clearly labelled vial and transportation bag. Inocula will be placed at room temperature for pneumococcus and on ice within a closed polystyrene box for RSV.

Inocula will not be opened before reaching the designated room of the research clinic in which volunteers are to be inoculated.

Supply and accountability

Accurate records of receipt and condition of all Challenge stock agents will be available for verification by the Study Monitor. Trained site staff will be responsible for adequate and accurate accounting of

all Challenge Agents inoculum. Any deviation from the protocol-dispensing regimen will be fully documented.

9.10 Inoculation

A viral swab for RSV and COVID-19 will be done with a point of care test to confirm participant negative status before inoculation.

Inoculation of bacteria

The inoculum will be administered according to local SOPs. Briefly, the participant will be seated in a semi-recumbent position. Using a micropipette, 0.1ml of pneumococcus-containing-fluid will be instilled into each nostril. This will be done slowly with sufficient interval between each inoculation to ensure maximum contact time between the nasal and pharyngeal mucosa. After inoculation, the participant will remain in this position for up to 15 minutes. Participants will be asked not to wash or blow their noses for at least one hour.

Inoculation of virus

The inoculum will be administered according to local SOPs. Briefly, the participant will be seated in a semi-recumbent position. Using a micropipette, RSV-containing-fluid will be instilled into each nostril. This will be done slowly with sufficient interval between each inoculation to ensure maximum contact time between the nasal and pharyngeal mucosa. Subjects will be asked not to swallow during the procedure to ensure maximal pharyngeal contact. Following inoculation, advice regarding hand hygiene will be given and subjects will be provided with alcohol hand gel to reduce spread of virus in the environment.

Research staff performing inoculation will wear full personal protective equipment including gloves, gown, safety goggles and fluid resistant surgical masks. RSV-A inoculation will occur in a separate clinical room to pneumococcal inoculation with appropriate cleaning to disinfect the area between inoculation episodes.

Post inoculation

After the first inoculation participants will be given a safety pack containing:

- Thermometer
- Safety information leaflet (including how to take temperature and symptoms of pneumococcal infection)
- Medical alert card with study team contact details
- Amoxicillin 500mg TDS 3-day supply (including antibiotic patient information sheet). Participants will be asked to return any remaining antibiotic doses to reconcile and discard.

Written and verbal instructions are given to the participant describing potential mild, moderate and severe symptoms and the instances when antibiotics can be taken. These include:

- At an investigator's discretion at any point
- At the end of the study (after visit 7), if they have been positive for Spn6B, at any timepoint following inoculation, without having had two consecutive negative NWs before the last visit
- If unwell and/ or symptomatic and instructed to take by the research team, or
- If unwell and unable to contact the research team

Participants will be instructed to monitor the development of any symptoms at home (or in confinement) and complete diaries. Home monitoring of symptoms will include a clear flow chart of the necessary intervention should any symptoms develop (see participant safety information leaflet). A mood assessment questionnaire (Hospital Anxiety and Depression Score or HADS) will be completed by participants at screening and prior to administration of the second challenge. A member of the research team will review diaries and assessments daily in the first week following inoculation and attempt to contact the participants should they not make contact or fill in the diary by the specified time. If no contact is possible, then a prior defined 'secondary contact', from whom written consent has been obtained, will be telephoned. Participants will have access to a 24/7 on-call telephone number until the end of the study. Patients reporting symptoms potentially consistent with pneumococcal disease (for example, ear pain, sore throat, cough and fever) or moderate RSV disease will be seen in person for medical assessment and will begin the course of amoxicillin if the research staff feel symptoms could possibly be due to pneumococcal or another potential bacterial infection.

To diminish the risk of local transmission we will educate personnel and participants about the characteristics, transmission, and risk of RSV and pneumococcal infection before starting the study, frequent handwashing and use of surgical facemasks will be applied.

In the outpatient setting, participants will be required to keep all transmission preventing measures, including use of face masks, frequent handwashing and keeping 1.5m distance from any household members. We will consent household members to collect twice weekly pseudonymised (as used in all data protection legislation and regulation) nasopharyngeal or nasal swabs that will be stored in their home freezer and brought back to the clinic by the study participant at the study visits.

Monitoring of colonisation

Colonisation will be defined by the result of nasal washes taken at 2, 7 and 14-days after each inoculation on Day 0 and Day 7 as per visit schedule (Table 2A). Therefore, samples will be taken at Days 2, 7, 9, 14 and 21 after primary challenge. Additional samples will be taken at Day 60 (53 days after D7 secondary challenge), particularly important to measure serological responses as these may sometimes be delayed. Self-isolating participants will travel to our outpatient facility for their samples wearing a fluid resistant surgical mask and avoiding public transport according to SOPs we will develop during the pilot phase of the study.

Confirmation of pneumococcal colonisation

Nasal washes will be plated onto culture media, incubated overnight and colonies will be confirmed as *S. pneumoniae* using classical microbiological techniques. If and when microbiological culture confirms *S. pneumoniae* experimental colonisation in the laboratory, the clinical team will be informed to ensure participants are counselled appropriately. A participant is considered positive for colonisation if they test positive for pneumococcus at any nasal wash sample by either classical microbiology or molecular methods. If study participants persistently carry pneumococcus at their study visit 7, they will be instructed to take oral Amoxicillin 500mg three times daily for 3 days to clear the colonisation.

DNA will be extracted from nasal wash (NW) samples using our well-defined protocols (47). *S. pneumoniae* serotype 6B detection will be done by multiplex qPCR for *lytA* and *6BcpsA*, respectively. This technique will enable us to detect individuals who are potential carriers with very low bacterial density. This multiplex qPCR is well established and validated in our laboratories.

Confirmation of viral colonisation

Viral multiplex qPCR for detection and quantification will be performed on genetic material of stored swab and/or nasal wash to detect RSV-A and all common respiratory viruses (including SARS-CoV2). RSV might also be detected by ID NOW^{TM}. Quantitative cultures in HEp-2 cell plaque assays might also be performed in 12-well plates with triplicate 10-fold dilutions of nasal wash as previously described (42).

Definition of RSV infection: A participant will be considered RSV infected if they have positive samples (e.g. nasosorption, nasal swab, nasal wash) at any two consecutive swabbing timepoints after RSV-A experimental challenge detected in the same sample by qPCR.

Antibody measurement

As per our previous published work we will measure virus specific and pneumococcal serotype-specific antibody responses both systemically and at the respiratory mucosa. Response measurements will include viral and bacterial neutralisation assays *in vitro*. Antibody titres and function will be compared between participants. Participants will be divided into groups (table 3) depending on the order of challenge (primary RSV or primary Spn), Spn carriage and RSV infection confirmation. We will measure changes in responses post each infection to baseline. This will allow us to evaluate the effect of co-infection and order of pathogen exposure to humoral responses against RSV and Spn.

	**Positive for RSV infection**	**Positive for Spn carriage**
RSV+/ Spn+ (primary RSV)	+	+
Spn+/RSV+ (primary Spn)	+	+
RSV+/ Spn- (primary RSV)	+	-
Spn-/ RSV+ (primary Spn)	+	-
RSV-/ Spn+ (primary RSV)	-	+
Spn+/RSV- (primary Spn)	-	+
RSV-/Spn- (primary RSV)	-	-
Spn-/RSV- (primary Spn)	-	-

Table 3: Participant's group allocation based in order of the challenge, Spn carriage status and RSV infection.

Cellular responses

We will also assess cellular responses to RSV and Spn systemically (from PBMCs) and at the respiratory mucosa (nasal scrapes and biopsies). Viral and pneumococcal specific T cell recall responses (both CD4+ and CD8+) will be quantified post stimulation with viral or pneumococcal antigens, respectively, in all the study groups above. We will also measure levels of memory B cells to RSV and Spn6B in each study group. We will measure changes in the responses post each infection to baseline. Immune responses will be compared amongst the groups above. We will also evaluate changes in the innate immune cell dynamics, activation and functionality in a kinetic fashion in the nasal mucosa and correlate those findings with susceptibility to infection or ability to clear infection.

Genetic responses

As per our previously published work, we will use mRNA sequencing to perform in depth investigation of transcriptome changes in both peripheral blood and respiratory mucosal cells as a response to viral

and bacterial infection. Such gene signatures will be paired with immunophenotyping data and compared amongst the 4 study groups. This type of analyses will enable us to identify correlates of protection or susceptibility to RSV and pneumococcal infection and mechanisms of host-pathogen interaction or viral and bacterial interaction and how this affects the downstream immune responses. We will also perform transcriptomic analysis of recovered Spn from RSV- and RSV+ participants to determine how bacterial gene expression is altered by co-infection with RSV-A.

9.11 Baseline assessments

After eligibility and subsequent randomisation, the following procedures will be required:

Revalidation of consent
Check 24-hour contact details
Interim medical history including any significant events
Check temporary exclusion criteria to challenge
Vital signs and clinical examination
Urine pregnancy test for female participants
Sample collection as per Table 2A

All laboratory results will be reviewed and collated by the study team who will record these in the CRF. If a test result is deemed clinically significant, it may be repeated, to ensure it is not a single occurrence. If a test remains clinically significant, the participant will be informed. Depending on the nature of the result the participant may be asked to see their GP and be given the relevant information from any test results from the trial. Alternatively, if consent provided, the participant's GP may be contacted to discuss a particular result or finding. Decisions to exclude potential participants from enrolling in the trial or to withdraw a participant from the trial will be at the discretion of the Principal Investigator and Co-Investigators.

Participants may be re-screened at a later date to determine whether they meet eligibility criteria (e.g. if they have an acute infection on the day of screening that has subsequently resolved). They will be reimbursed for the additional study visit.

9.12 Inpatient observation – phase A

Participants in phase A of the study will undergo a 7-10 residential isolation stay following RSV inoculation. During this stay they will be reviewed daily including having their vital signs taken and a clinical examination if indicated. They will adhere to the same self-sampling schedule as if they were in the community and undergo the same schedule of study visits as listed in Table 2A. The length of their inpatient stay will be determined by an RSV nucleic acid amplification test on Day 7 post inoculation. If this is negative, they will be permitted end their isolation period and return home.

9.13 Subsequent visits

The following samples will be obtained during the study, detailed in Table 2A.

1. **Urine** – Participants will be asked to collect up to 20ml of urine for pregnancy testing (female)
2. **Throat swabs** will be obtained for detection of viral and bacterial pathogens by microbiological and molecular techniques. Swabs may also be taken at unscheduled visits, if participants develop

any respiratory symptoms outside the usual study schedule. The participant's tongue will be depressed using a tongue depressor exposing the palatopharyngeal arch. The sample is taken by making five small circular motions of the palatopharyngeal arch in contact with the mucosa whilst avoiding the participant's tongue. Performing throat swabs prior to nasal washes will ensure that the oropharynx is not inadvertently contaminated with nasal pathogens prior to throat swab sampling. Normally, up to 2 swabs may be taken at each time point, with additional swabs as required if the participant is symptomatic.

3. **Nasosorption** will be obtained before the nasal wash. Sample strips collect concentrated nasal lining fluid to measure inflammatory responses induced by an infection. Blotting paper will be held inside the nostril for 1- 3 minutes until soaked. These will then be removed and placed in a microcentrifuge tube for storage.
4. **Nasopharyngeal or nasal swabs and saliva** will be obtained for detection of viral (including SARS-CoV2) and bacterial pathogens. Regular nasopharyngeal or nasal samples will be taken (self-samples and by study staff to monitor RSV-A viral infection and Spn6B carriage dynamics). If the participant is symptomatic, additional swabs may be taken at study and unscheduled visits. Participants will provide saliva samples by spitting into a container.
5. **Nasal wash** will be performed using our established SOP to collect nasal flora/ pathogen specimens and soluble biomarkers (48). Briefly, 5ml of saline is instilled and held for a few seconds in the nares before being allowed to drip into a sterile pot; this is usually repeated up to 20ml in total. In the event of nasal wash loss (for example, if the participant coughs, sneezes, or swallows) the procedure may be repeated to obtain an adequate specimen (≥10ml return).
6. **Nasal cells** will be collected after nasal wash using flocked swabs and/or a nanosampling method in which cells are obtained through minimally-invasive superficial nasal scrape biopsies (rhinoprobe). Participants can be biopsied multiple times with no significant side effects (31, 32). Up to 4 samples will be obtained at each nasal sampling visit. If no cells are visible on the rhinoprobe following sampling, the sample will be repeated.
7. **Blood sampling** will be performed by trained, experienced staff. Up to 74ml of blood will be collected at a single visit to measure full blood count (for safety), and laboratory measures including, but not limited to serum immunoglobulins, PBMC populations, and host RNA expression.
8. **Cough sample:** to evaluate the transmission of bacteria from coughing, participants will be asked to cough on a culture plate.
9. **Emission sample:** participants wear the facemask so it covers both the nose and mouth for a duration of 15-60 minutes. There are no restrictions on talking or coughing while wearing the mask. After sampling the face masks are removed and immediately placed in a double grip seal bag and stored at 4°C. The filter is then removed and cultured.
10. **Mood assessments** by the Hospital Anxiety and Depression Score will be performed prior to administration of the second challenge to screen for clinical mood concerns ahead of self-isolation/quarantine.
11. **Nasal biopsy tissue** will be collected on a single occasion as an optional procedure. This tissue will be collected from the inferior turbinate and/or the postnasal space/adenoid following NHS procedures. Participants will have up to 4 biopsies collected between the two sites, performed by a trained ENT surgeon in an acute hospital outpatient setting using local anaesthesia.

Clinical samples (self-tests, obtained by research participants)

Study participants will be trained in self-sampling during visit 0. These samples will be conducted at the participant place of residence during their confinement/self-isolation phase. After the first 24 hours, participants will be requested to collect the sample within a 2-hour window of a given time each day. Participants will be instructed to take photos of the samples for documentation of time of sampling. Samples will be stored at home in a biohazard appropriate packaging (supplied by the research team) in a freezer. A tiny temperature tag inside their dedicated cooler bag will record and allow for monitoring of the temperature of the samples when at home and during their transfer to clinic. Participants will return the samples on their subsequent outpatient visit. These time points may be adjusted, for example in the event of afternoon inoculation, to avoid sampling during the night. The purpose of these samples is to assess the kinetics of viral/bacterial infection whilst reducing the burden of repeated outpatient visits for participants. This technique is well established by our group and has previously been approved (REC reference: 18/NW/0481). Self-tests will include:

1. **Nasopharyngeal or Nasal Swab** (as above)
2. **Nasosorption** (as above)
3. **Saliva (as above)**
4. **Symptom diary:** A symptoms diary will be filled in by participants on the day of each inoculation and for the first 21 days after first inoculation. Participants will be asked to grade any symptoms from 0-4 on a Likert scale. This format has been previously utilised in the EHPC studies, therefore this will facilitate direct comparison of symptoms with previously established CHIM models. Further details on clinical symptom scores and how these will be assessed and managed are described in section 12.4.1.

Clinical Symptom Scores

Individual symptom scores will be accumulated for 21 days after the first challenge (D0).

Upper Respiratory Tract Symptoms

A total 'upper respiratory clinical symptom score' will be derived using a four-point scale (0-4 for absent, mild, moderate, severe and requiring emergency department visit or hospitalisation) for each of the following eight respiratory symptoms: sneezing, headache, malaise, fever (37.6°C or above/chills), nasal discharge, nasal obstruction, sore throat and cough according to established methods, giving a maximum clinical severity score of 24. This is an established method for studies of common cold illnesses (49). Symptoms will be recorded at the same time of day and before any procedures such as nasal lavage is performed. More details are provided in Annex 1.

Lower respiratory tract symptoms

An e-diary of lower respiratory tract symptoms (D0 to D21) will also be completed with a scoring system outlined in the diary card (see Annex 1). The purpose of this exercise is to rapidly identify any participant who may be at risk of developing an SAE.

Follow up visits

Follow up visits will be conducted as detailed in Table 2A and 2B. Flexibility around visit windows is described.

9.14 Sample Handling

Samples will be taken as detailed in sections 9.13 and handled as set out in the Laboratory Analysis Plan. Samples will be analysed as described in section 9.10, some may occur on fresh samples, other samples may be frozen to allow analyses to the batched. Samples will be stored for the duration of the study and thereafter if consent is given be transferred to the OVC biobank (see section 9.3). Samples may be stored in appropriate storage locations outside of the University of Oxford for the duration of the study. If participants do not consent to the OVC biobank, the remaining samples will be destroyed at the end of the study.

9.15 Early discontinuation / withdrawal of participants

A participant may be withdrawn from challenge for any of the following reasons:

- Withdrawal of consent to continue in the study (a subject may withdraw his or her consent at any time)
- The study Investigator or Sponsor, for any reason, decides the subject should be withdrawn from the study
- Adverse events, which cannot be tolerated by the subject
- Pregnancy
- Significant non-compliance with the protocol, as determined by the medical monitor(s)

Sponsor decides to discontinue the subject’s participation in the study or to terminate the study

If a subject is withdrawn from the study, where possible, he/she should continue to attend the safety follow-up visits so that data can be collected for the Intention-to-Treat Population. If subjects do not agree to this, then the clinical team will work with them to ensure safe withdrawal from the study, including administration of antibiotics as appropriate. If a participant withdraws during RSV infectious period, it will be recommended that they continue to wear face mask and self-isolate for up to 10 days following RSV challenge. The participant will be offered viral (NAAT) testing during this period should they wish it. If the participant withdraws after Spn inoculation, they will be advised to complete the prescribed course of antibiotics after withdrawal.

9.16 End of Trial definition

The trial will be complete when all assays providing data for primary and secondary endpoints have been completed. For any exploratory endpoints not completed prior to the end of the trial, where appropriate consent is received, samples will be transferred to OVC Biobank and analysed under the auspices of the OVC Biobank protocol and its ethical approval.

10. Study interventions

10.1 Investigational Medicinal Products (IMP)

No investigational medicinal products will be used during this study

10.2 Concomitant medications

The use of concomitant medication prescribed or over the counter, will be recorded in the participants CRF. Prescribed medications such as antipyretics, antibiotics and immunosuppressive agents may impact the study results or may impact the safety of the participant (particularly during the challenge period). It is at the discretion of the study investigators to determine whether withdrawal or temporary exclusion of the participant is required. Participants will be provided with amoxicillin 500mg to be taken TDS for 3 days to reduce density of Spn colonisation if they remain positive for Spn at their visit 7 without 2 consecutive negative samples, if unwell or at clinician discretion as per section 9.10.

10.3 Assessment of compliance

Issues related to compliance will be assessed by healthcare professionals with appropriate training. Any issues with compliance will be recorded and documented according to GCP guidelines.

10.4 Post study interventions

Participants and their GPs will be informed of their study allocation and the order of pathogen challenge.

11. Risk Assessment and Mitigation

This study is designed to ensure minimal risk by applying established safety procedures from the EHPC and RSV models. These safety procedures are described throughout the study protocol but are summarised in Table 4 below. Safety reporting will be consistent with NHS National Research Ethics Service (NRES) and MHRA guidance.

Table 4: Risks and mitigations

**Screening**	Exclude adults with potential risk factors for invasive pneumococcal and severe RSV infection based on history, vital signs, clinical assessment and safety laboratory samples. Exclude adults who have contacts with risk factors for invasive or severe infection. Mood assessment for all participants.
**Participant safety**	Safety guidance is presented to the participant during consent and on the days of inoculation. Participants are provided a safety information leaflet detailing:<br>• contact details for the research team available 24/7<br>• symptoms of infection<br>• advice to report early signs of infection or to seek urgent health care if concerned<br>• if/when to take the antibiotics provided<br>• to report adverse events including unrelated hospital admissions for the duration of the follow up period until their last visit<br>• how to maintain regular hand washing.<br>Close friend or family: participants are encouraged to inform a close contact that they are taking part in a study and a copy of the safety leaflet is provided for them including contact details for the research team. We advise participants that if they are unwell they should contact the clinical research team and inform their identified close contact. The close contact's contact details will be provided to the

	study team should the site be unable to locate the participant. Attempts will be made to contact the close contact instead if there are concerns.
**Confinement or self-isolation**	Phase A: Participants will be subject to confinement for 7-10 days after RSV challenge during the pilot phase of the study to confirm safety of the co-infection challenge. We will educate personnel and participants about the characteristics, transmission, and risk of RSV infection before starting the study. Personal protective equipment (PPE) and handwashing will be provided. Participants in the pilot phase will have an individual room and allowed outdoor (within the facility) wearing a fluid resistant surgical mask and without getting in contact with other participants. Participants will not be allowed physical visitors other than the study staff but will be provided with internet connectivity to facilitate communication.<br><br>Phase B/C: participants will be required to self-isolate at home, again for 7-10 days after RSV challenge, sleeping in a separate room to any co-habitants. Participants will be allowed outside but must wear a fluid resistant surgical mask. Standard self-isolation procedures will be observed, including: staying at home; not going to work or university; not going to public areas; not using public transportation and not having visitors to the house. When possible, we will seek to recruit participants who live <30mins walk away from study facilities to avoid the need for public transport to attend study visits (fluid resistant surgical mask worn during transit). If a participant lives >30mins walk away, they will be considered for the study and would be offered a private taxi (while wearing a fluid resistant surgical mask) or asked to use their own car to avoid contact with the general public. Participants will be asked to maintain a distance of >2m away from household members, remain in a ventilated room with doors closed and if the bathroom and kitchen are shared, there should be a rota to avoid concomitant use and thorough cleaning should be performed after use (more information will be provided in the Participant information sheet). Multiple EHPC studies have demonstrated that there is extremely low risk of bacterial transmission between study participants and their close contacts. Healthy young adults do not suffer from severe RSV infection, but at most develop mild-to-moderate common cold symptoms after experimental RSV infection. Any participant who co-habits with another individual at risk of severe RSV infection (e.g. an infant) will not be eligible for this study (see exclusion criteria). Another human challenge model of RSV conducted in the Netherlands used similar self-isolation guidelines (REF: NL78591.041.21).
**Symptoms and access to healthcare**	**Urgent Care:** to avoid any delay in diagnosis, participants are advised to attend their usual health care facility (wearing a fluid resistant surgical mask) or dial 111 if seriously concerned about their health, as their condition may not necessarily be related to the inoculation. They will also be advised to inform the research team.<br>**Daily checks:** following primary inoculation participants will complete an electronic or paper diary to report any symptoms for a total of 21 days. Thermometers are provided for daily temperature measurement.<br>**Symptoms:** will be monitored and recorded systematically at each visit by the clinical research team.

	**Triggered clinician assessment:** participants with respiratory/ear symptoms, fever, and other symptoms potentially associated with pneumococcal infection may attend the clinic for a triggered assessment by the research nurse/doctor . They may be advised to seek another healthcare route if a visit with the research nurse/doctor is not possible or the symptoms are severe. A research doctor is available for telephone advice 24/7 for participants.<br><br>**General Practitioner:** will be routinely notified of participants involvement in the study.
**Antibiotics**	**Sensitivity:** the bacterial inoculum will be tested to confirm sensitivity to the protocol antibiotics.<br>**Supply of antibiotics:** Oral Amoxicillin 500mg TDS for 3 days is provided to each participant to avoid delay in treatment if the participant has symptoms of potential pneumococcal infection (preferably taken upon discussion with the clinical team).<br>**Termination of colonisation:** participants who remain bacterial colonisation positive at Visit 7 will be advised to take a 3-day course of amoxicillin 500mg TDS antibiotics to clear or reduce colonisation post inoculation.
**Monitoring colonisation**	**Nasal Wash:** results of colonisation are reported to the clinical team each follow-up visit following inoculation. Colonisation and safety data are communicated weekly for discussion by the SMG.
**Withdrawal**	If a participant or their close contacts develop potential risk factors for invasive bacterial or severe viral infection, then they may be withdrawn from the study at any time and commence antibiotics to clear/reduce bacterial colonisation when required.
**Staff Safety**	Use of personal protective equipment, assessment by Occupational Health, management of high-risk staff members (e.g. pregnant or immunocompromised individuals)
**Safety monitoring**	**Trial Oversight:** An established DSMC, TSC and SMG will review infection/colonisation rates and adverse events.

11.1 Risk to participant safety

Bacterial inoculation and colonisation

Pneumococcus is responsible for infections including otitis media (OM), sinusitis, pneumonia, bacteraemia and meningitis. The milder forms of infection (OM, sinusitis) are many times more common than the serious invasive forms of the disease. Due to inoculating participants with pneumococcus, there is a very low risk of OM, sinusitis, pneumonia, bacteraemia and meningitis. While the risk to individuals of developing any infection is very low (10% adults experience natural colonisation at any time and the incidence of invasive disease is 20/100,000 patient years), the study is designed to ensure that any risk is minimal.

This study can be safely run based on the following experience and provisions:

- The research team has 14 years of experience in human challenge studies, following very similar protocols and facing similar risks as previous studies.
- The selected pneumococcal serotype (6B) is fully antibiotic sensitive.
- Participant selection and exclusion criteria reduce the excess risk of invasive pneumococcal disease associated with comorbid conditions.
- Participant education regarding the risks of study participation, provision of a safety information leaflet, and close interaction with the study staff.

- Rigorous and frequent monitoring of development of symptoms and body temperature.
- Provision of standby antibiotics to reduce time to treatment, if it is required.
- 24-hour emergency telephone contact with researchers (including individual daily monitoring for the first 7 days following inoculation), to facilitate access to hospital and/or prompt treatment if required.

We now have experience of inoculating and following over 2000 participants using several serotypes, in different age cohorts and at a range of doses (20,000-320,000 CFU/100µL), with participants being experimentally colonised, naturally colonised and not colonised during our studies. We have had two separate SAEs reported during these challenge studies, both classified as unrelated to the study protocols and study conduct.

In the event that symptoms occur during the inoculation or follow up period, volunteers will be contacted on the day that the symptoms are reported and the clinical team will advise participants to commence amoxicillin based on a pre-defined algorithm (available in the EHPC SOPs) and irrespective of the colonisation status at that point. Participants may be reviewed in study clinic or asked to attend a NHS healthcare treatment facility directly at investigator discretion.

Viral inoculation and colonisation

RSV infection is a common respiratory virus that usually causes mild, cold-like symptoms. The majority of individuals fully recover within 7-14 days. However, RSV infection can be more serious, particularly in young infants and older adults. RSV is the most common cause of pneumonia and bronchiolitis in children under 1 in the UK. In the last decade 12 human challenge studies used the RSV Memphis 37 viral strain, a majority included healthy adults under 60 years of age in 12 days quarantine. Five studies documented mild to moderate adverse events (urticaria, epistaxis, bilateral OM, URTI, pharyngo-laryngeal pain, vomiting, rhinorrhoea, headache, decreased pulmonary function, diarrhoea, GI discomfort), which occurred both in the intervention and placebo groups and were not considered to be related to the viral challenge.

The viral inoculation component of this study can be run safely based on the following experience and provisions:

- The selected RSV-A strain (Memphis 37) is prepared to GMP standards (in line with MHRA guidance) and has been used widely in RSV CHIM studies without any major safety concerns.
- Robust participant selection to reduce the risk of severe infection
- Extensive participant education regarding the risks of study participation, provision of a safety information leaflet, and close interaction with the study staff.
- Rigorous and frequent monitoring of the development of symptoms including body temperature.
- 24-hour emergency telephone contact with researchers (including individual daily monitoring for the first 7 days following inoculation), access to local NHS hospital facilities and prompt treatment if required.

Combined bacterial and viral inoculation and colonisation

This component is novel for a CHIM study. Co-colonisation with RSV and pneumococcus is commonly observed in the community, especially during winter months where RSV is thought to potentiate bacterial colonisation and invasive potential (50). Indeed, we have observed increased pneumococcal colonisation within EHPC studies when RSV has been incidentally detected in nasal wash samples

(8/10 participants). None of these participants experienced any AEs associated with co-infection. However, mindful of the potentially increased risk for participants, we will increase the safety provision for the study, developing specific SOPs during the pilot phase.

Specific, additional safety measures include:

- A pilot phase A incorporating an inpatient confinement period of up to 10 days (up to n=10 participants) post RSV-A inoculation in a dedicated clinical research facility. Previous RSV CHIM studies have demonstrated that any mild coryzal symptoms develop and resolve during this period. During this time, participants will have immediate access to a- healthcare professional 24/7 in the event of illness.
- Development and testing of self-isolation SOPs, based on existing, extensively tested UKHSA procedures, developed and applied for the management of SARS-CoV2 positive individuals during the height of the pandemic.
- Participants admitted to the dedicated clinical research facility will follow the protocols of the admissions process and adhere to local hospital admission assessments as per hospital policy.

Sample collection

The majority of sampling methods utilised are not invasive and have no associated risks. The following have potential for mild, self-limiting risks:

- Nasal Cells: collecting nasal cells may cause discomfort, eye watering or minor local bleeding.
- Nasal Wash: participants may swallow saline which may taste salty.
- Venepuncture: taking blood samples may cause some discomfort or result in a bruise. Very rarely participants experience light-headedness or fainting. Clinical staff are trained to assess and deal with such occurrences. The amount of blood collected during the study will be within the NHS Blood and Transplant guidelines for blood donation
- Throat Swab: participants may gag when the sample is taken.
- Nasopharyngeal/nasal swab and nasosorption: this may cause some discomfort, eye watering or a minor local bleeding.
- Inferior turbinate/post-nasal biopsy: Participants may have mild nasal discomfort or rhinorrhoea following the procedure, which typically settles within 48 hours. Rarely, participants undergoing nasal biopsy may have an adverse reaction to the local anaesthetic used. The risk of local bleeding which may require further outpatient ENT intervention such as nasal packing or cautery is <1%). Rarely, participants may need to be admitted for observation to an ENT acute ward for management of bleeding. Surgical intervention to manage epistaxis in the operating theatre following this procedure is extremely rare. All participants who undergo a nasal biopsy will receive a safety telephone review at 1 day and 3 weeks post-procedure and will have 24/7 access to a study doctor for up to 3 weeks post procedure.

Risks associated with the COVID-19 pandemic

To protect participants, infection control procedures in line with the latest UKHSA guidance will be used throughout the study. Participants who test positive for COVID-19 at the screening visit will be temporarily suspended from the study to reduce the risk of onwards transmission. Samples provided up to that point will be retained and participation will be continued after the isolation period as suggested by UKHSA or longer (if participant remains unwell).

If a participant does develop symptoms suggestive of COVID-19 infection they will be advised to follow the latest UKHSA guidance with regards to self-isolation. A clinical review will be conducted by medical staff with appropriate PPE (for both staff and participant) to clarify whether their symptoms are related to COVID-19 infection, RSV or pneumococcal challenge. This applies to the post inoculation period only. Participants will be advised not to use public transport when travelling to and from this appointment. A COVID-19 nasopharyngeal swab PCR test will be performed at this visit and all further participant study visits will be suspended until this clarified. In the event of a study participant becomes acutely unwell with COVID-19 symptoms, they will be advised to seek urgent medical attention via normal routes of healthcare.

11.2 Risk to participant contacts

To mitigate any potential risk of spreading RSV or pneumococcus to vulnerable groups in the community, we will discuss these risks with the participants and exclude anyone with close physical contact with at risk individuals (i.e. children under 5 years of age, children/adults with immunosuppression or chronic ill health) during the trial period.

As this is the first time that an outpatient RSV challenge study has been conducted in the UK, all household contacts are being invited to take part in screening for RSV during the RSV-challenge phase of the study (twice-weekly swabs for 2 weeks post-challenge).

We prescribe oral amoxicillin 500mg three times daily for 3 days to all participants as per section 9.10 to terminate or reduce colonisation at the end of the participants study period. Self-isolation procedures for up to 10 days after RSV-A challenge are described in Table 4.

11.3 Risk to researchers

Possible risks to researchers include:

- Needle stick injury during venepuncture or nasal biopsy.
- Biological and chemical hazards within the laboratory,
- Infection from colonisation positive participants or inoculum.

Experienced staff will carry out procedures that are within their competencies, as delegated by the Chief/Principal Investigator, and which are in accordance with the relevant SOPs. Appropriate risk and COSHH assessments are in place for clinical and laboratory procedures. All laboratory work will be conducted in an appropriately rated laboratory in line with health and safety regulations for research with human tissues/infectious agents. Participants with COVID-19 symptoms will be advised to self-isolate and not attend further appointments until their COVID-19 status is established. Personal protective equipment including gloves, gown, safety goggles and fluid resistant surgical masks will be used at all study visits according to the established procedures.

12. Safety reporting

12.1 Definitions

Our safety reporting terms and definitions are described in Table 5 below.

**Term**	**Definition**

**Adverse Event (AE)**	Any untoward medical occurrence in a participant to whom an inoculum has been administered, including occurrences, which are not necessarily caused by or related to that product.
**Adverse Event of Special Interest (AESI)**	An adverse event of special interest (serious or non-serious) is one of scientific and medical concern specific to the inoculation, for which ongoing monitoring and rapid communication by the investigator to the Sponsor can be appropriate. Such an event might warrant further investigation in order to characterise and understand it. Depending on the nature of the event, rapid communication by the trial Sponsor to other parties (e.g., regulators, DSMC) might also be warranted.
**Serious Adverse Event (SAE)**	A serious adverse event is any untoward medical occurrence that:<br>• Results in death<br>• Is life-threatening<br>• Requires inpatient hospitalisation or prolongation of existing hospitalisation<br>• Results in persistent or significant disability/incapacity<br>• Consists of a congenital anomaly or birth defect<br><br>Other 'important medical events' may also be considered serious if they jeopardise the participant or require an intervention to prevent one of the above consequences.<br><br>NOTE: The term "life-threatening" in the definition of "serious" refers to an event in which the participant was at risk of death at the time of the event; it does not refer to an event which hypothetically might have caused death if it were more severe.

Table 5: Safety reporting terms. NB: to avoid confusion or misunderstanding of the difference between the terms "serious" and "severe", the following note of clarification is provided: "Severe" is often used to describe intensity of a specific event, which may be of relatively minor medical significance. "Serious" is the regulatory definition supplied above.

12.2 Grading

The labelling of an AE will be defined by the severity threshold described below in Table 6.

Severity grading criteria for local and systemic AEs. NB: A&E assessment in itself does not constitute a SAE. Refer to section 12.1 for SAE definition

**GRADE 0**	None
**GRADE 1**	Mild: Transient or mild discomfort (< 48 hours); No interference with activity; No medical intervention/therapy required
**GRADE 2**	Moderate: Mild to moderate limitation in activity – some assistance may be needed; no or minimal medical intervention/therapy required
**GRADE 3**	Severe: Marked limitation in activity, some assistance usually required; medical intervention/therapy required.
**GRADE 4**	Potentially Life-threatening: requires assessment in A&E or hospitalisation

Table 6: Grading of adverse events

12.3 Causality

The relationship of each adverse event to inoculation must be determined by a medically qualified individual within the site study team according to the following definitions:

Not related	• No temporal relationship to RSV-A or *S. pneumoniae inoculation* ***and***<br>• Alternative aetiology (clinical, environmental or other intervention), ***and***<br>• Does not follow pattern of recognised response to RSV-A or *S. pneumoniae* inoculation or other study procedure.
Possible	• Reasonable temporal relationship to RSV-A or *S. pneumoniae* inoculation, ***or***<br>• Event not readily explained by alternative aetiology (clinical, environmental or other interventions), ***or***<br>• Similar pattern of response to that seen to RSV-A or *S. pneumoniae*.
Probable	• Reasonable temporal relationship to RSV-A or *S. pneumoniae* inoculation or other study procedure, ***and***<br>• Event not readily produced by alternative aetiology (clinical, environment, or other interventions), ***or***<br>• Known pattern of response with RSV-A or *S. pneumoniae* or other study procedure.
Definite	• Reasonable temporal relationship to RSV-A or *S. pneumoniae inoculation* or other study procedure, ***and***<br>• Event not readily produced by alternative aetiology (clinical, environment, or other interventions), ***and***<br>• Known pattern of response to RSV-A or *S. pneumoniae or other study procedure*.

All solicited AEs recorded and graded by the participant will automatically be assumed to be related to inoculation and therefore will not be formally causality assessed. For every unsolicited AE, the PI-delegated investigator will assess the causal relationship of the unsolicited AE.

12.4 Procedure for collecting and recording of adverse events

We will record solicited AEs occurring from the first challenge visit (V1) until 21 days after first challenge visit (V7) that are observed by the investigator or reported by the participant. Therefore, the diary is collected for a total of 21 days. Severity gradings will be in accordance with Annex 2. All SAEs will be recorded from time of enrolment. Participants will also be given the opportunity to report unsolicited AEs in an additional section of the e-diary.

AEs will be recorded in either:

- The e-diary (entry by participant)
- The eCRF (entry by study team)

After V7, only SAEs will be collected. Serious adverse events will be collected from enrolment until the participant completes the study.

All AEs that result in a participant's withdrawal from the study will, subject to participant consent, be followed up, where possible until a satisfactory resolution occurs, or until a non-study related causality is assigned.

AEs will be recorded using the following guidance:

- Pre-existing medical conditions (present before the study start) are considered medical history and should not be recorded as AEs. However, if the participant experiences a worsening or complication of such a condition, the worsening or complication should be recorded as an AE. Investigators should ensure that the AE term recorded captures the change in the condition (e.g., "worsening of")
- Each AE should be recorded to represent a single diagnosis. Accompanying signs or symptoms (including abnormal laboratory values) should not be recorded as additional AEs.
- Changes in laboratory values are only considered to be AEs if they are judged to be clinically significant, for example, if some action or intervention is required. If abnormal laboratory values are the result of pathology for which there is an overall diagnosis, the diagnosis only should be reported as one AE.

The following information will be recorded in the CRF:

- Description of the AE.
- The date of onset and end date.
- Severity of AE
- Assessment of relatedness to study procedure(s) (as judged by a medically qualified investigator).
- Action taken.

It will be left to the investigator's clinical judgment whether an AE is of sufficient severity to require the participant's removal from study. A participant may also voluntarily withdraw from the study due to what he or she perceives as an intolerable AE. If either of these occurs, the participant should undergo an end of study assessment and be given appropriate medical care (e.g. referral to their GP). If required, the investigator can refer the participant directly to hospital if the AE warrants it.

12.4.1 E-diary AEs

Solicited adverse events will be recorded by the participant in an electronic diary graded by the participant alone (Appendix A). Participants will be asked to complete an electronic diary during their first challenge visit until 14 days after the second challenge visit (total 21 days). All solicited AEs recorded and graded by the participant will automatically be assumed to be related to the inoculation and therefore will not be formally causality assessed.

Solicited adverse events will be reviewed daily during the periods of recording as detailed above by the clinical study team. If the clinical team have concerns about the severity or frequency of an event, or a diary is not completed, this will be followed up with the participant by phone or at a scheduled visit. All $\geq$ grade 3 solicited adverse events recorded in the challenge diary will be followed up with the participant by the clinical team in order to monitor for possible stopping rules. Participants will have access to the study team 24 hours a day via the study mobile number, should they have concerns.

12.4.2 Unsolicited adverse events

These may be recorded by the participant in an electronic diary for the same period as specified above. Unsolicited adverse events will be reviewed at clinic visits. If clarification of any event is required then the study nurse or doctor will seek this from the participant during a clinical visit or by telephone call. Unsolicited adverse events recorded in the e-diary will be severity graded by the participant. Causality will be assigned for unsolicited AEs.

In-person clinical reviews may be arranged at the investigator's discretion if there is sufficient clinical concern. This may be at the study site or during a home visit.

12.4.3 Vital sign AEs

At all visits vital signs are taken. These will be recorded directly into the eCRF at the time of review and severity grading will be automatically assigned as per Annex 3. Where a moderate or severe (grade 2 or 3) AE is identified a clinician should review the participant in clinic and document the clinical assessment carried out. Changes in vital signs that are deemed clinically significant by a PI-delegated clinician will be causality assessed.

12.4.4 Laboratory AEs

All laboratory tests will be recorded onto a results eCRF and automatically graded (Annex 4). If a test is deemed clinically significant, it may be repeated, to ensure it is not a single occurrence. If a test remains clinically significant, the participant will be informed and advised with regards to appropriate medical care. Laboratory results can be out of normal range for a number of reasons other than physiological disturbance (e.g. hot weather, delayed transit to processing laboratory). If judged to be clinically significant these will undergo causality assessment.

12.5 Recording and reporting of serious adverse events (SAEs)

Serious adverse reactions/events (SARs/SAEs) will be reported from the time of enrolment until the completion of the study (day 60) and for participants undergoing nasal biopsy, SAEs will be collected for 3 weeks post biopsy.

All SAEs will be recorded on an SAE form and reported to the DSMC chair, Sponsor and the funder Pfizer Inc. within 24 hours of discovery or notification of the event. SAEs will be monitored and reported until the end of the participants last study visit or until resolution/stabilisation. Additional information received for a case (follow-up or corrections to the original case) need to be detailed on an SAE update form and sent to the Sponsor, Pfizer Inc. and the DSMC within 24 hours of new information becoming available. The DSMC will perform an independent review of the SAEs and request any further information required. Documentation of any review will be kept in the site file.

12.6 Adverse Events of Special Interest (AESI)

An adverse event of special interest is one of scientific and medical concern specific to a product or trial, for which ongoing monitoring and rapid communication by the investigator to the safety committee or Sponsor may be appropriate.

Due to the additional study procedures the following events will be considered AESIs.

- Invasive pneumococcal disease
- Severe RSV infection requiring hospitalisation
- Pneumococcal pneumonia, otitis media (OM) or pneumococcal meningitis
- Virologically confirmed transmission of RSV-A to a household contact
- AEs requiring a physician visit or Emergency Department visit which, in the opinion of the study staff, are related to the challenge with RSV-A or Spn

All SAEs and AESIs will be followed until resolution, until the event is considered stable or until a non-study causality is assigned.

12.7 Development Safety Update Reports (DSURs)

The CI will submit a Development Safety Update Report (DSUR) once a year throughout the clinical study, or on request, to REC and Sponsor.

12.8 Stopping rules

Stopping rules for individual participants will apply, which may result in halting progress to second challenge.

- Solicited adverse events: the participant develops a ≥ grade 3 systemic solicited AE considered possibly, probably or definitely related within 2 days after challenge (day of challenge and one subsequent day) which persists for 48 hours or is deemed severe by clinician assessment
- Unsolicited adverse events: the participant has a ≥ grade 3 adverse event, considered possibly, probably or definitely related to challenge which persists for 48 hours or is deemed severe by clinician assessment or has a SAE considered possibly, probably or definitely related to challenge.

The TSC may make the decision to terminate the trial early in the event of serious safety concerns, informed by the DSMC.

12.9 Data and Safety Monitoring Committee (DSMC)

A DSMC is an independent committee which will review safety and colonisation rate data throughout the study. All roles and responsibilities of the DSMC will be outlined in detail in the DSMC charter. The specific role of the committee will be:

- To independently review AEs, SAEs and AESIs regardless of relatedness to any of the study procedures throughout the study.
- To formally review the safety profile and colonisation rate of the inoculum during the pilot phase before progression to the second phase and at the end of the study.

To perform unscheduled reviews on request of the study team at a demand and frequency determined by the severity of reported adverse events.

The DSMC will be provided with a full safety report after 21 days of safety data have been collected from the first 8 participants completing Phase A. For the avoidance of doubt, this will be 21 days of safety data after the last participant has completed Phase A, with the report to be presented within 14 days of this timepoint being reached. This safety report will include any information on solicited and unsolicited adverse events (AEs), laboratory AEs, SAEs and AESI. The DSMC will be asked to make recommendations on whether the study can progress to Phase B/C on the basis of safety data.

The DSMC will be provided with a second formal safety report after 21 days of safety data have been collected from the first 8 participants in outpatient Phase B/C.

A further DSMC meeting will be held at the conclusion of the study,

The DSMC will be supplied with additional reports at the completion of the study, in the event of an SAE, or if requested at any time by the CI or DSMC members.

The outcome of each DSMC review will be communicated directly to the CI, SMG, TSC and Pfizer Inc. and documentation of all reviews will be kept in the site file.

The Chair of the DSMC will also be contacted for advice where the CI feels independent advice or review is required.

13. Statistics and Analysis

13.1 Statistical Analysis Plan

Statistical Analysis will be performed by statisticians within the Oxford vaccine group. The per-protocol population will be used for the primary and secondary analysis. A detailed statistical analysis plan (SAP) will be signed off before any formal analysis of the data.

13.2 Populations for analysis of primary and secondary endpoints

The per-protocol population will be used for evaluation of primary and secondary outcomes. For primary outcome evaluation, the per-protocol population is defined as study participants who:

1. Have consented to the study and met all the inclusion and exclusion criteria;
2. Have been successfully challenged with Spn6B and RSV;
3. Have had three nasal wash data points within 14 days post-inoculation of Spn, including V2, V3 and V6.
4. Have not had prohibited medications during the study
5. Completed 7 days follow-up visit following secondary inoculation

Exploratory endpoints will be analysed in the following populations:

Participants who received at least one challenge with at least one evaluable post-challenge clinical specimen. For some of the analysis, such as gene expression, a subset of participants will be selected for analysis based on clinical outcomes and availability of samples for desired timepoints.

13.3 Analysis of demographics and baseline characteristics

Descriptive statistics relating to participant characteristics at baseline will be calculated overall and by group. No formal statistical comparisons of baseline characteristics between randomised groups will be conducted.

13.4 Data Summaries

Continuous variables such as the density of pneumococcal colonisation, viral load and AUC of density of pneumococcal colonisation over time and RSV viral load over time will be summarised according to number of subjects with non- missing data (n), mean, standard deviation (SD), median, minimum, and maximum.

Categorical variables such as presence/absence and duration of pneumococcal colonisation and RSV infection, AEs and SAEs will be summarised according to the absolute frequency and percentage of subjects (%) in each category level. The denominator for the percentages is the number of subjects in the treatment arm with data available, unless noted otherwise.

13.5 Description of statistical methods

The primary objective is to compare the Spn6B colonisation rates following *Streptococcus pneumoniae* serotype 6B (Spn6B) challenge between participants with or without previous RSV-A challenge by classical culture. The null and alternative hypotheses are:

H_0: Colonisation $rate_{RSV\text{-}P}$ = Colonisation $rate_{P\text{-}RSV}$

H_1: Colonisation $rate_{RSV\text{-}P} \neq$ Colonisation $rate_{P\text{-}RSV}$

where colonisation $rate_{RSV\text{-}P}$ is the proportion of participants with presence of Spn6B experimental *Streptococcus pneumoniae* within 14 days following inoculation at day 7 in the RSV-P arm, and colonisation $rate_{P\text{-}RSV}$ is the proportion of participants with presence of Spn6B within 14 days following inoculation at day 0 in the P-RSV arm. The endpoint evaluation can be found in section 9.6. Immunogenicity data are expected to be highly skewed and will be log-transformed prior to analysis. Results will be presented as geometric means with 95% confidence intervals. Values below the limit of detection will be replaced by half the value of the lower limit.

13.6 Analysis of primary outcome

Primary outcome (presence/absence of Spn6B experimental *Streptococcus pneumoniae* during 21 days post primaryD0 challenge) will be summarised by frequencies and proportions by study arms. Data will be analysed using a generalized linear model with presence/absence of Spn6B being dependent variable and randomisation stratification factor (i.e., study site, and gender) and study arm included as independent variables. The risk ratio and rate difference together with their 95% confidence intervals will be estimated. Data from pilot phase will be included within the analysis of primary outcome.

Natural carriers will be included in the above final analysis. The analysis strategy will follow the procedure above.

13.7 Analysis of secondary outcomes

Pneumococcal density and viral load: Density of pneumococcal colonisation and RSV load at different time points will be available for those who have a recorded positive value and will be analysed in two ways. We will use longitudinal data analysis methods, specifically generalised linear mixed models (GLMM) or generalised estimating equations (GEE), to analysis pneumococcal and RSV colonisation status and density at individual time points with treatment, time and interaction between time and treatment as fixed effects and study participant as random effect / clustering variable. The density will be summarised using number, mean, geometric mean, standard deviation, median, minimum and maximum at each time point. Exchangeable covariance structure will be used. The geometric mean ratio between groups together with their 95% CIs at each time point will be derived.

The area under the curve (AUC) of density of experimental pneumococcal colonisation over time or RSV load over time will be derived and summarised using the above descriptive statistics and log AUC will be analysed using a generalised linear model with a single factor of treatment. The geometric mean ratio between groups together with their 95% CIs will be derived.

RSV attack rate: The binary secondary endpoints (presence/absence of RSV-A at any time point during 21 days post primary D0 challenge) will follow the primary analysis.

Symptoms, signs and safety after co-infection challenge: The symptoms between the sole-infection challenge period and co-infection challenge period of the same challenge agent will be compared. E.g. for the impact of co-infection challenge on symptoms following Spn6B challenge, we will compare the D0-D3 symptoms in the Spn-RSV arm to the D4-D7 symptoms in the RSV-Spn arm. Symptoms after D9 will be summarised on the descriptive basis. To model the impact of co-infection challenge, the

individual symptom score at each day will be the dependent variable (absent vs present), and study group (sole-infection and co-infection), study site, time (days following the challenge of interest, D0-D3) will be included in the model as independent variables. Participant level random effects will be included in the model. We will also explore the interaction between study arms and time by including an interaction term in the model.

Other secondary outcomes: The analysis of all the other secondary outcomes will be included in the SAP.

13.8 Analysis of exploratory outcomes

The immune responses data are expected to be highly skewed, and the data will be log-transformed prior to analysis. The geometric mean concentration (GMC) and associated 95% confidence interval (CI) will be summarised by computing the anti-log of the mean of the log-transformed data. Data will be summarised by study arms at different time points.

13.9 Sample size determination

Phase A

No power calculation has been conducted for the pilot (feasibility and safety) phase where up to 10 participants will be randomised (1:1) to primary RSV or Spn challenge with reciprocal challenge seven days later. With an anticipated drop-out rate of 20%, the effective sample size will be 8.

Phase B/C

The primary outcome measure for this study is experimental induction of pneumococcal carriage with serotype 6B (Spn). Based on our extensive programme of controlled human pneumococcal infection research in over 2000 volunteers, we have demonstrated that 50% of participants develop experimental carriage (classical microbiological culture confirmation) after experimental exposure to serotype 6B. During this programme, we have observed that 5/6 participants in whom asymptomatic RSV infection (confirmed by nasal PCR) was identified prior to pneumococcal challenge subsequently developed experimental carriage. Based on these data, and using a two-sided proportions power calculation test (parameters: alpha =0.05 and 80% power), 45 participants will be required to demonstrate a difference of 30% Spn carriage in two independent groups: 1) primary RSV challenge followed by Spn6B challenge (expected proportion of pneumococcal carriage +ve = 80%); and 2) primary Spn6B challenge followed by RSV challenge (expected proportion of pneumococcal carriage +ve = 50%). The effective sample size will be 90 (including participants who completed phase A). After adjusting for an attrition of 20%, the study will recruit a total of up to 113 participants. Anticipated RSV attack rates will be 55%-60% (personal communication Prof Chris Chiu). We do not anticipate this being altered by preceding pneumococcus colonisation. RSV attack rate were therefore not used for sample size estimations (primary outcome).

13.10 Decision points

An interim analysis is not planned for this study. Decision points to pause or terminate the trial will be made based on participant safety data if any concerns emerge.

13.11 The Level of Statistical Significance

Statistical significance will be assumed if $p < 0.05$ for primary and secondary outcome measures after appropriate adjustment if multiple tests are used.

13.12 Procedure(s) to Account for Missing or Spurious Data

Reasons for missing data (including withdrawal of consent, loss to follow-up, removal from study due to serious side effects, death, or inability to obtain any laboratory results) will be indicated, but missing data will not be imputed for primary endpoints. Missing data for colonisation density or immune responses will be interpolated where possible, otherwise (as when participants are excluded at an early timepoint within the study), removed from the analysis.

13.13 Procedures for Reporting any Deviation(s) from the Original Statistical Plan

A final SAP will be signed off before the final database lock. Any additional analysis or deviations from the SAP will be documented in the final analysis report and updated according to the statistical SOP.

14. Data Management

The data management aspects of the study are summarised here, with details fully described in the Data Management Plan.

The Investigators will populate the content of the participants' CRFs, which will be in a paper and/or electronic format using an EDC system (e.g. REDCap database, or an appropriate alternative). The database will be stored on a secure server located in Europe and will have restricted access (password-protection) and accountability records. All information transcribed to and from the REDCap database will be done by encrypted (https) transfer.

Each study participant will have a unique participant number which will be allocated at the time of the screening visit. Names and/or identifying details are not included in any study data electronic file, with the exception of the electronic diaries, for which consent will be obtained to store the participant email address, which is necessary for the system to function. Only site research staff and sponsor data managers have access to view the email address. With the exception of clinical safety blood samples which are sent to local clinical laboratories and follow local sample labelling requirements, samples sent to laboratories for processing will be identified by trial number and participant number only.

14.1 Data integrity

Data collection and storage will be inspected throughout the study by internal monitors appointed by the Oxford Vaccine Group on behalf of the study Sponsor, University of Oxford Research Governance, Ethics and Assurance (RGEA). An external independent auditor appointed by Sponsor and funder in collaboration may also audit the study data.

14.2 Data storage

Study data may be stored electronically on a secure server, and paper notes will be kept in a key-locked filing cabinet at the study site. All essential documents will be retained for up to 25 years or as per national regulatory requirements. Volunteers who only complete online screening or telephone screening (before informed consent) will not have data kept beyond the end of the trial. The need to store study data for longer in relation to potentially relevant future vaccines will be subject to ongoing review. Pseudonymised research data may be stored indefinitely, but with 5 yearly review. General archiving procedures will be conducted in compliance to SOP OVC020 Archiving.

14.3 Source data

Source documents are original documents, data, and records from which participants' CRF data are populated. These include, but are not limited to, hospital or GP records (from which medical history and previous and concurrent medication may be summarised into the CRF), clinical and office charts, laboratory and pharmacy records, diaries, microfiches, radiographs, and correspondence. In this study, CRF entries will be considered source data where it is the site of the original recording. All documents will be stored safely under strict confidentiality and with restricted access. On all study-specific documents, other than the signed consent and the participant contact sheet, the participant will be referred to by the study participant number/code only.

Participant's personally identifiable information will be stored in a separate password protected databases (Participant Management System in Oxford and AccessionQ in Liverpool) saved on secure servers within the institutions. Only Oxford staff and Liverpool staff have access to the Participant management system and AccessionQ, respectively, and have permission for data entry.

14.4 Access to data

Direct access will be granted to authorised representatives from (or appointed by) the Sponsor, host institution and the regulatory authorities to permit trial-related monitoring, audits and inspections.

14.5 Data recording and record keeping

The Investigators will populate the content of participants' CRFs and all the study data including participants e-diaries will be recorded directly into an Electronic Data Capture (EDC) system (e.g. REDCap, or similar), or onto a paper source document for later entry into the EDC system if direct entry is not available. Any additional information that needs recording but is not relevant for the CRF (such as signed consent forms) will be recorded on a separate paper source document. All documents will be stored safely and securely in confidential conditions.

The EDC system (CRF data) uses a relational database (MySQL/ PostgreSQL) via a secure web interface with data checks applied during data entry to ensure data quality. The database includes a complete suite of features which are compliant with GCP, EU and UK regulations and Sponsor security policies, including a full audit trail, user-based privileges, and integration with the institutional LDAP server. The MySQL and PostgreSQL database and the webserver will both be housed on secure servers maintained by Oxford Vaccine Group IT personnel. The servers are in a physically secure location in Europe, and data are backed up on secure servers operated by the University of Oxford IT Services, physically located in Europe and will have restricted access (password-protection) and accountability records. Backups will be stored in accordance with the IT department schedule of daily, weekly, and monthly retained for one month, three months, and six months, respectively. Weekly backup tapes are stored offsite. The servers provide a stable, secure, well-maintained, and high-capacity data storage environment. REDCap is a widely used, powerful, reliable, well-supported system. Access to the study's database will be restricted to the members of the study team by username and password.

The study team will use names and contact details to contact participants about the research study, and make sure that relevant information about the study is recorded for their care, in relation to their health during the study and to oversee the quality of the study. At the completion of the study, unless

participants consent otherwise (e.g. requesting to be informed of other trials), participant's personal details will not be used to contact them other than in exceptional circumstances concerning their safety. If consent is provided by participants to take part in another study carried out by the study site, personal information and medical information including blood test results may be accessed to avoid unnecessary repetition. If participants provide specific consent, we will use personal identifiable data to invite participants for future research.

Each study participant will have a unique participant number which will be allocated at the time of screening. Names and/or identifiable details are not included in the clinical electronic database capture system. Storage of participant email addresses for electronic diaries and electronic medical records access informed consent forms will be required for the system to function, which consent will be obtained. Only site research staff and sponsor data managers have access to view the email address.

Bank details will be stored for time limited periods according to the site policies.

15. Quality assurance procedures

A Trial Monitoring Plan will be developed by the OVG monitors and agreed by the Study Management Group (SMG) and CI based on the trial risk assessment. The frequency of monitoring will be dependent on a documented risk assessment of the trial after review by the Sponsor. Monitoring will be performed according to ICH Good Clinical Practice (GCP) by OVG Monitor. Following written SOPs, the monitors will verify that the clinical trial is conducted, and data are generated, documented and reported in compliance with the protocol, GCP and the applicable regulatory requirements. The investigational site will provide direct access to all trial related source data/documents, eCRFs and reports for the purpose of monitoring and auditing by the sponsor and inspection by local and regulatory authorities.

15.1 Risk assessment

A protocol risk assessment and monitoring plan is prepared before the finalisation of the protocol and will be reviewed as necessary over the course of the study to reflect significant changes to the protocol or outcomes of monitoring activities.

15.2 Study monitoring

Monitoring will be performed according to the principles of Good Clinical Practice (GCP) by parties appointed by OVG. The investigator sites will provide direct access to all trial related source data/documents and reports for the purpose of monitoring and auditing by the Sponsor and inspection by local and regulatory authorities.

15.3 Study committees

Study Management Group (SMG):

Includes scientists, health professionals and investigators who provide ongoing management of the trial. They conduct the study and review recruitment, safety and colonisation reports weekly.

Trial Steering Committee (TSC):

Provides oversight on trial design, safety, conduct and evaluation consistent with GCP guidelines in accordance with terms of reference. The committee is comprised of members of the SMG as well as members who are independent to the conduct of the clinical trial (which includes the chair) who have expertise in the management of controlled human infection model studies. The TSC consider recommendations from the Data and Safety Monitoring Committee (DSMC) then advise the SMG and Sponsor. The TSC may make major decisions including to terminate the study, replace an arm of the study or amend the protocol.

Data and Safety Monitoring Committee (DSMC):

The DSMC safeguards and monitors the interests of the trial participants by assessing the safety of interventions and review the protocol according to the DSMC charter. They periodically review safety data to determine patterns and trends of events, or to identify safety issues, which would not be apparent on an individual case basis. They may review data in the interest of safety. Members are independent to the trial, experienced in this field and the conduct of clinical trials. The TSC and DSMC will be provided with interim safety data on a weekly basis.

The first specific go, no-go review point will take place after 21 days of safety data have been collected from the first 8 participants enrolled in phase A, before progression to the outpatient phase of the study is permitted. A second go, no-go review point will take place after 21 days of safety data have been collected from the first 8 participants in outpatient phase B/C.

In addition, interim data will be provided if at any time the SMG have any concerns regarding the safety of a participant or the general public. The DMSC will advise the TSC and study investigators on whether there are any ethical or safety reasons why the trial should be changed or not continue. The DSMC will meet as per the terms of reference.

16. Protocol deviations

Any deviations from the protocol will be documented in a protocol deviation form and filed in the trial master file. Each deviation will be assessed as to its impact on volunteer safety and study conduct. Significant deviations will be listed in the end of study report.

16.1 Audit and Inspection

The QA manager at OVG conducts systems based internal audits to check that trials are being conducted according to local procedures and in compliance with GCP and applicable regulations.

The Sponsor, trial sites, and ethical committee(s) may carry out audits to ensure compliance with the protocol, GCP and appropriate regulations.

GCP inspections may also be undertaken by the MHRA to ensure compliance with protocol and the Medicines for Human Use (Clinical Trials) Regulations 2004, as amended. The Sponsor will assist in any inspections and will support the response to the MHRA as part of the inspection procedure.

17. Serious breaches

A "serious breach" is defined as breach which is likely to affect to a significant degree the safety or physical or mental integrity of the participants or the scientific value of the trial.

The Sponsor will be notified immediately of any case of a serious breach where the above definition applies during the trial conduct phase. The Sponsor of a clinical trial will notify the ethics committee in writing of any serious breaches of the conditions and principles of GCP in connection with that trial; or the protocol relating to that trial, as amended from time to time, within 7 days of becoming aware of that breach. Pfizer will be notified of any confirmed serious breaches within 7 days.

18. Ethical and regulatory considerations

18.1 Declaration of Helsinki

The Investigator will ensure that this trial is conducted in accordance with the principles of the Declaration of Helsinki and in line with GCP guidelines.

18.2 Guidelines for good clinical practice

The Investigator will ensure that this study is conducted in accordance with relevant regulations and with Good Clinical Practice.

18.3 Approvals

Following Sponsor approval the protocol, informed consent form, participant information sheet and. advertising material will be submitted to an appropriate Research Ethics Committee (REC); HRA (if relevant); and host institutions for written approval. The Investigator will submit and, where necessary, obtain approval from the above parties for all substantial amendments to the original approved documents.

18.4 Other ethical considerations

We will not recruit potentially vulnerable participants to this project. All participants must demonstrate capacity to consent for themselves. Participants will be asked to complete a short quiz (demonstrative that details in the participant information sheet have been understood and retained) prior to completion of the informed consent documentation. If we identify clinically relevant findings during the study, we will communicate these to the participant and support linkage to NHS care, including preparation of medical records summarising issues identified, in partnership with the participant.

18.5 Reporting

All correspondence with the REC will be retained in the Trial Master File/Investigator Site File. An annual progress report (APR) will be submitted by the Chief Investigator to the REC within 30 days of the anniversary date on which the favourable opinion was given, and annually until the trial is declared ended. Within one year after the end of the trial, the Chief Investigator will submit a final report with the results, including any publications/abstracts, to the REC. If the trial in ended prematurely, the Chief Investigator will notify the REC, including the reasons for the premature termination.

18.6 Transparency in research

Prior to the recruitment of the first participant, the study will have been registered on a publicly accessible database. Results will be uploaded to the databases within 12 months of the end of trial declaration by the CI or their delegate. Where the trial has been registered on multiple public

platforms, the trial information will be kept up to date during the trial, and the CI or their delegate will upload results to all those public registries within 12 months of the end of the trial declaration.

18.7 Participant confidentiality

The study will comply with the General Data Protection Regulation (GDPR) and Data Protection Act 2018, which require data to be anonymised as soon as it is practical to do so. The trial staff will ensure that the participants' anonymity is maintained other than for uses (e.g. communication with the GP) about which the participants will be specifically consented for. Participants will be identified by a participant ID number on the CRF. Any electronic databases and documents with participant identifying details will be stored securely and will only be accessible by study staff and authorised personnel.

18.8 Expenses and benefits

It is not intended that financial factors influence an individual's decision to participate in this study. The fees will reflect remuneration and not financial coercion. We compensate participants for time, travel, inconvenience and discomfort. The sums offered are consistent with remuneration in other similar local and national studies and are detailed below:

Time per visit	£40 per hour
Travel expenses per visit	£30
Sample collection per visit	£20
Diary completion	£60
Inpatient period per day	£200
Self-isolation period per day	£150
Nasal Biopsy visit	£150

Remuneration is on a *pro rata* basis should a participant not complete all visits and/or study requirements. If a participant withdraws from the study early, they will be remunerated for the visits they attended and samples which were taken up until the time they withdrew. If additional visits are required, the participant may be reimbursed for that visit. Standard visits are up to 60 minutes whereas screening/inoculation visits have a duration of up to 90 minutes. Each participant in phase A can therefore receive a maximum of £3,130 and each participant in phase B/C a maximum of £2,580. Household contacts who consent to twice-weekly swabbing will be offered £10 per swab completed. This may be in the form of a voucher.

19. Finance and insurance

19.1 Funding

Funding for the study has been provided by Pfizer Inc.

19.2 Insurance

The University has a specialist insurance policy in place which would operate in the event of any participant suffering harm as a result of their involvement in the research (Newline Underwriting Management Ltd, at Lloyd's of London).

19.3 Contractual arrangements

Appropriate contractual arrangements will be put in place with all third parties.

20. Publication policy

20.1 Publications

A publication is defined as any written document intended for submission to a congress, conference, journal or other public forum, and includes abstracts, posters and full articles, pertaining to the study, with or without study results. Publications will be consistent with the Consort Guidelines and checklist http://www.consort-statement.org/ and will be based on the International Committee of Medical Journal Editors (ICJME) requirements in that all persons listed as authors must meet ICJME requirements and all persons that meet these requirements will be listed as an author, other contributors will be acknowledged.

The findings from this study will be disseminated amongst the scientific community. We intend to publish our findings in peer reviewed scientific journals and present data at appropriate local, national and international conferences. In addition, we will produce a lay report of our findings, which will be made available to all participants.

20.2 Authorship

Authorship of the final trial report and subsequent publications will include those who contribute to the design, delivery and analysis of the trial in the University of Oxford and LSTM teams and collaborators at Pfizer Inc. Authorship will be defined on completion of the study in discussion with Professor Daniela Ferreira.

21. Process for the generation of intellectual property

Ownership of IP generated by employees of the University vests in the University. The University will ensure appropriate arrangements are in place as regards any new IP arising from the study.

22. Archiving

Study data may be stored electronically on a secure server and paper notes will be kept in a secure location at the site. All essential documents will be retained for up to 25 years, or as per national regulatory requirements. Pseudonymised research data may be stored indefinitely, but with 5 yearly review. General archiving procedures will be conducted in compliance to SOP OVC020 Archiving.

23. References

1. Geoghegan S, Erviti A, Caballero MT, Vallone F, Zanone SM, Losada JV, Bianchi A, Acosta PL, Talarico LB, Ferretti A, Grimaldi LA, Sancilio A, Duenas K, Sastre G, Rodriguez A, Ferrero F, Barboza E, Gago GF, Nocito C, Flamenco E, Perez AR, Rebec B, Ferolla FM, Libster R, Karron RA, Bergel E, Polack FP. Mortality due to Respiratory Syncytial Virus. Burden and Risk Factors. *Am J Respir Crit Care Med* 2017; 195: 96-103.

2. Wahl B, O'Brien KL, Greenbaum A, Majumder A, Liu L, Chu Y, Lukšić I, Nair H, McAllister DA, Campbell H, Rudan I, Black R, Knoll MD. Burden of Streptococcus pneumoniae and Haemophilus influenzae type b disease in children in the era of conjugate vaccines: global, regional, and national estimates for 2000-15. *Lancet Glob Health* 2018; 6: e744-e757.

3. Weinberger DM, Klugman KP, Steiner CA, Simonsen L, Viboud C. Association between respiratory syncytial virus activity and pneumococcal disease in infants: a time series analysis of US hospitalization data. *PLoS Med* 2015; 12: e1001776.

4. Weinberger DM, Givon-Lavi N, Shemer-Avni Y, Bar-Ziv J, Alonso WJ, Greenberg D, Dagan R. Influence of pneumococcal vaccines and respiratory syncytial virus on alveolar pneumonia, Israel. *Emerg Infect Dis* 2013; 19: 1084-1091.

5. Bardsley M, Morbey RA, Hughes HE, Beck CR, Watson CH, Zhao H, Ellis J, Smith GE, Elliot AJ. Epidemiology of respiratory syncytial virus in children younger than 5 years in England during the COVID-19 pandemic, measured by laboratory, clinical, and syndromic surveillance: a retrospective observational study. *The Lancet Infectious Diseases* 2023; 23: 56-66.

6. Pollard AJ, Savulescu J, Oxford J, Hill AV, Levine MM, Lewis DJ, Read RC, Graham DY, Sun W, Openshaw P, Gordon SB. Human microbial challenge: the ultimate animal model. *Lancet Infect Dis* 2012; 12: 903-905.

7. Bagga B, Cehelsky JE, Vaishnaw A, Wilkinson T, Meyers R, Harrison LM, Roddam PL, Walsh EE, DeVincenzo JP. Effect of Preexisting Serum and Mucosal Antibody on Experimental Respiratory Syncytial Virus (RSV) Challenge and Infection of Adults. *J Infect Dis* 2015; 212: 1719-1725.

8. DeVincenzo JP, Wilkinson T, Vaishnaw A, Cehelsky J, Meyers R, Nochur S, Harrison L, Meeking P, Mann A, Moane E, Oxford J, Pareek R, Moore R, Walsh E, Studholme R, Dorsett P, Alvarez R, Lambkin-Williams R. Viral load drives disease in humans experimentally infected with respiratory syncytial virus. *Am J Respir Crit Care Med* 2010; 182: 1305-1314.

9. Ascough S, Dayananda P, Kalyan M, Kuong SU, Gardener Z, Bergstrom E, Paterson S, Kar S, Avadhan V, Thwaites R, Sanchez Sevilla Uruchurtu A, Ruckwardt TJ, Chen M, Nair D, Derrien-Colemyn A, Graham BS, Begg M, Hessel E, Openshaw P, Chiu C. Divergent age-related humoral correlates of protection against respiratory syncytial virus infection in older and young adults: a pilot, controlled, human infection challenge model. *Lancet Healthy Longev* 2022; 3: e405-e416.

10. Anderson LJ, Walsh EE. The Challenge of Respiratory Syncytial Virus Human Challenge Studies. *N Engl J Med* 2022; 386: 696-697.

11. Sadoff J, De Paepe E, DeVincenzo J, Gymnopoulou E, Menten J, Murray B, Rosemary Bastian A, Vandebosch A, Haazen W, Noulin N, Comeaux C, Heijnen E, Eze K, Gilbert A, Lambkin-Williams R, Schuitemaker H, Callendret B. Prevention of Respiratory Syncytial Virus Infection in Healthy Adults by a Single Immunization of Ad26.RSV.preF in a Human Challenge Study. *J Infect Dis* 2022; 226: 396-406.

12. DeVincenzo J, Cass L, Murray A, Woodward K, Meals E, Coates M, Daly L, Wheeler V, Mori J, Brindley C, Davis A, McCurdy M, Ito K, Murray B, Strong P, Rapeport G. Safety and Antiviral

Effects of Nebulized PC786 in a Respiratory Syncytial Virus Challenge Study. *J Infect Dis* 2022; 225: 2087-2096.

13. Schmoele-Thoma B, Zareba AM, Jiang Q, Maddur MS, Danaf R, Mann A, Eze K, Fok-Seang J, Kabir G, Catchpole A, Scott DA, Gurtman AC, Jansen KU, Gruber WC, Dormitzer PR, Swanson KA. Vaccine Efficacy in Adults in a Respiratory Syncytial Virus Challenge Study. *N Engl J Med* 2022; 386: 2377-2386.

14. release PP. Pfizer Announces Positive Top-Line Data from Phase 3 Trial of Older Adults for its Bivalent Respiratory Syncytial Virus (RSV) Vaccine Candidate. 2022. Available from: https://www.pfizer.com/news/press-release/press-release-detail/pfizer-announces-positive-top-line-data-phase-3-trial-older.

15. Roestenberg M, Hoogerwerf MA, Ferreira DM, Mordmuller B, Yazdanbakhsh M. Experimental infection of human volunteers. *Lancet Infect Dis* 2018; 18: e312-e322.

16. Ferreira DM, Neill DR, Bangert M, Gritzfeld JF, Green N, Wright AK, Pennington SH, Bricio-Moreno L, Moreno AT, Miyaji EN, Wright AD, Collins AM, Goldblatt D, Kadioglu A, Gordon SB. Controlled human infection and rechallenge with Streptococcus pneumoniae reveals the protective efficacy of carriage in healthy adults. *Am J Respir Crit Care Med* 2013; 187: 855-864.

17. Nikolaou E, Jochems SP, Mitsi E, Pojar S, Blizard A, Reiné J, Solórzano C, Negera E, Carniel B, Soares-Schanoski A, Connor V, Adler H, Zaidi SR, Hales C, Hill H, Hyder-Wright A, Gordon SB, Rylance J, Ferreira DM. Experimental Human Challenge Defines Distinct Pneumococcal Kinetic Profiles and Mucosal Responses between Colonized and Non-Colonized Adults. *mBio* 2021; 12.

18. Mitsi E, Roche AM, Reine J, Zangari T, Owugha JT, Pennington SH, Gritzfeld JF, Wright AD, Collins AM, van Selm S, de Jonge MI, Gordon SB, Weiser JN, Ferreira DM. Agglutination by anti-capsular polysaccharide antibody is associated with protection against experimental human pneumococcal carriage. *Mucosal Immunol* 2016.

19. Adler H, German EL, Mitsi E, Nikolaou E, Pojar S, Hales C, Robinson R, Connor V, Hill H, Hyder-Wright AD, Lazarova L, Lowe C, Smith EL, Wheeler I, Zaidi SR, Jochems SP, Loukov D, Reine J, Solorzano-Gonzalez C, de Gorguette d'Argoeuves P, Jones T, Goldblatt D, Chen T, Aston SJ, French N, Collins AM, Gordon SB, Ferreira DM, Rylance J. Experimental Human Pneumococcal Colonization in Older Adults Is Feasible and Safe, Not Immunogenic. *Am J Respir Crit Care Med* 2021; 203: 604-613.

20. Wright AK, Bangert M, Gritzfeld JF, Ferreira DM, Jambo KC, Wright AD, Collins AM, Gordon SB. Experimental human pneumococcal carriage augments IL-17A-dependent T-cell defence of the lung. *PLoS Pathog* 2013; 9: e1003274.

21. Mitsi E, Kamng'ona R, Rylance J, Solorzano C, Jesus Reine J, Mwandumba HC, Ferreira DM, Jambo KC. Human alveolar macrophages predominately express combined classical M1 and M2 surface markers in steady state. *Respir Res* 2018; 19: 66.

22. Carniel BF, Marcon F, Rylance J, German EL, Zaidi S, Reine J, Negera E, Nikolaou E, Pojar S, Solorzano C, Collins AM, Connor V, Bogaert D, Gordon SB, Nakaya HI, Ferreira DM, Jochems SP, Mitsi E. Pneumococcal colonization impairs mucosal immune responses to live attenuated influenza vaccine. *JCI Insight* 2021; 6.

23. Mitsi E, Carniel B, Reine J, Rylance J, Zaidi S, Soares-Schanoski A, Connor V, Collins AM, Schlitzer A, Nikolaou E, Solorzano C, Pojar S, Hill H, Hyder-Wright AD, Jambo KC, Oggioni MR, De Ste Croix M, Gordon SB, Jochems SP, Ferreira DM. Nasal Pneumococcal Density Is Associated with Microaspiration and Heightened Human Alveolar Macrophage Responsiveness to Bacterial Pathogens. *Am J Respir Crit Care Med* 2020; 201: 335-347.

24. Peno C, Banda DH, Jambo N, Kankwatira AM, Malamba RD, Allain TJ, Ferreira DM, Heyderman RS, Russell DG, Mwandumba HC, Jambo KC. Alveolar T-helper 17 responses to streptococcus pneumoniae are preserved in ART-untreated and treated HIV-infected Malawian adults. *J Infect* 2018; 76: 168-176.

25. Diniz MO, Mitsi E, Swadling L, Rylance J, Johnson M, Goldblatt D, Ferreira D, Maini MK. Airway-resident T cells from unexposed individuals cross-recognize SARS-CoV-2. *Nat Immunol* 2022; 23: 1324-1329.

26. Pennington SH, Pojar S, Mitsi E, Gritzfeld JF, Nikolaou E, Solorzano C, Owugha JT, Masood Q, Gordon MA, Wright AD, Collins AM, Miyaji EN, Gordon SB, Ferreira DM. Polysaccharide-specific Memory B-cells Predict Protection Against Experimental Human Pneumococcal Carriage. *Am J Respir Crit Care Med* 2016.

27. Collins AM, Wright AD, Mitsi E, Gritzfeld JF, Hancock CA, Pennington SH, Wang D, Morton B, Ferreira DM, Gordon SB. First human challenge testing of a pneumococcal vaccine. Double-blind randomized controlled trial. *Am J Respir Crit Care Med* 2015; 192: 853-858.

28. Dagan R, Patterson S, Juergens C, Greenberg D, Givon-Lavi N, Porat N, Gurtman A, Gruber WC, Scott DA. Comparative immunogenicity and efficacy of 13-valent and 7-valent pneumococcal conjugate vaccines in reducing nasopharyngeal colonization: a randomized double-blind trial. *Clin Infect Dis* 2013; 57: 952-962.

29. Robinson RE, Mitsi E, Nikolaou E, Pojar S, Chen T, Reine J, Nyazika TK, Court J, Davies K, Farrar M, Gonzalez-Dias P, Hamilton J, Hill H, Hitchens L, Howard A, Hyder-Wright A, Lesosky M, Liatsikos K, Matope A, McLenaghan D, Myerscough C, Murphy A, Solorzano C, Wang D, Burhan H, Gautam M, Begier E, Theilacker C, Beavon R, Anderson AS, Gessner BD, Gordon SB, Collins AM, Ferreira DM. Human Infection Challenge with Serotype 3 Pneumococcus. *Am J Respir Crit Care Med* 2022.

30. Liatsikos K, Hyder-Wright A, Pojar S, Chen T, Wang D, Davies K, Myerscough C, Reine J, Robinson RE, Urban B, Mitsi E, Solorzano C, Gordon SB, Quinn A, Pan K, Anderson AS, Theilacker C, Begier E, Gessner BD, Collins A, Ferreira DM, group Ps. Protocol for a phase IV double-blind randomised controlled trial to investigate the effect of the 13-valent pneumococcal conjugate vaccine and the 23-valent pneumococcal polysaccharide vaccine on pneumococcal colonisation using the experimental human pneumococcal challenge model in healthy adults (PREVENTING PNEUMO 2). *BMJ Open* 2022; 12: e062109.

31. Rylance J, de Steenhuijsen Piters WAA, Mina MJ, Bogaert D, French N, Ferreira DM. Two Randomized Trials of the Effect of Live Attenuated Influenza Vaccine on Pneumococcal Colonization. *Am J Respir Crit Care Med* 2019; 199: 1160-1163.

32. Jochems SP, Marcon F, Carniel BF, Holloway M, Mitsi E, Smith E, Gritzfeld JF, Solórzano C, Reiné J, Pojar S, Nikolaou E, German EL, Hyder-Wright A, Hill H, Hales C, de Steenhuijsen Piters WAA, Bogaert D, Adler H, Zaidi S, Connor V, Gordon SB, Rylance J, Nakaya HI, Ferreira DM. Inflammation induced by influenza virus impairs human innate immune control of pneumococcus. *Nat Immunol* 2018; 19: 1299-1308.

33. Glennie S, Gritzfeld JF, Pennington SH, Garner-Jones M, Coombes N, Hopkins MJ, Vadesilho CF, Miyaji EN, Wang D, Wright AD, Collins AM, Gordon SB, Ferreira DM. Modulation of nasopharyngeal innate defenses by viral coinfection predisposes individuals to experimental pneumococcal carriage. *Mucosal Immunol* 2016; 9: 56-67.

34. Lewnard JA, Bruxvoort KJ, Fischer H, Hong VX, Grant LR, Jodar L, Gessner BD, Tartof SY. Prevention of Coronavirus Disease 2019 Among Older Adults Receiving Pneumococcal Conjugate Vaccine Suggests Interactions Between Streptococcus pneumoniae and Severe Acute Respiratory Syndrome Coronavirus 2 in the Respiratory Tract. *J Infect Dis* 2022; 225: 1710-1720.

35. Mitsi E, Reine J, Urban BC, Solorzano C, Nikolaou E, Hyder-Wright AD, Pojar S, Howard A, Hitchins L, Glynn S, Farrar MC, Liatsikos K, Collins AM, Walker NF, Hill HC, German EL, Cheliotis KS, Byrne RL, Williams CT, Cubas-Atienzar AI, Fletcher TE, Adams ER, Draper SJ, Pulido D, Beavon R, Theilacker C, Begier E, Jodar L, Gessner BD, Ferreira DM. Streptococcus pneumoniae colonization associates with impaired adaptive immune responses against SARS-CoV-2. *J Clin Invest* 2022.

36. Huijts SM, Coenjaerts FEJ, Bolkenbaas M, van Werkhoven CH, Grobbee DE, Bonten MJM. The impact of 13-valent pneumococcal conjugate vaccination on virus-associated community-acquired pneumonia in elderly: Exploratory analysis of the CAPiTA trial. *Clin Microbiol Infect* 2018; 24: 764-770.

37. al. GBe. A post-hoc analysis of 13-valent pneumococcal conjugate vaccine efficacy against endemic human coronavirus-associated pneumonia. . *Paper presented at: The ESCMID Conference on Coronavirus Disease (ECCVID); September 23–25, 2020; Virtual Accessed March 18, 2022* 2022.

38. Nunes MC, Cutland CL, Klugman KP, Madhi SA. Pneumococcal Conjugate Vaccine Protection against Coronavirus-Associated Pneumonia Hospitalization in Children Living with and without HIV. *mBio* 2021; 12.

39. Greenberg D, Givon-Lavi N, Faingelernt Y, Ben-Shimol S, Avni YS, Bar-Ziv J, Dagan R. Nasopharyngeal Pneumococcal Carriage During Childhood Community-Acquired Alveolar Pneumonia: Relationship Between Specific Serotypes and Coinfecting Viruses. *J Infect Dis* 2017; 215: 1111-1116.

40. Habibi MS, Thwaites RS, Chang M, Jozwik A, Paras A, Kirsebom F, Varese A, Owen A, Cuthbertson L, James P, Tunstall T, Nickle D, Hansel TT, Moffatt MF, Johansson C, Chiu C, Openshaw PJM. Neutrophilic inflammation in the respiratory mucosa predisposes to RSV infection. *Science* 2020; 370.

41. DeVincenzo J, Lambkin-Williams R, Wilkinson T, Cehelsky J, Nochur S, Walsh E, Meyers R, Gollob J, Vaishnaw A. A randomized, double-blind, placebo-controlled study of an RNAi-based therapy directed against respiratory syncytial virus. *Proc Natl Acad Sci U S A* 2010; 107: 8800-8805.

42. Zaas AK, Chen M, Varkey J, Veldman T, Hero AO, 3rd, Lucas J, Huang Y, Turner R, Gilbert A, Lambkin-Williams R, Øien NC, Nicholson B, Kingsmore S, Carin L, Woods CW, Ginsburg GS. Gene expression signatures diagnose influenza and other symptomatic respiratory viral infections in humans. *Cell Host Microbe* 2009; 6: 207-217.

43. MHRA. Common issues identified during clinical trial applications. 2018 09/02/2023]. Available from: https://www.gov.uk/government/publications/common-issues-identified-during-clinical-trial-applications/common-issues-clinical.

44. NHS. Update on guidance for clinically extremely vulnerable individuals and actions for acute trusts. 2023 11/03/2023]. Available from: https://www.england.nhs.uk/coronavirus/documents/c0830-update-on-guidance-for-clinically-extremely-vulnerable-individuals-and-actions-for-acute-trusts/.

45. UKRI. Human tissue and biological samples for use in research. 2019 09/02/2023]. Available from: https://www.ukri.org/publications/human-tissue-and-biological-samples-for-use-in-research/.

46. Williams CM, Pan D, Decker J, Wisniewska A, Fletcher E, Sze S, Assadi S, Haigh R, Abdulwhhab M, Bird P, Holmes CW, Al-Taie A, Saleem B, Pan J, Garton NJ, Pareek M, Barer MR. Exhaled SARS-CoV-2 quantified by face-mask sampling in hospitalised patients with COVID-19. *J Infect* 2021; 82: 253-259.

47. German EL, Solórzano C, Sunny S, Dunne F, Gritzfeld JF, Mitsi E, Nikolaou E, Hyder-Wright AD, Collins AM, Gordon SB, Ferreira DM. Protective effect of PCV vaccine against experimental pneumococcal challenge in adults is primarily mediated by controlling colonisation density. *Vaccine* 2019; 37: 3953-3956.

48. Gritzfeld JF, Wright AD, Collins AM, Pennington SH, Wright AK, Kadioglu A, Ferreira DM, Gordon SB. Experimental human pneumococcal carriage. *J Vis Exp* 2013.

49. Jackson GG, Dowling HF, Spiesman IG, Boand AV. Transmission of the common cold to volunteers under controlled conditions. I. The common cold as a clinical entity. *AMA Arch Intern Med* 1958; 101: 267-278.

50. Talbot TR, Poehling KA, Hartert TV, Arbogast PG, Halasa NB, Edwards KM, Schaffner W, Craig AS, Griffin MR. Seasonality of invasive pneumococcal disease: Temporal relation to documented influenza and respiratory syncytial viral circulation. *The American Journal of Medicine* 2005; 118: 285-291.

Annex 1: Example diary card

Example of fields for 21-day diary card: Part A: upper respiratory symptoms score

	Sneezing	Headache	Malaise	Fever / chills	Nasal discharge	Nasal obstruction	Sore throat	Cough	Total score
Day 0									
Day 1									
Day 2									
Day 3									
Day 4									
Day 5									
Day 6									
Day 7									
Day 8									
Day 9									
Day 10									
Day 11									
Day 12									
Day 13									
Day 14									
Day 15									
Day 16									
Day 17									
Day 18									
Day 19									
Day 20									
Day 21									

Upper respiratory clinical symptom score. Scores are documented as 0 = absent, 1 = mild, 2 = moderate, 3 = severe, and 4 = emergency department visit or hospitalisation

Definition of a clinical cold

A clinical cold is diagnosed if two or more of the following are present:

- A cumulative clinical symptom score of 14 or greater over a 6 day period
- Nasal discharge is present on three or more days over the six-day period post viral inoculation
- A subjective impression of a cold developing. This latter criterion is used because there are a few subjects who have had a very strong subjective impression of a clinical cold but the cumulative clinical score does not reach the arbitrary cut-off level

	Cough on waking	Wheeze on waking	Daytime cough	Daytime wheeze	Daytime SOB*	Nocturnal cough, wheeze or SOB*	Coughing up phlegm	Total score
Day 0								
Day 1								
Day 2								
Day 3								
Day 4								
Day 5								
Day 6								
Day 7								
Day 8								
Day 9								
Day 10								
Day 11								
Day 12								
Day 13								
Day 14								
Day 15								
Day 16								
Day 17								
Day 18								
Day 19								
Day 20								
Day 21								

Lower respiratory clinical symptom score. Scores are documented as 0 = absent, 1 = mild, 2 = moderate, 3 = severe, and 4 = emergency department visit or hospitalisation SOB: Shortness of breath

Annex 2: Grading the severity of self-reported Adverse Events (challenge period)

Adverse event	Grade	Definition (in degrees Celsius)
Temperature	0	< 37.6
	1	37.6 – 38.0
	2	38.1 – 39.0

	3	> 39.0

Annex 3: Grading the severity of visit-observed Adverse Events

Observation	Grade 1	Grade 2	Grade 3	Grade 4
Oral temperature (̊C)	37.6 – 38.0	38.1 – 39.0	> 39.0	A&E visit or hospitalisation for hyperpyrexia
Tachycardia (beats/min)	101-115	116-130	>130	A&E visit or hospitalisation for arrhythmia
Bradycardia (beats/min)	50-54	45-49	<45	A&E visit or hospitalisation for arrhythmia
Systolic hyper-tension (mmHg)	141-150	151-155	>155	A&E visit or hospitalization for malignant hypertension
Diastolic hyper-tension (mmHg)	91-95	96-100	>100	A&E visit or hospitalization for malignant hypertension
Systolic hypo-tension (mmHg)	85-89	80-84	<80	A&E visit or hospitalization for hypotensive shock

Annex 4: Grading the severity of laboratory Adverse Events

Parameter	Grade 1	Grade 2	Grade 3	Grade 4*
**Haemoglobin: decrease from baseline value (g/l)**	10 - 15	16-20	21-50	>50
**White cell count: elevated (10^9/L)**	11.01 – 15.00	15.01 – 20.00	20.01 – 25.00	>25
**White cell count: depressed (10^9/L)**	2.50 – 3.50	1.50 – 2.49	1.00 – 1.49	<1.0
**Neutrophil count (10^9/L)**	1.5-2.0	1.0-1.4	0.5-0.9	<0.5
**Platelets (10^9/L)**	125-140	100-124	25-99	<25
**Sodium: hyponatraemia (mmol/L)**	132–134	130–131	125–129	<125
**Sodium: hypernatraemia (mmol/L)**	146	147	148–150	>150
**Potassium: hyperkalaemia (mmol/L)**	5.4 – 5.5	5.6 – 5.7	5.8 – 5.9	>5.9
**Potassium: hypokalaemia (mmol/L)**	3.3–3.4	3.1–3.2	3.0	<3.0
**Urea (mmol/L)**	8.2–8.9	9.0–11	>11	RRT
**Creatinine (µmol/L)**	132-150	151-176	177-221	>221 or RRT

**ALT and/or AST (IU/L)**	1.1–2.5 x ULN	>2.6–5.0 x ULN	5.1-10 x ULN	>10 x ULN
**Bilirubin, with increase in LFTs (µmol/L)**	1.1–1.25 x ULN	1.26–1.5 x ULN	1.51–1.75 x ULN	>1.75 x ULN
**Bilirubin, with normal LFTs (µmol/L)**	1.1–1.5 x ULN	1.6–2.0 x ULN	2.1–3.0 x ULN	>3.0 x ULN
**Alkaline phosphatase (IU/L)**	1.1–2.0 x ULN	2.1–3.0 x ULN	3.1–10 x ULN	>10 x ULN
**Albumin: hypoalbuminaemia (g/L)**	28–31	25–27	<25	Not applicable
**C-reactive protein**	>10-30	31-100	101-200	>200

Grade 4* Potentially life threatening

Normal ranges may vary between sites and gradings may be adapted between sites

Annex 5: Amendment History

Amendment No.	Protocol Version No.	Date issued	Author(s) of changes	Details of Changes made
n/a	**1.0**		Daniela Ferreira, Maheshi Ramasamy, Ben Morton, Grace Li, Carla Solórzano-Gonzalez	Document created
n/a	1.1	09 October 2023	Daniela Ferreira, Maheshi Ramasamy, Ben Morton, Grace Li, Carla Solorzano-Gonzalez	**Changes in the protocol on the basis of provisional opinion**
Non-Substantial 01 – NSA01	1.2	19 October 2023	Daniela Ferreira, Maheshi Ramasamy, Ben Morton, Grace Li, Carla Solórzano-Gonzalez	**Changes in the protocol:**<br>- Correcting some typos/misspellings and clarification for consistency throughout the text and with other approved documents
Non-Substantial 02 – NSA02	1.3	22 November 2023	Maheshi Ramasamy, Sanjita Brito-Mutunayagam, Carla Solórzano-Gonzalez	**Changes in the protocol:**<br>Section 8.3:<br>-Clarification of exclusion criteria of public transport as this only applies for phase B/C after participants are challenged with RSV.

Non-Substantial 03 – NSA03	1.4	05 December 2023	Maheshi Ramasamy, Sanjita Brito-Mutunayagam, Carla Solórzano-Gonzalez	**Changes in the protocol:**<br>Section 9.7:<br>- Increase of screening window to -60 days<br>-Clarification of obtention of repeated samples<br>Change of PIS to account for extension of screening period and split of screening visits. Re-consent not required.
Substantial Amendment 01 –SA01	2.0	20 February 2024	Maheshi Ramasamy, Sanjita Brito-Mutunayagam, Carla Solórzano-Gonzalez, Ben Morton, Kiarash Tanha	**Changes in the protocol:**<br>**Section 1:**<br>-Addition of Co-Investigator<br><br>**Table 1:**<br>-Secondary objective outcome measure: We will measure the absence of SAEs/AESIs relating to inoculation throughout the study and not only during phase A.<br><br>**Section 8.2 and 8.3:**<br>-Inclusion/Exclusion criteria updated<br><br>**Section 9.5:**<br>-Clarification on randomisation replacements and gender stratification<br><br>**Table 2A:**<br>-Extension of visit 8 window<br>-Additional pregnancy test included for visit 4<br>-Addition of TOPS if required per local policy<br><br>**Section 9.10:**<br>-Specific volumes of RSV to be instilled per nostril were removed.<br><br>**Section 12.3, 12.4.1 and 12.4.2:**<br>-Clarification of causality assessment of AEs

				**Section 12.6:**<br>-AESI list updated<br><br>**Section 12.9 and 15.3:**<br>-Clarification on the timelines and content of the DSMC reports<br><br>**Section 13, 13.5 and 13.6:**<br>-Clarification of statistical methods and timepoints for primary outcome analysis<br><br>**Section 13.9**<br>- Clarification of effective sample size for phase B/C.<br><br>**Annex 1:**<br>-Addition of baseline measurement (D0) and coughing up phlegm<br><br>**Annex 4:**<br>-Update of white cell count and potassium ranges.<br><br>**Changes in PIS**<br>-Update with exclusion criteria<br>-Update of the approximate duration of study visits<br>-Clarification of number of study visits<br>-Clarification of review of symptom diary review
Non-Substantial 04- NSA04	2.1	24 May 2024	Maheshi Ramasamy, Sanjita Brito-Mutunayagam, Carla Solorzano-Gonzalez, Daniela Ferreira	**Changes in the protocol:**<br>**Section 11.2:**<br>-Clarification of household contact swab management<br><br>**Change in Household Contact Screening invitation letter:**<br>-Clarification of household contact swab management

Substantial Amendment 02 – SA02	3.0	17 June 2024	Maheshi Ramasamy, Daniela Ferreira, Elena Mitsi, Nile Verleur, Carla Solórzano-Gonzalez, Seun Ajayi.<br><br>ENT Surgeons: Matthew Lawson-Coates, Samuel Leong, Aby Lamyman	**Changes in the protocol:**<br><br>**Section 1:**<br>-Addition of Co-Investigators and collaborator<br><br>Section 8.3 and 8.4:<br>-Exclusion and Temporary Exclusion criteria updated based on nasal biopsy procedure<br><br>**Section 9.13**<br><br>**-** Inclusion of nasal biopsies for participants who volunteer for this additional sampling.<br><br>**Table 2A:**<br><br>Update of visits with nasal biopsy schedule<br><br>Inclusion of emission samples at V2 (Day 2) and V5 (Day 9)<br><br>Inclusion of PBMCs and Serum samples at V7 (D21)<br><br>Section 9.14<br><br>We have updated the information regarding sample handling<br><br>**Section 18.8**<br><br>- Update of participant benefits to reflect reimbursement for nasal biopsies<br><br>**Changes to the PIS**<br><br>-Updated with the nasal biopsy procedure.<br><br>-We have updated the text regarding completion of Phase A.<br><br>**Changes to Reimbursement Information Sheet:**<br><br>Updated with the reimbursement for nasal biopsy visit.

				**Additional documents:**<br>**Change to Study ICF:**<br>Update to describe all samples collected in the study: emission and cough sample<br>A new document was also created to provide participants information regarding the nasal biopsy procedure:<br>-Nasal biopsy PIS and ICF<br>A re-consent form was also created to ask participants for consent to obtain additional samples included in the protocol: throat swab at visits 1 and 7 and emission sample at visits 2 and 5
