# Supplementary material for: Understanding the interaction of upper respiratory tract infection with respiratory syncytial virus and Streptococcus pneumoniae using a human challenge model: a multicenter, randomized controlled study protocol
Source: PLoS One. 2025 Jul 1;20(7):e0325149. doi: 10.1371/journal.pone.0325149 (PMC12212582; doi:10.1371/journal.pone.0325149)
Supplement: S3 Table — (DOCX) [file pone.0325149.s003.docx]

| **Inclusion Criteria** |
| --- |
| - Healthy adults aged 18-55 (inclusive, at the time of consent) - Fluent spoken English – to ensure a comprehensive understanding of the research project - Capacity to provide written informed consent in English - Females of childbearing potential with a negative urine pregnancy test at screening and willing to practice adequate contraceptive measures as per UK Clinical Trial Facilitation Group during the study - Willing to provide their household contacts with the Close Contact Screening Information Letter - For Phase C, RSV neutralizing antibody titre in the lowest 10^th^ percentile of screened participants |
| **Exclusion Criteria** |
| - Research participant:   - Currently involved in another study unless observational or non-interventional. Exceptions may be applied at the discretion of the Chief Investigator to ensure no harm comes to the participants (e.g. excessive blood sampling or nasal sampling)   - Participated in a previous Spn6B EHPC study ≤3 years or an Spn3 EHPC study ≤1 year before screening - Unable to travel to outpatient clinic for visits during phase B/C after RSV challenge (for up to 10 days or 7 days if negative for RSV) without using public transport. - Unable to wear a fluid resistant surgical mask. - Vaccination**:**   - No live vaccination within four weeks prior to enrolment (defined as time of first inoculation)   - Previous pneumococcal or (investigational) RSV vaccination (including in a research study) - Allergy:   - Allergy to beta-lactam antibiotics (including penicillin and amoxicillin)   - Allergy to Lidocaine local anesthetic (for optional nasal biopsy participants only) - Medical history leading to increased risk of severe infection, illness including but not limited to:   - Asplenia or dysfunction of the spleen   - Chronic respiratory disease (e.g. asthma [requiring medication (including salbutamol inhaler) within last 12 months], COPD, bronchiectasis, and sleep apnoea)   - Chronic heart disease (e.g. angina, ischaemic heart disease, chronic heart failure) – controlled and stable hypertension may be included   - Chronic kidney disease (e.g. nephrotic syndrome, kidney transplant, requires dialysis)   - Chronic liver disease (e.g. cirrhosis, biliary atresia, hepatitis)   - Chronic neurological disease that limits mobility, bulbar or respiratory function (including stroke, Parkinson’s disease, dementia, and multiple sclerosis)   - Diabetes mellitus (including diet controlled)   - Receipt of immunosuppressive therapy such as anti-cancer chemotherapy or radiation therapy within the preceding 12 months or long-term systemic corticosteroid, Roaccutane, or disease modifying anti-rheumatoid drugs therapy (for more than 7 consecutive days within the 3 months prior to enrolment)   - Individuals with cochlear ear implants   - Individuals with major CSF leaks (e.g. following traumatic, major skull surgery, or requiring CSF shunts)   - Subjects with known or suspected immune deficiency (e.g. known IgA deficiency, immotile cilia syndrome, or Kartagener’s syndrome)   - Autoimmune disease   - History of frequent nose bleeds   - Bleeding disorders   - History of significant unexplained bleeding after a surgical or dental procedure (for optional nasal biopsy participants only) - Current medical issues   - Acute URTI in the four weeks preceding recruitment   - Any uncontrolled medical or surgical condition (e.g. mental health conditions, epilepsy, narcolepsy, or chronic pain) at the discretion of the study doctor - Any major pneumococcal illness or pneumonia requiring hospitalization in the last 10 years - Medication:   - Any medication that may affect the immune system in the last 3 months (e.g. systemic steroids [IM/IV], Roaccutane, disease modifying anti-rheumatoid drugs)   - Long-term antibiotic use   - Recipient of monoclonal antibodies for any indication within one year of period at screening   - Recipient of blood transfusion products within the last year   - Any medication that may affect the coagulation system in the last 3 months (excluding aspirin)   - Use of any medication or other product (prescription or over the counter) for symptoms of rhinitis or nasal congestion within the last 1 month - Maternal:   - Female participants who are pregnant   - Female participants who are lactating   - Female participants who intend to become pregnant during the study   - Female participants unable to take contraception measures during the study (from consent to final study visit at day 60) - Direct caring role or share living accommodation with individuals at higher risk from infection:   - Children under the age of 5 years   - Adults > 65 years old.   - Adults with chronic ill health or immunosuppression   - Adults classified as clinically extremely vulnerable by the NHS - Health-care worker - Current or ex-smoker (regular cigarettes/cigars/e-cigarette/vaping/smoking of recreational drugs) in the last 6 months - Previous significant smoking history (more than 5 cigarettes per day for 20 years or the equivalent [e.g. >5 pack years]) - Regularly drinks ≥3units/day (male) or ≥2units/day (female) - Regularly uses recreational drugs - Significant mental health disorders:   - Uncontrolled condition or previous admission to a psychiatric unit (at the discretion of the research clinician) which would impair the participants ability to safely participate in the study   - Moderate or severe depression or anxiety as classified by the Hospital Anxiety and Depression Score at screening or challenge that is deemed clinically significant by the study doctors - Overseas travel planned during 21-day period following first inoculation - Participants FBC results do not meet the required criteria on screening bloods: HB <90 g/L, total WCC <1.5x10^9^/L, total WCC <12x10^9^/L and platelets <75x10^9^/L - Any other issue which, in the opinion of the study staff, may:   - Put the participant or their contacts at risk because of participation in the study.   - Adversely affect the interpretation of the study results, or   - Impair the participant’s ability to participate in the study. |
| **Temporary exclusion criteria on day of challenge** |
| - Current acute infective illness – delay inoculation by 14 days - Recent /current URTI – delay inoculation by 4 weeks after last day of illness - Asymptomatic positive COVID-19 OR *RSV-A swab (taken on day of planned challenge) – delay inoculation by 21 days - Antimicrobial (including antiviral) use – delay inoculation by 28 days from last date of antimicrobial therapy - Nasal carriage- participants who have natural pneumococcal carriage identified at screening will be excluded– delay until carriage clearance |
| **Temporary exclusion criteria to nasal biopsy** |
| - Antibiotic use (during the study)-delay nasal biopsy for at least 1 week from last date of therapy - Dental infections- delay nasal biopsy for at least 2 weeks after last day of illness |

**S2 Table: RESPECCT inclusion and exclusion criteria including temporary exclusion criteria.**

* For clarity, a participant who has a positive RSV-A swab at day 7 following primary RSV inoculation will proceed to second inoculation with pneumococcus unless they meet individual stopping rule criteria or at investigator discretion.

Abbreviations: EHPC, experimental human pneumococcal colonisation; COPD, chronic obstructive pulmonary disease; CSF, cerebrospinal fluid; IM, intramuscular; IV, intravenous; FBC, full blood count; HB, haemoglobin; WCC, white cell count; URTI, upper respiratory tract infection
