# Supplementary material for: Understanding the interaction of upper respiratory tract infection with respiratory syncytial virus and Streptococcus pneumoniae using a human challenge model: a multicenter, randomized controlled study protocol
Source: PLoS One. 2025 Jul 1;20(7):e0325149. doi: 10.1371/journal.pone.0325149 (PMC12212582; doi:10.1371/journal.pone.0325149)
Supplement: S4 File — (PDF) [file pone.0325149.s004.pdf]

[redacted]

Oxford Vaccine Group
University of Oxford
Centre for Clinical Vaccinology and Tropical Medicine,
Churchill Hospital, Headington, Oxford OX3 7LE

[redacted]

For 21 days after your first inoculation

1. **Take your temperature with the thermometer provided in the morning for the first FIVE days after each inoculation AND if you feel feverish at any time for 21 days**
2. **Record any symptoms you are experiencing in your diary**
3. **We will monitor the completion of diaries remotely and if we see it has not been completed, we will contact you**
4. **Please provide contact details of someone that can contact you if we have not heard from you**
5. **Always keep your antibiotics and our contact details with you during the study**

[redacted]

Oxford Vaccine Group
University of Oxford
Centre for Clinical Vaccinology and Tropical Medicine,
Churchill Hospital, Headington, Oxford OX3 7LE

[redacted]

and return any unused antibiotics to our team at the end of the study

RESPECT

REspiratory syncytial virus and S. pnEumoniae Challenge Coinfection sTudy

Safety Information Leaflet

Contact out of hours:

- **Call XXXXXXX**
- **During office hours: call XXXXXX**

If you are unwell	Action
Very unwell<br><br>OR<br><br>Very concerned about your health	**Contact your GP or Emergency Department *and***<br>Inform the OVG Study team by *calling* **XXXXX** (XXXX) if unable to get through) *and*<br>**Start taking the antibiotic provided** (one tablet of Amoxicillin three times a day for 3 days) *and*<br>**Tell the doctor:**<br>• Live ***Streptococcus pneumoniae* strain 6B** was inoculated into your nose<br>• It is sensitive to penicillin<br>• To contact us if they require more information
Mild/	**Contact the Respiratory Research Team (day and night)**<br>• Fever temp >37.5˚C (if below 36˚C please recheck again)

Moderate symptoms	• Shivering<br>• Headache<br>• New rash<br>• Drowsiness<br>• Cough<br>• Earache /Eye infection<br>• Shortness of breath<br>• Loss of taste or smell

Report if unwell or early symptoms to make sure you are assessed and treated

Contact card: Keep your contact card and antibiotics with you at all times

Contact person: Please give a copy of this safety information sheet to your close contact (partner or housemate etc)

What if I am not near a phone?

If you are unwell and you are unable to contact the research team, we would advise that you start taking your antibiotics straight away. If you have any concerns, we recommend you attend your nearest GP, Walk in centre or Emergency Department.

What do I tell the doctor?

If, for any reason you attend your doctor or the hospital, you need to inform them that you have been exposed to live ***Streptococcus pneumoniae*** **SPN6B** and attempted to transmit this into your nose. These bacteria can be treated by amoxicillin as it is sensitive to these antibiotics. Please also contact us as soon as you can.

What if I have a general cough or cold?

Please contact us and we can advise you whether we would like to assess you in the clinic. In some cases, we may ask you to have a throat swab to confirm if you

[redacted]
Oxford Vaccine Group
University of Oxford
Centre for Clinical Vaccinology and Tropical Medicine,
[redacted]

have an infection, advise you to take the antibiotics or advise you to see your GP.

Safety monitoring

To ensure you are not experiencing any problems we ask you to fill in the diary daily for 21 days and to inform us at any time in the study if you are unwell for any reason. Your study team will monitor your diary daily and will advise you when the best time to complete it is.

If you are unwell:

Pneumococcus bacteria may cause infection. Although this is very unlikely, we ask you to familiarise yourself with early symptoms or signs that may indicate infection to make sure they are recognised and treated. We also want to know if you develop any symptoms suggestive of Covid-19 disease to minimise any risk of transmission. Please contact the research team who are available day and night if you have any symptoms as follows:

**If you have these symptoms Contact the research team:**	• **Fever (temp>37.5 ˚C) (if below 36 ˚C please recheck)**<br>• **Shivering**<br>• **Headache**<br>• **New rash**<br>• **Drowsiness**<br>• **Cough**<br>• **Earache/eye infection**<br>• **Loss of taste or smell**<br>• **Shortness of breath**

**If you are very unwell or concerned about your health**<br>***Caution:*** it is possible that you may be unwell for another reason not related to these bacteria.	Seek urgent care from your GP or hospital<br><br>Start taking the antibiotic (one tablet of Amoxicillin three times a day for 3 days)<br><br>Tell the doctor:<br>- You were exposed to live *Streptococcus pneumoniae* SPN6B<br>- It is sensitive to penicillin<br>- You have no history of allergy to this antibiotic.

[redacted]
Oxford Vaccine Group
University of Oxford
Centre for Clinical Vaccinology and Tropical Medicine,
Churchill Hospital, Headington, Oxford OX3 7LE
[redacted]
[redacted]

[redacted]

Oxford Vaccine Group
University of Oxford
Centre for Clinical Vaccinology and Tropical Medicine,
Churchill Hospital, Headington, Oxford OX3 7LE
[redacted]

[redacted]

RESPECCT

***RE**spiratory syncytial virus and **S**. **p**n**E**umoniae **C**hallenge **C**oinfection s**T**udy*

Respiratory precautions and self-isolation guidance – preventing transmission of Respiratory Syncytial Virus and *Streptococcus pneumoniae* 'pneumococcus' (RSV/P)

In order to prevent you transmitting RSV/P to anyone around you, it is very important that you follow some basic guidelines regarding everyday hygiene. If these are followed it is very unlikely that you would transmit RSV/P. The advice below relates to hygiene to prevent transmission. After the RSV inoculation, you will be required to self-isolate for 7-10 days. Advice on this is included at the end of this document on page 2.

Transmission

Respiratory Syncytial Virus (RSV) is typically spread by large droplets through direct contact with infectious respiratory secretions. The virus can survive on surfaces or objects for about 4 to 7 hours (UK Health Security Agency, 2023).

The germs *Streptococcus pneumoniae*, or 'pneumococcus' are usually breathed in. People often have small amounts of germs in their nose and throat that can be passed on through coughing, sneezing, touching objects and spreading contact (NHS, 2019).

Hand washing

Hand washing is the most important thing to do to prevent the spread of these germs. You should thoroughly wash your hands with liquid soap in warm running water for at least 20 seconds, and dry carefully. If paper towels are not available, use your own personal towel to keep separately from others in your household (e.g., keep it in your room for your own use). You should avoid sharing all towels with other people. Please refer to page 2.

Coughs and Sneezes

When coughing and sneezing tiny droplets launch fluid-containing germs into the air or onto surrounding objects. These germs can be transmitted if someone else breathes them in, or touches the germs covering the object, and then touches their own nose or mouth. Cover your coughs and

sneezes with a tissue or your upper shirt sleeve, **not your hands**. See ‘sneezing and coughing manners’ on page 3.

Self-isolation after RSV inoculation

After inoculation with the RSV germ, you are required to self-isolate at home for 7-10 days. On day 7 we will do a test for RSV in the research clinic. If this is negative you will be able to leave self-isolation early, but if it is positive you must continue to self-isolate for a further 3 days. This is to protect other people from catching the virus.

This self-isolation is very similar to that during the recent COVID-19 pandemic. You will be given at least 48 hours’ notice prior to the RSV inoculation so that you can prepare properly, for example by buying food and over-the-counter medication like paracetamol.

You **must stay at home** during your self-isolation period, other than for your research clinic appointments (or if a medical emergency should unexpectedly occur). You should not go to work, university, shops, other people’s houses, public areas, or use public transport. You must not have visitors to the house. You can use any private outdoor space like a garden or a balcony if you have one, but you should wear a surgical face mask and keep at least 2 metres away if other members of the public or household are present in this space.

Whilst at home you should sleep in a separate room to anyone else you live with and maintain social distancing from others in your household. This means you should always stay at least 2 metres away, wear surgical face masks and open windows in communal spaces in the presence of other household members. If you share a kitchen or bathroom with others, then plan to use those rooms at different times and clean them thoroughly after use with household cleaning products. You should eat in a separate room if you live with others and shouldn’t share cups or eating utensils. You should avoid close contact like kissing or shaking hands. Please put tissues and facemasks in a bin straight after using them.

The safest way of travelling to your research clinic appointments is on foot or cycling (if you feel fit enough) whilst wearing a surgical facemask. You are also allowed to drive yourself whilst wearing a facemask but be mindful of staying at least 2 metres away from others in carparks. You can take a

private taxi but please inform the driver before you get in the car; offer a facemask; and open windows in the car.

Self-isolation can be difficult. We will check in with you to see how the study may affect your mood. A few recommendations to help with your mood include regular contact with friends and family, maintaining a sense of routine, keeping a healthy diet and varying activities such as podcasts and reading. (NHS Kent and Medway 2021)

If you wish to discuss any of this document, then please speak to the study team.

Duration of the entire procedure: 40-60 seconds

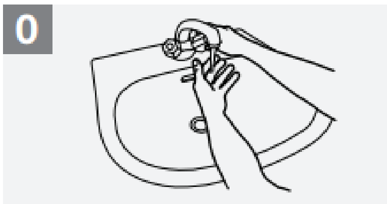

Wet hands with water;

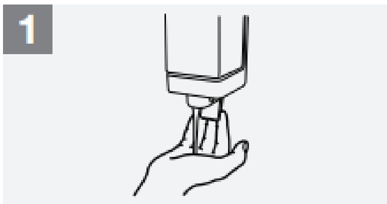

Apply enough soap to cover all hand surfaces;

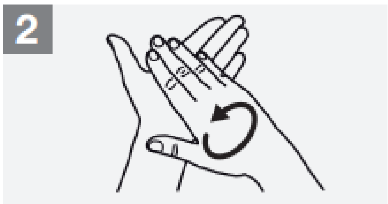

Rub hands palm to palm;

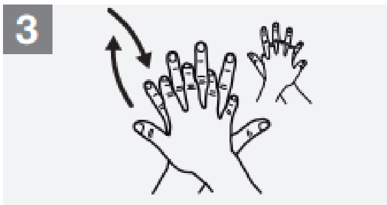

Right palm over left dorsum with interlaced fingers and vice versa;

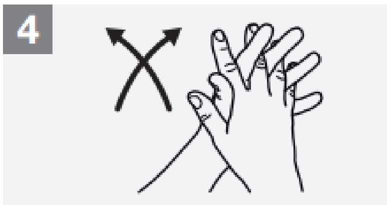

Palm to palm with fingers interlaced;

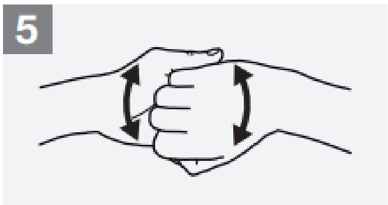

Backs of fingers to opposing palms with fingers interlocked;

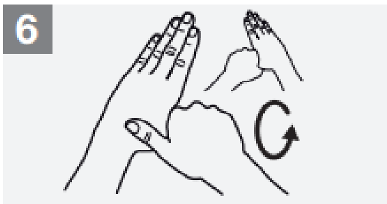

Rotational rubbing of left thumb clasped in right palm and vice versa;

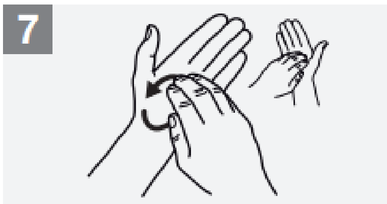

Rotational rubbing, backwards and forwards with clasped fingers of right hand in left palm and vice versa;

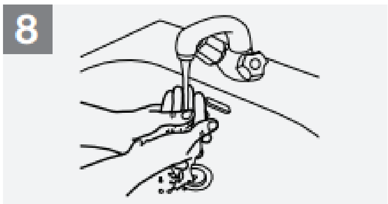

Rinse hands with water;

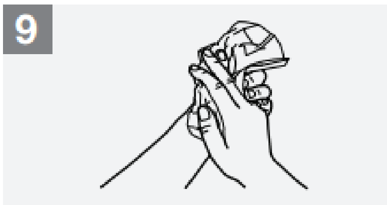

Dry hands thoroughly with a single use towel;

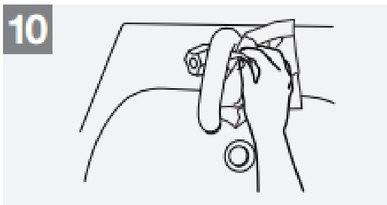

Use towel to turn off faucet;

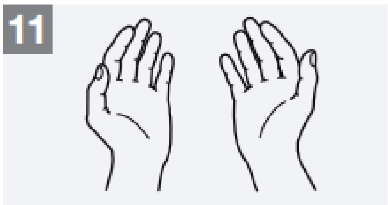

Your hands are now safe.

World Health Organization | Patient Safety — A World Alliance for Safer Health Care | SAVE LIVES Clean Your Hands

All reasonable precautions have been taken by the World Health Organization to verify the information contained in this document. However, the published material is being distributed without warranty of any kind, either expressed or implied. The responsibility for the interpretation and use of the material lies with the reader. In no event shall the World Health Organization be liable for damages arising from its use.
WHO acknowledges the Hôpitaux Universitaires de Genève (HUG), in particular the members of the Infection Control Programme, for their active participation in developing this material.

May 2009

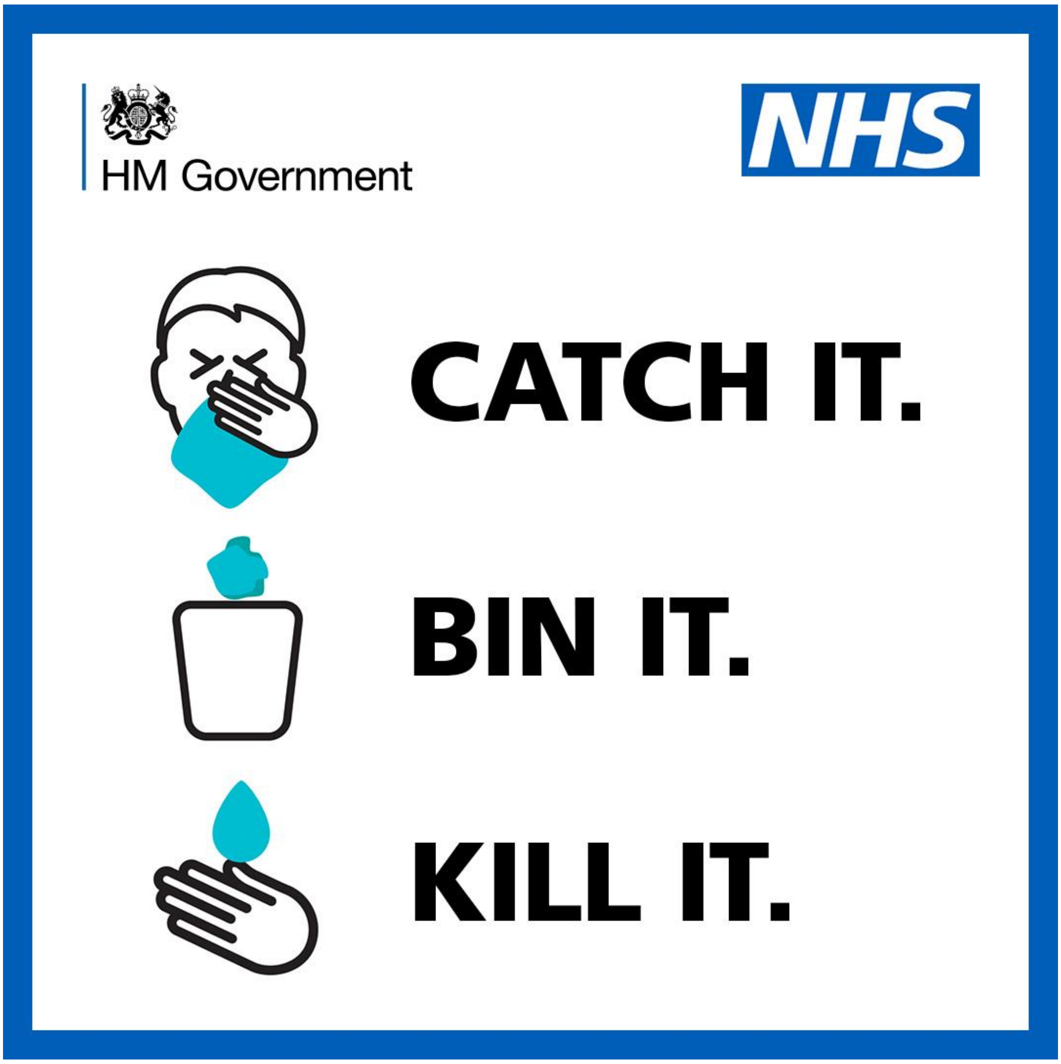
HM Government
NHS
CATCH IT.
BIN IT.
KILL IT.

“Coughing Manners”

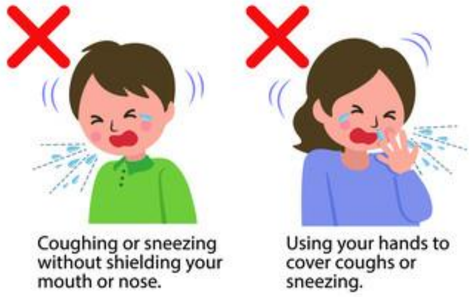

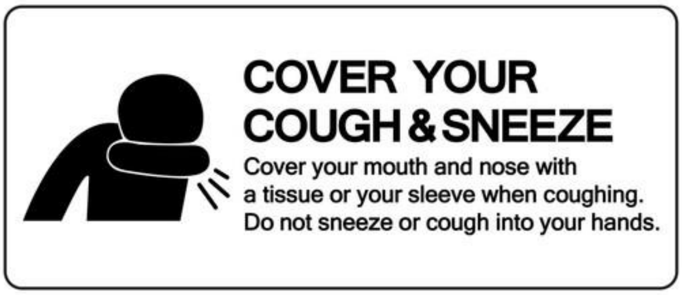

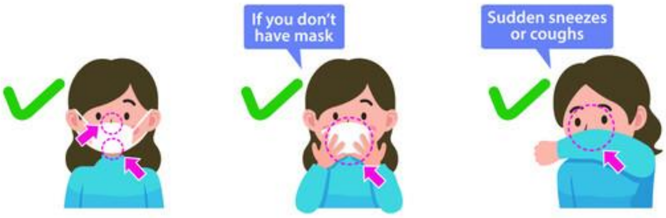

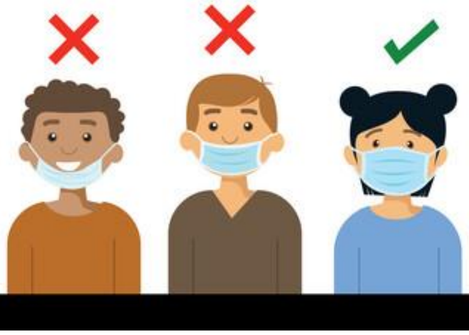

[redacted]

[redacted]

Oxford Vaccine Group

University of Oxford

Centre for Clinical Vaccinology and Tropical Medicine,

Churchill Hospital, Headington, Oxford OX3 7LE

[redacted]

RESPECCT

RE*spiratory syncytial virus and* ***S. pnE****umoniae*

C*hallenge* ***C****oinfection s****T****udy*

CHALLENGE DIARY CARD

Participant Number: __ __ __ __

Participant Initials: __ __

Challenge date 1: __ __/__ __ __/__ __ __ __

Challenge time: __ __: __ __ hours

Challenge date 2: __ __/__ __ __/__ __ __ __

Challenge time: __ __: __ __ hours

Reminder: bring this diary with you to every visit

WHAT IS THIS DIARY FOR?

This is a **paper backup diary.** We would like you to complete the e-diary to collect information about any symptoms you have after your ***Respiratory Syncytial Virus and Streptococcus pneumoniae*** 'pneumococcus' ***(RSV-P)*** challenges, which is accessed on the internet at:

https://apps.ovg.ox.ac.uk/redcap/XXXXXXX

It is important that you complete the diary online as the study doctor will be reviewing this regularly. If you are, for any reason, unable to access the internet, this paper diary can be used as a backup to record your symptoms. Please make sure you have collected the self-samples required for today, if you have any questions please speak to study team

Completion of the diary will help the study team monitor you closely throughout the study, to assess for the development of RSV-P infection and look after you appropriately. The risks associated with RSV-P challenge are greatly minimised by attending the study visits, completing the e-diary and maintaining close contact with the study team.

We will review your diary with you at each visit. Thank you very much for assisting us with this study.

Reminder: bring this diary with you to every visit

If you are hospitalised for any reason *or* if you are concerned about your health, please contact the study team immediately

Our contact details are:

Website: www.ovg.ox.ac.uk

[redacted]

24-hour contact number:

XXXXXXXXXXX

Temperature

Please check your temperature, with the thermometer provided, in the **MORNING for the first FIVE days** after each inoculation at about the same time each day (e.g. at 7am), **AND** if you feel feverish at any time for 21 days, please record this on page 14.

Please check your temperature by placing the thermometer under your tongue until it beeps. Please immediately record the reading, using one decimal number (e.g. 37.5°C). Avoid checking your temperature for 30 minutes after eating or drinking. If the temperature reading is below 35.5°C, please check it again to be sure your thermometer is working properly.

Participants are asked to contact the team daily or the team will make contact for the first five days after each inoculation with their temperature recording and any symptoms before 12:00pm/noon. This is as a safety precaution in the event of developing pneumococcal infection.

You **must complete** the Upper and Lower respiratory clinical symptom tables (green and blue tables) for the next 21 days. Please ensure you complete this every day giving a score from 0 to 4.

Please notify the study team immediately if your temperature is 38°C or above.

Medication

If you take any medication agreed by the study team, please record on page 18.

Regular medication that you normally take can be continued and does not need to be recorded.

Symptoms of infection

You may develop symptoms of respiratory infection following the challenge; please complete the ‘symptoms’ tables on pages 6-12 Please record any additional symptoms you experience on the pink table on page 16. Please complete each day on the e-Diary (this paper copy is just a back-up).

Please contact the study team immediately if you are concerned about any of the symptoms you have developed.

If you have not experienced a symptom please circle "0" in the diary page for the relevant day. If you have experienced a symptom please circle the number indicating the severity of the symptom using the scale.

Table 1. Upper respiratory clinical symptom score scale

Symptom	Severity				
	0	1	2	3	4
Sneezing	No sneezing present	Present but no interference with activity	Some interference with activity	Significant; prevents daily activity	Emergency department visit or hospitalisation
Headache	No headache present	Present but no interference with activity	Some interference with activity	Significant; any use of codeine phosphate or prevents daily activity	Emergency department visit or hospitalisation
Malaise	No malaise	Present but no interference with activity	Some interference with activity	Significant; prevents daily activity	Emergency department visit or hospitalisation
Fever / chills	No fever	Present but no interference with activity	Some interference with activity	Significant; prevents daily activity	Emergency department visit or hospitalisation
Nasal discharge	No nasal discharge	Present but no interference with activity	Some interference with activity	Significant; prevents daily activity	Emergency department visit or hospitalisation
Nasal obstruction	No nasal congestion present	Present but no interference with activity	Some interference with activity	Significant; prevents daily activity	Emergency department visit or hospitalisation
Sore throat	No sore throat present	Present but no interference with activity	Some interference with activity	Significant; any use of codeine phosphate or prevents daily activity	Emergency department visit or hospitalisation
Cough	No cough present	Present but no interference with activity	Some interference with activity	Significant; prevents daily activity	Emergency department visit or hospitalisation

If you have not experienced a symptom please circle "0" in the diary page for the relevant day. If you have experienced a symptom please circle the number indicating the severity of the symptom using the scale.

Table 2. Lower respiratory clinical symptom score scale

Symptom	Severity				
	0	1	2	3	4
Cough on waking	No cough present	Present but no interference with activity	Some interference with activity	Significant; prevents daily activity	Emergency department visit or hospitalisation
Wheeze on waking	No wheeze present	Present but no interference with activity	Some interference with activity	Significant; prevents daily activity	Emergency department visit or hospitalisation
Daytime cough	No cough present	Present but no interference with activity	Some interference with activity	Significant; prevents daily activity	Emergency department visit or hospitalisation
Daytime wheeze	No wheeze present	Present but no interference with activity	Some interference with activity	Significant; prevents daily activity	Emergency department visit or hospitalisation
Daytime shortness of breath	No shortness of breath present	Present but no interference with activity	Some interference with activity	Significant; prevents daily activity	Emergency department visit or hospitalisation
Nocturnal cough, wheeze or shortness of breath	No shortness of breath present	Present but no interference with activity	Some interference with activity	Significant; prevents daily activity	Emergency department visit or hospitalisation
Coughing up phlegm	No coughing up phlegm present	Present but no interference with activity	Some interference with activity	Significant; prevents daily activity	Emergency department visit or hospitalisation

If you have not experienced a symptom please write “0” in the diary box for the relevant day.

If you have experienced a symptom please write the number indicating the severity of the symptom using the scale:

Upper respiratory clinical symptoms (green table).

Scores are documented as 0 = absent, 1 = mild, 2 = moderate, 3 = severe,and 4=emergency deparment visit or hospitalisation. See the severity descriptions on Table 1, page 6

Take your temperature every day for 5 days after each inoculation before 12pm / noon. If you have a temperature over 38 °C please report result to the study doctor as soon as possible. If you feel feverish keep measuring your temperature at any time for 21 days and record this on page 14.

**Symptom**	**Day 0**	**Day 1**	**Day 2**	**Day 3**	**Day 4**	**Day 5**	**Day 6**	**Day 7**	**Day 8**	**Day 9**	**Day 10**	**Day 11**	**Day 12**
Temperature	°C	°C	°C	°C	°C	°C			°C	°C	°C	°C	°C
Time temperature taken (HH:MM)													
Sneezing													

Headache													
Malaise													
Fever / chills													
Nasal discharge													
Nasal obstruction													
Sore throat													
Cough													
Total score													

Day 13 to Day 21 continued on next page…

**Symptom**	**Day 13**	**Day 14**	**Day 15**	**Day 16**	**Day 17**	**Day 18**	**Day 19**	**Day 20**	**Day 21**
Temperature									
Time temperature taken (HH:MM)									
Sneezing									
Headache									
Malaise									
Fever / chills									
Nasal discharge									
Nasal obstruction									
Sore throat									

Cough									
Total score									

If you have not experienced a symptom please write "0" in the diary box for the relevant day.

If you have experienced a symptom please write the number indicating the severity of the symptom using the scale:

Lower respiratory clinical symptom score (blue table)

Scores are documented as 0 = absent, 1 = mild, 2 = moderate, 3 = severe, and 4=emergency department visit or hospitalisation. See the severity descriptions on Table 2, page 7

**Symptom**	**Day 0**	**Day 1**	**Day 2**	**Day 3**	**Day 4**	**Day 5**	**Day 6**	**Day 7**	**Day 8**	**Day 9**	**Day 10**	**Day 11**	**Day 12**	**Day 13**	**Day 14**
Cough on waking															
Wheeze on waking															
Daytime cough															

Daytime wheeze															
Daytime shortness of breath															
Nocturnal cough, wheeze or shortness of breath															
Coughing up phlegm															
Total score															

Lower respiratory clinical symptom score diary from Day 15 to Day 21 continued on the next page…

Symptom	Day 15	Day 16	Day 17	Day 18	Day 19	Day 20	Day 21
Cough on waking							
Wheeze on waking							
Daytime cough							
Daytime wheeze							
Daytime shortness of breath							
Nocturnal cough, wheeze or shortness of breath							
Coughing up phlegm							
Total score							

Temperature Log

If you feel feverish at any other time please check your temperature and record below:

Date								
Day (post challenge)								
Time								
Oral (mouth) temperature	ºC	ºC	ºC	ºC	ºC	ºC	ºC	ºC

Date								
Day (post challenge)								
Time								
Oral (mouth) temperature	ºC	ºC	ºC	ºC	ºC	ºC	ºC	ºC

Additional Symptoms

You may experience other symptoms such as feeling hot or cold, sweating or shivering. If you feel unable to manage any of the symptoms you are experiencing, please contact the study team immediately.

Please record any additional symptoms on the following tables. Please include any illness/injury even if you think it is not related to the study. Please include the start date, start time, finish date, finish time (or state ongoing if not resolved by day 21, and severity of symptoms (see below scale).

Please grade any symptoms using the following scale:

- 0 No symptoms
- 1 Mild – easily tolerated with no limitation on normal activity
- 2 Moderate – some limitation of daily activity
- 3 Severe – unable to perform normal daily activity
- 4 Emergency department or hospital visit required

**Start Date/time**	**Symptom details**	**Severity** (please circle)	**Finish date/time** (state ongoing if continuing)
__/__/__ __:__		0 1 2 3 4	__/__/__ __:__
__/__/__ __:__		0 1 2 3 4	__/__/__ __:__
__/__/__ __:__		0 1 2 3 4	__/__/__ __:__

__/__/__<br>__:__		0 1 2 3 4	__/__/__<br>__:__

Medications

Please record any medication taken in addition to any regular medication that you take. Please do not take any medication unless agreed by the study team.

Date	Time (24 hr clock)	Medication	Dose	Reason for taking

Please use the lines below to report additional information not reported in the previous tables because of the lack of space or any other comment you consider relevant.

____

____

____

____

____

____

____

____

INVESTIGATOR'S COMMENTS ON DIARY DATA CORRECTIONS:

____

____

____

____

____

____

I declare that any information recorded in this diary has been accurately reviewed and discussed with the participant.

After correction of obvious mistakes all data have been transcribed faithfully into the e-diary.

____ ___________________

Date Investigator's Signature

[redacted]

Oxford Vaccine Group

University of Oxford

Centre for Clinical Vaccinology and Tropical Medicine,

Churchill Hospital, Headington, Oxford OX3 7LE

[redacted]

WHO SHALL I CONTACT DURING THE STUDY?

If you have any questions during the study, or if you want to contact the study team for any reason please telephone:

Website: www.ovg.ox.ac.uk

[redacted]

Out of office hours please contact the 24-hour contact number:

XXXXXXX
