# Supplementary material for: Understanding the interaction of upper respiratory tract infection with respiratory syncytial virus and Streptococcus pneumoniae using a human challenge model: a multicenter, randomized controlled study protocol
Source: PLoS One. 2025 Jul 1;20(7):e0325149. doi: 10.1371/journal.pone.0325149 (PMC12212582; doi:10.1371/journal.pone.0325149)
Supplement: S5 Table — (DOCX) [file pone.0325149.s005.docx]

| **Objectives** | **Outcome measures** |
| --- | --- |
| **Primary study objectives** |  |
| To determine if primary RSV-A challenge increases the risk of secondary Spn6B carriage using classical culture | Colonization rates of Spn6B determined by classical culture |
| **Secondary study objectives** | |
| To confirm co-infection model safety for participants | Absence of SAEs/AESIs relating to inoculation throughout the study |
| To determine if primary RSV-A challenge increases the risk of secondary Spn6B carriage using molecular methods | Colonization rates of Spn6B determined by RT-qPCR |
| To determine if primary RSV-A infection increases the risk of secondary Spn6B carriage | Colonization rates of Spn6B determined by classical culture and molecular methods |
| To determine if primary RSV-A challenge or infection alters the density of secondary Spn6B carriage | Density of Spn6B carriage by classical culture and molecular methods |
| To determine if primary RSV-A challenge or infection alters the duration of secondary Spn6B carriage | Quantification of Spn6B determined longitudinally by classical culture and RT-qPCR |
| To determine if primary Spn6B challenge alters the risk of secondary RSV-A infection | RSV-A attack rates determined by RT-qPCR |
| To determine if primary Spn6B carriage alters the risk of secondary RSV-A infection | RSV-A attack rates determined by RT-qPCR |
| To determine if primary Spn6B challenge or carriage alters the viral load of secondary RSV-A infection (AUC of density over time) | RSV-A viral load determined by RT-qPCR |
| To determine if primary Spn6B challenge or carriage alters the duration of secondary RSV-A infection | Duration of RSV-A detection and quantification determined by RT-qPCR |
| To determine if co-infection with RSV-A and Spn6B alter URTI and LRTI symptoms | Number of URTI and LRTI symptoms per participant after secondary challenge |
| Longitudinal assessment of viral and bacterial shedding from the nose in both arms of the study | Shedding of pathogens from the nose and throat will be assessed by coughing plate, hand swab after nose touching and facemasks assessments  RSV-A viral load and Spn6B density by RT-qPCR |
| To determine if primary Spn6B carriage increases the proportion of participants from whom live RSV-A can be isolated from their hands | Shedding of pathogens from the nose and throat will be assessed by coughing plate, hand swab after nose touching and facemasks assessments  RSV-A viral load and Spn6B density by RT-qPCR |
| To determine if primary or secondary RSV-A infection increases the proportion of participants from whom live Spn6B can be isolated from their hands | Shedding of pathogens from the nose and throat will be assessed by coughing plate, hand swab after nose touching and facemasks assessments  RSV-A viral load and Spn6B density by RT-qPCR |
| To determine if Spn6B carriage density correlates with bacterial shedding | Shedding of pathogens from the nose and throat will be assessed by coughing plate, hand swab after nose touching and facemasks assessments  RSV-A viral load and Spn6B density by RT-qPCR |
| To determine if RSV-A nasal and throat viral load correlates with viral shedding | Shedding of pathogens from the nose and throat will be assessed by coughing plate, hand swab after nose touching and facemasks assessments  RSV-A viral load and Spn6B density by RT-qPCR |
| To determine if the outpatient RSV-A challenge model is associated with any increased risk of infection in household contacts | Nasopharyngeal or nasal swabs of household contacts during two weeks following RSV-A challenge visit |
| **Exploratory study objectives:** | |
| To identify if primary Spn6B carriage alters immune responses to secondary RSV-A infection (innate and adaptive) | Assessment of immune responses including nasal cytokines and cell populations as well as RSV-specific antibodies and cellular immunity before and after each pathogen challenge. |
| To identify how primary RSV-A challenge or infection alters immune responses to secondary Spn6B carriage | Assessment of immune responses including nasal cytokines and cell populations as well as Spn6B-specific antibodies and cellular immunity before and after each pathogen challenge. |
| Nasal cells gene expression alterations and their visualization (spatial location) within nasal tissue | Longitudinal analysis of the spatial microenvironment and transcriptomics of nasal tissue samples |
| Characterize transcriptional changes in immune cells (via single cell and bulk RNA sequencing) in response to RSV-A and Spn6B challenge/infection/carriage. | RNAseq analysis to determine gene induction and regulation to identify gene signatures (cell specific where possible) that correlate with susceptibility to infection, as well as alterations in immune responses and symptoms during co-infection |
| Assess pneumococcal transcriptional alterations in response to RSV-A co-infection | Determination of gene induction and regulation to identify bacterial response to viral co-infection |

**S3 Table. Primary, secondary, and exploratory objectives and outcomes of the RESPECCT study.**

Abbreviations: RSV-A, Respiratory Syncytial Virus-A; Spn6B, *Streptococcus pneumoniae* serotype 6B; AE, adverse event; AUC, area under the curve; AESI, adverse event of special interest; SAE, significant adverse event; RT-qPCR, reverse transcription quantitative polymerase chain reaction; URTI, upper respiratory tract infection; LRTI, lower respiratory tract infection; RNAseq, RNA sequencing.
