# Supplementary material for: Understanding the interaction of upper respiratory tract infection with respiratory syncytial virus and Streptococcus pneumoniae using a human challenge model: a multicenter, randomized controlled study protocol
Source: PLoS One. 2025 Jul 1;20(7):e0325149. doi: 10.1371/journal.pone.0325149 (PMC12212582; doi:10.1371/journal.pone.0325149)
Supplement: S6 Table — (DOCX) [file pone.0325149.s006.docx]

| **Observation** | **Grade 1** | **Grade 2** | **Grade 3** | **Grade 4** |
| --- | --- | --- | --- | --- |
| Oral temperature  (℃) | 37.6 – 38.0 | 38.1 – 39.0 | > 39.0 | A&E visit or hospitalization for hyperpyrexia |
| Tachycardia (beats/min) | 101-115 | 116-130 | >130 | A&E visit or hospitalization for arrhythmia |
| Bradycardia (beats/min) | 50-54 | 45-49 | <45 | A&E visit or hospitalization for arrhythmia |
| Systolic hyper-tension (mmHg) | 141-150 | 151-155 | >155 | A&E visit or hospitalization for malignant hypertension |
| Diastolic hyper-tension (mmHg) | 91-95 | 96-100 | >100 | A&E visit or hospitalization for malignant hypertension |
| Systolic hypo-tension (mmHg) | 85-89 | 80-84 | <80 | A&E visit or hospitalization for hypotensive shock |

**Table 5A: Grading of visit-observed adverse events**

Abbreviations: A&E; accident and emergency, mmHG; millimeters of mercury; ℃, Degree Celsius.

| **Parameter** | **Grade 1** | **Grade 2** | **Grade 3** | **Grade 4** |
| --- | --- | --- | --- | --- |
| Hemoglobin: decrease from baseline value (g/l) | 10 - 15 | 16-20 | 21-50 | >50 |
| White cell count: elevated (10^9^/L) | 11.01 – 15.00 | 15.01 – 20.00 | 20.01 – 25.00 | >25 |
| White cell count: depressed (10^9^/L) | 2.50 – 3.50 | 1.50 – 2.49 | 1.00 – 1.49 | <1.0 |
| Neutrophil count (10^9^/L) | 1.5-2.0 | 1.0-1.4 | 0.5-0.9 | <0.5 |
| Platelets (10^9^/L) | 125-140 | 100-124 | 25-99 | <25 |
| Sodium: hyponatremia (mmol/L) | 132–134 | 130–131 | 125–129 | <125 |
| Sodium: hypernatremia (mmol/L) | 146 | 147 | 148–150 | >150 |
| Potassium: hyperkalemia (mmol/L) | 5.4 – 5.5 | 5.6 – 5.7 | 5.8 – 5.9 | >5.9 |
| Potassium: hypokalemia (mmol/L) | 3.3–3.4 | 3.1–3.2 | 3.0 | <3.0 |
| Urea (mmol/L) | 8.2–8.9 | 9.0–11 | >11 | RRT |
| Creatinine (μmol/L) | 132-150 | 151-176 | 177-221 | >221 or RRT |
| ALT and/or AST (IU/L) | 1.1–2.5 x ULN | >2.6–5.0 x ULN | 5.1-10 x ULN | >10 x ULN |
| Bilirubin, with increase in LFTs (μmol/L) | 1.1–1.25 x ULN | 1.26–1.5 x ULN | 1.51–1.75 x ULN | >1.75 x ULN |
| Bilirubin, with normal LFTs (μmol/L) | 1.1–1.5 x ULN | 1.6–2.0 x ULN | 2.1–3.0 x ULN | >3.0 x ULN |
| ALP (IU/L) | 1.1–2.0 x ULN | 2.1–3.0 x ULN | 3.1–10 x ULN | >10 x ULN |
| Albumin: hypoalbuminemia (g/L) | 28–31 | 25–27 | <25 | Not applicable |
| C-reactive protein | >10-30 | 31-100 | 101-200 | >200 |

**Table 5B: Grading of laboratory adverse events.**

Abbreviations: ALT, alanine aminotransferase; AST, aspartate aminotransferase; ALP, alkaline phosphatase; ULN, upper limit of normal; LFTs, liver function tests
